# Formation of persistent organic diradicals from *N*,*N′*-diphenyl-3,7-diazacyclooctanes

**DOI:** 10.1007/s00706-018-2298-4

**Published:** 2018-10-29

**Authors:** Sara Norrehed, Christoffer Karlsson, Mark E. Light, Anders Thapper, Ping Huang, Adolf Gogoll

**Affiliations:** 1 Department of Chemistry - BMC, Uppsala University, Uppsala, Sweden; 2 Department of Engineering Sciences, Uppsala University, Uppsala, Sweden; 3 Department of Chemistry, University of Southampton, Highfield, Southampton, UK; 4 Department of Chemistry - Ångström Laboratory, Uppsala University, Uppsala, Sweden

**Keywords:** Biaryls, Crystal structure, Heterocycles, Oxidative coupling

## Abstract

**Abstract:**

*N*,*N′*-Diphenyl-3,7-diazacyclooctane and structurally related *N*,*N′*-diphenylbispidine derivatives react with silver(I) ions in a high-yielding C–C coupling reaction to produce dication–diradical species, with the silver ions serving a double function both as template and as an oxidant. The resulting bis(benzidino)phane derivatives are persistent organic radicals, stable for several months in solution as well as in the solid state, at room temperature and above, as well as being exposed to the atmosphere. The molecular structure features a double-decker cyclophane motif, stabilized by intramolecular π-dimerization of two delocalized benzidinium radical segments. Intermolecular π-dimers are formed in the solid state.

**Graphical abstract:**

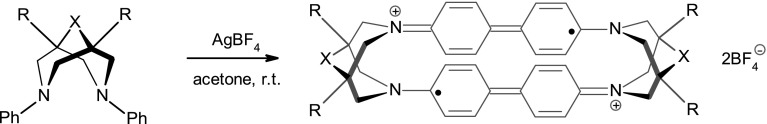

**Electronic supplementary material:**

The online version of this article (10.1007/s00706-018-2298-4) contains supplementary material, which is available to authorized users.

## Introduction

Persistent organic diradicals are of interest for a variety of applications as functional materials, such as molecular magnets and molecular electronics [1–6]. In contrast to monoradicals, they offer the intriguing option of modulating their spin state. Diradicals with intramolecular π−π interactions are of particular interest, and only a few examples have been reported [4]. Common structural motifs are based on the dimers of benzidine [7] (4,4′–diaminobiphenyl) or *N*,*N*,*N*′,*N*′-tetraaryl-(1,1′-biphenyl)-4,4′-diamines [8–10]. While these usually retain some structural flexibility regarding the π−π-interacting segments, attempts have been made to obtain “confronted arenes” [11] with closer interactions, such as found in cyclophane-like structures [12]. We here report the serendipitous finding of a simple synthetic method to produce highly persistent organic diradicals with strong intramolecular π−π interactions.

## Results and discussion

During preparation of π-allyl palladium complexes with the chelating nitrogen ligand *N*,*N*′-diphenylbispidine (**2**) (Scheme 1) in the presence of silver salts, we occasionally noted a greenish discoloration [13]. Eventually, we found that stoichiometric conversion of bispidines to intensely green products requires just the presence of an *N*,*N*′-diphenyl-3,7-diazacyclooctane (**1**) skeleton as well as silver (Ag^+^) ions. Thus, bispidines **1**–**3** show this reactivity, giving dark green solids in 87% (**5**, from **1**) and 73% (**6**, from **2**) yield after HPLC purification, respectively (Scheme 1). Bispidine **3** produces a mixture. In contrast, *N*,*N*′-diphenylpiperazine (**4**) forms a colourless precipitate. Further investigations using pseudo-Job plots [14] reveal a 1:3 (diamine:Ag^+^) stoichiometry of this reaction (Supplementary data Fig. S14). The isolation of elemental silver as a by-product, typically obtained as silver foil (Supplementary data Fig. S8), suggested a redox reaction between a phenyl amine and Ag^+^ involving one-electron transfer and the formation of organic radicals, similar to those obtained in oxidative coupling of anilines to benzidines, and also supported by the intense green colour [14].

**Figure:**
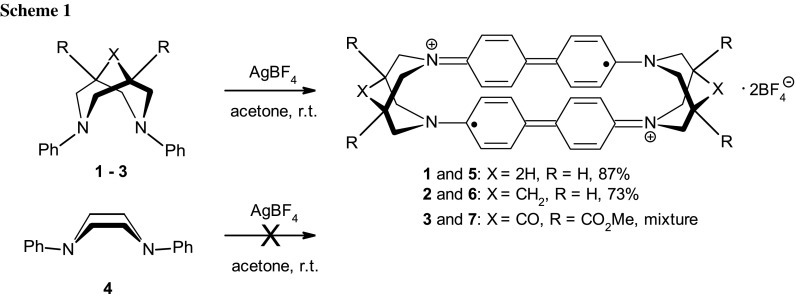

The structure of **5** was revealed by X-ray crystallography. In the crystals, two benzidine units are arranged into a cyclophane motif, and intercalated with counterions and solvent molecules. To balance the counterion charge (i.e., 2 × BF_4_^−^), a dicationic organic component is required. This would be possible if each benzidine unit carried one unpaired electron (Scheme 1).

**Fig. 1:**
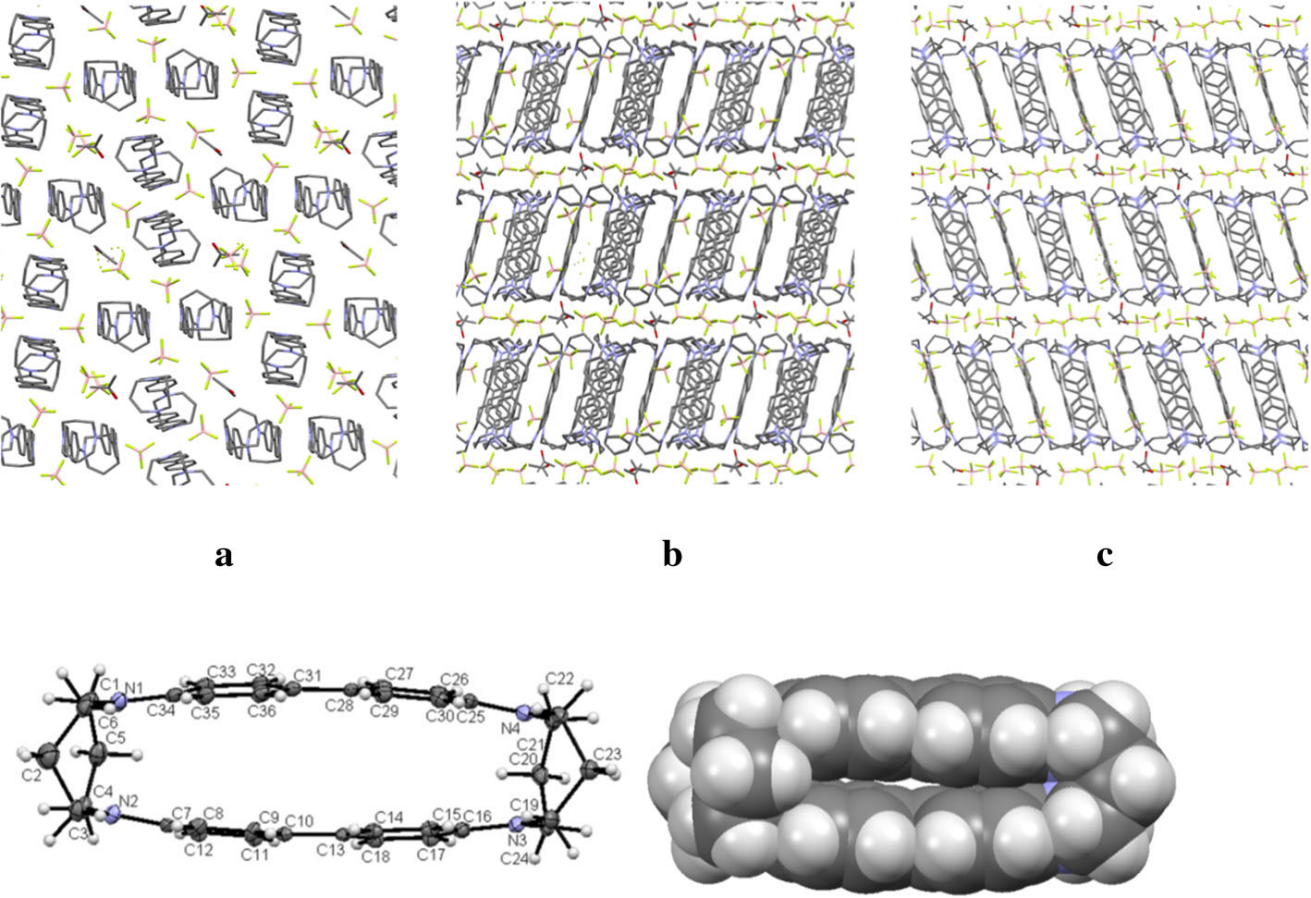
Crystal structure of dication–diradical **5** confirms double-decker cyclophane motif. Top: The arrangement of dication–diradical cyclophanes displayed along the *a*, *b*, and *c*-axes, showing intercalation with counterions and solvent molecules (crystal from acetone solution). Bottom left: ORTEP figure, ellipsoids drawn at 35% probability level, showing slight deviation of the two benzidine segments from perfect planarity. Bottom right: atoms drawn at van der Waals radii, indicating the close π-stacking between the two cyclophane segments

Two different morphologies were obtained depending on the solvent used for crystallization; both being composed of double-decker cyclophanes incorporating two benzidine units (Fig. 1).

**Fig. 2:**
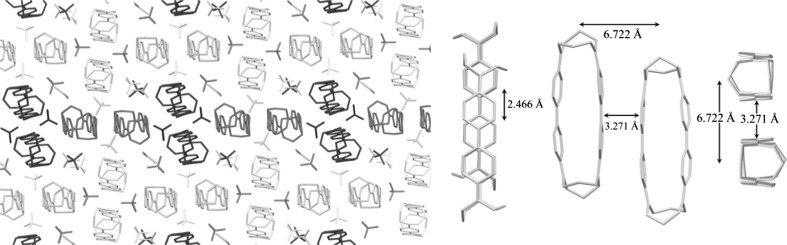
Intra- and intermolecular π-stacking in the solid state of dication–diradical **5**. Left: crystal packing viewed down crystallographic a-axis, with colour coding of symmetry equivalent units of pairwise π-stacked cyclophanes. Right: intermolecular π-stacking within a pair of symmetry equivalent molecules, viewed from three different directions

The molecular structure of the cyclophane units is characterized by two almost parallel biphenyl segments, separated by approximately 3.16 Å [terminal carbons C(7)–C(34) at 3.175(6) Å and C(16)–C(25) at 3.151(5) Å] to 3.56 Å [central carbons C(10)–C(31) at 3.560(5) Å and C(13)–C(28) at 3.555(5) Å]. This distance is similar to that found for π-dimers of persistent cation radicals in the solid state, which is 2.9–3.4 Å [1]. Spacing between the pairs of terminal nitrogen atoms is somewhat closer, at 2.745(5) Å (N(1)–N(2)) and 2.711(5) (N(3)–N(4)), resulting in a slight deviation of the biphenyl segment from planarity. Within each biphenyl segment, the two phenyl rings are almost in the same plane, with torsion angles of 177.8(4)° (C(9)–C(10)–C(13)–C(18)) and 179.1(4)3° (C(32)–C(31)–C28)–C(29). For the nitrogen atoms, bond angles close to 120° indicate sp^2^-hybridization, e.g., for N(1): C(1)–N(1)–C(6) = 116.2(4)°, C(34)–N(1)–C(6) = 120.9(4)°, and C(34)-N(1)-C(1) = 122.9(4)°. This hybridization is further supported by finding each nitrogen atom and the three bonded carbon atoms at very close distance [varying from − 0.0054(0.0033) Å for N(16) to 0.0429(0.0029) Å for N(7)] to the mean plane of these four atoms. Notably, in contrast to most intermolecular π-dimers, the phenyl rings of the two benzidine units are aligned on top of each other, without any lateral displacement. In addition, intermolecular π–stacking is also present, constituted by pairwise arrangement of molecules at 3.271 Å distance between benzidine segments with lateral displacement of ca. 2.466 Å (Fig. 2). It should be emphasized that there are only a few other reported X-ray crystallographic data for benzidine cation radicals [1, 15].

Bond lengths in the benzidine segments of **5** deviate from those found in benzidine itself (Fig. S16, Supplementary), as would be expected for a quinoidal resonance structure, similar to recently reported benzidine cation radicals [9].

While the dication–diradical nature of **5**–**7** initially was inferred from the structure of **5** as revealed by X-ray crystallography (vide supra), several further observations support its electronic structure. First, the presence of unpaired electrons results in ^1^H NMR spectra of the isolated products **5**–**7** with very broad signals revealing their paramagnetic properties (Fig. S6, Supplementary). However, it was possible to obtain ^19^F NMR spectra with reasonably narrow signals for their BF_4_^−^ counterions, due to some distance from the paramagnetic centers [16]. Interestingly, diffusion coefficients determined by LED–PGSE NMR experiments on ^19^F corresponded well to the hydrodynamic radii of the proposed dimeric reaction products (Table S1, Supplementary) when relating these parameters via the Stokes–Einstein equation [17]. This indicates the presence of tight ion pairs. Notably, for *N,N′*-diphenylpiperazine (**4**), which showed entirely different reactivity, the ^19^F detected BF_4_^−^ diffusion coefficient remained essentially the same as that for an AgBF_4_ solution.

Second, the UV/Vis spectrum of dication–diradical **5** shows absorption maxima at 403 and 760 nm (Fig. S11, Supplementary), bands that have been assigned to cation radical dimers in other benzidines, whereas the bands at higher wavelengths (typically *λ* ≥ 800 nm), assigned to cation radical monomers [8, 14, 18], are completely absent. Interestingly, the dark green solid of **5**, obtained by drying at atmospheric pressure, reversibly can be transformed into a black solid under vacuum, indicating a solvatochromic effect [19] due to removal of solvent molecules from the crystal lattice.

Third, upon exposure to reducing agents such as bisulfite or hydroquinone, solutions of the dication–diradical **5** immediately turned colourless, and presence of the diamagnetic cyclophane **8** (Scheme 2), having some resemblance to Stetter cyclophanes [20, 21], could be proven by ^1^H NMR spectra and high-resolution mass spectra.

**Figure:**
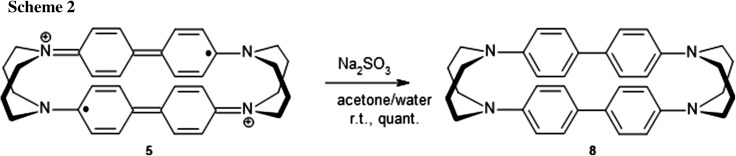

**Fig. 3:**
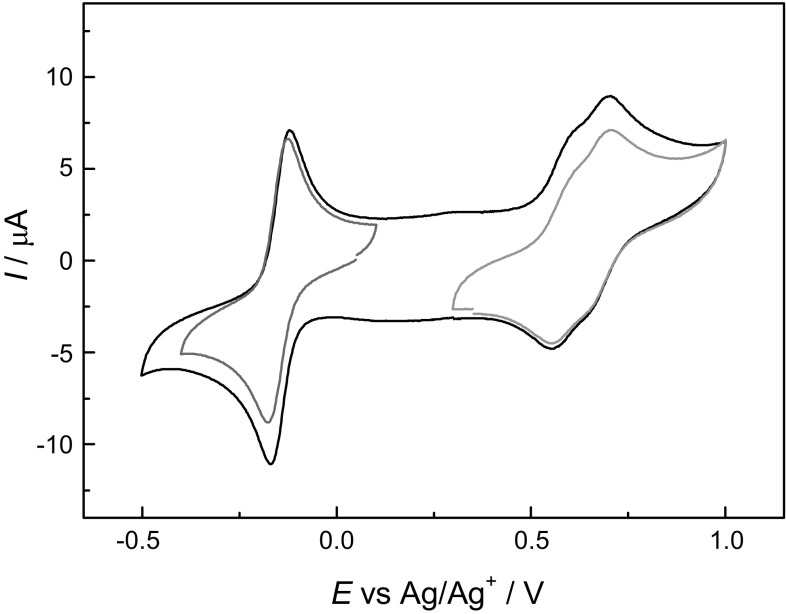
Cyclic voltammetry showing several redox steps for the dication–diradical cyclophane **5**

Fourth, cyclic voltammetry (Fig. 3) revealed that compound **5** is reversibly reduced at *E*_A_^0^ = − 0.144 V vs. Ag/Ag^+^, and it is reversibly oxidized in two steps at *E*_B_^0^ = 0.580 and *E*_C_^0^ = 0.683 V vs. Ag/Ag^+^, a behavior that would be expected for the proposed dication–diradical structure.

The charge passed at *E*_A_^0^ (blue curve in Fig. 3) is comparable to the total charge passed at *E*_B_^0^ and *E*_C_^0^ (green curve in Fig. 3), and the two latter peaks are of the same size. This is consistent with a two-electron transfer in process A, and with processes B and C being two consecutive one-electron transfers.

Process A is thus the reduction of **5** to the neutral species **8**, also obtained by chemical reduction, while processes B and C are oxidations to the tri- and tetracations, respectively. The benzidine units likely adopt a quinonoid structure upon oxidation, which would make the tetracation spinless.

Reduction of compound **6** is quasireversible, while its oxidation is irreversible. Both reduction and oxidation of compound **7** are irreversible under the applied conditions. Even though the dications of **6** and **7** are extremely stable, and the oxidation of **5** is reversible; the tri- and tetracations of **6** and **7** are, hence, very unstable in solution. Electrochemical redox reactions of benzidine derivatives have previously been reported, with the redox potentials being very solvent-dependent [22, 23].

Final support for the presence of radical species is provided by the detection of EPR signals from **5** (Supplementary Fig. S15). Thus, for the solid material **5**, an axial EPR signal with two *g* values (*g*| = 2.0040 and *g*_⊥_ = 2.0025, respectively) is observed. This signal is similar to the reported EPR signal of single-electron oxidized 3,3′-dimethyl-4,4′-diaminobiphenyl [18]. In a frozen dimethylsulfoxide solution, the EPR signal is broad (*g* = 2.004) and does not show any hyperfine splittings, indicating spin-exchange interactions between the unpaired electrons of the two closely spaced benzidine units [24, 25]. Further indication of interaction between the two biphenyl units of **5** via π-pairing, i.e., intramolecular formation of π-dimers, is provided by the relative weakness of the EPR signals. The formation of intermolecular diamagnetic π-dimers has, otherwise, been reported for some cation radicals at low temperatures [26, 27]. This fits well with the molecular structure described above.

The temperature dependence of the magnetic susceptibility of **5** was studied over the temperature range 2–150 K at a magnetic field strength of 0.1 T (Supplementary Fig. S15c). The molar susceptibility increases continuously with decreasing temperature in the measured temperature range, indicating a paramagnetic spin-triplet ground state. This spin state is converted into a diamagnetic singlet state at ~ 160 K. Hence, the energy difference between these two spin states must be small, with a rather weak intramolecular spin-exchange interaction. The previously mentioned pairwise intermolecular π-stacking effect is featured in the Curie–Weiss plot (Supplementary Fig. S15d) exhibiting a positive Weiss temperature ($\varTheta > 0)$, showing the presence of antiferromagnetic intermolecular interaction in the material.

With the structure of the novel persistent organic diradicals formed from *N*,*N*′-diphenyldiazacyclooctanes having been determined, it remained to propose a reasonable mechanism for their formation (Scheme 3). As bispidines are strong chelating ligands, it is likely that, initially, a complex is formed, followed by a redox reaction releasing Ag^0^. The resulting cation radical **1a** dimerizes, followed by deprotonation of the dication **1b** to the neutral tetraamine **1c**. This intermediate has two chelating sites, each binding one Ag^+^ ion, thus triggering two further redox reactions, yielding **1d**. Due to spatial proximity of the two radical units, dication–diradical **1d** favours intramolecular dimerization to the cyclophane ring of **1e**, rather than intermolecular dimerization. Therefore, the overall reaction has high yields and polymeric by-products are not an issue. Deprotonation transforms **1e** into the closed shell product **8**, a new chelating tetraamine. This binds two further Ag^+^ ions, followed by oxidation to dication–diradical **5**. In contrast to the previous cation radicals **1a** and **1d**, dication–diradical **5** is stabilized by delocalization involving the entire biphenyl system, and does, therefore, not react further. Overall, two diamine molecules (**1**–**3**) consume six Ag^+^ ions to form the cyclophane dication–diradicals **5**–**7**, as can be shown by screening reactions to produce pseudo-Job plots (Supplementary material). Besides being a one-electron oxidant, the Ag^+^ also serves a crucial template function in this reaction. Efficient binding of Ag^+^ to the intermediate **1c** due to a chelate effect is likely to give a high concentration of diradical **1d**, which then favours intramolecular cyclization over intermolecular oligomerization. This also explains the less clean reaction of bispidinone **3**, as it is known that bispidinones are weaker electron pair donors than bispidines [28]. Thus, weaker binding to Ag^+^ is likely, allowing for a higher extent of intermolecular oligomerization to produce a mixture of products.

**Figure:**
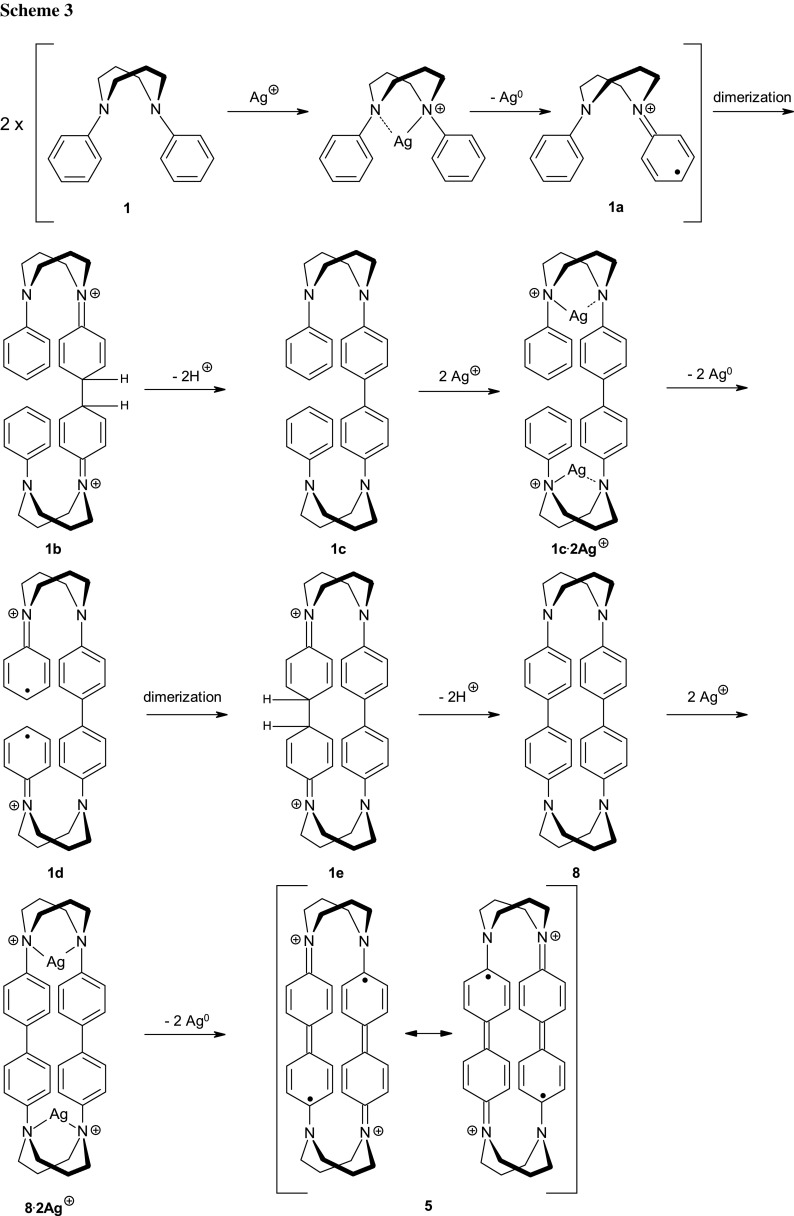

This mechanism is in line with one discussed by Bondarchuk and Minaev for the oxidative coupling of anilines [29]. More generally, it is related to the oxidative dimerization of arylamines to benzidines with Cu^2+^ or iron oxidants [22, 30, 31]. Furthermore, it also has some resemblance with the oxidative para-coupling of phenols, resulting in 4,4′-dihydroxybiphenyls, also involving radicals [32, 33]. However, the selective metal chelate-directed cyclization that we here propose to be the reason for the formation of the bisbenzidine motif, rather than polymers, in high yield has not been observed before.

Finally, the strikingly different reactivity of Ag^+^ with *N,N*′-diphenylpiperazine (**4**) calls for an explanation. This ligand enables the formation of a five-membered chelate ring, and with an ionic radius of 1.26 Å [33] Ag^+^ is expected to bind more strongly to the diazacyclohexane ligand **4** than to the diazacyclooctane ligands **1**–**3** and their subsequent reaction products, as shown in Scheme 3 [35–38]. We, therefore, believe steric factors to be crucial, since the cyclization step (**1d** → **1e**, Scheme 3), resulting eventually in the formation of stable diradicals with planar sp^2^ geometry at all four nitrogens (i.e., in **5**, including resonance effects), is not possible for the piperazine backbone. Hence, persistent radical species are not formed from **4**.

## Conclusion

In conclusion, a selective oxidative cyclization between *N,N*′-diphenyl-3,7-diazacyclooctane or structurally related bispidines and Ag^+^ ions provides rapid access to cyclophanes incorporating two benzidine segments. These compounds are obtained as highly persistent dication–diradicals with both intramolecular spin interactions via π-pairing, as well as intermolecular π-pairing in the solid state.

## Experimental

UV–Vis measurements were carried out on a Varian Cary 3 Bio spectrophotometer using 10 or 5 mm quartz cuvettes at r.t. with acetone as solvent. Purification by preparative HPLC was performed on a Gilson 231 system connected to an ACE AQ C18 (10 µm, 100 × 21 mm) column using a gradient of MeCN/H_2_O with 0.1% TFA, flow rate 10 cm^3^ min^−1^, and detection at 254 nm or 760 nm. NMR investigations were carried out on a Varian Unity Inova (^1^H at 499.94 MHz, ^19^F at 470.34 MHz, ^13^C at 125.7 MHz) spectrometer. Chemical shifts are reported in ppm referenced to tetramethylsilane via the residual solvent signal (CDCl_3_, ^1^H at 7.26 and ^13^C at 77 ppm; acetone-*d*_*6*_, ^1^H at 2.05 and ^13^C at 39.5 ppm). ^19^F chemical shifts were referenced to external CF_2_Br_2_ (7.0 ppm). Signal assignments were derived from P.E.COSY [39], gHSQC [40], gHMBC [41], gNOESY [42], ROESY [43], and TOCSY [44] spectra. *D* (diffusion coefficients) were determined from LED–PGSE experiments [45, 46], using z-gradients, acquiring a series of 10–20 spectra for an array of gradient pulse strengths (0–20 gauss/cm). Typically, a relaxation delay of 1 s, 9 ms gradient pulse duration, 100 ms diffusion delay, and 5 ms storage delay were used. The LED–PGSE spectra were evaluated by plotting the square of the gradient strength against the natural logarithm of the signal amplitude, resulting in a straight line with a slope proportional to − *D*. The actual value for *D* was obtained by relating this slope to that of a compound with known *D*, measured under the same conditions. In the present investigation, we have used KF in H_2_O (*D* = 1.14 × 10^−9^ m^2^ s^−1^) [47]. Melting points were determined using a Stuart Scientific melting point apparatus SMP10. Commercially available compounds were used without purification. HRMS was acquired using a Thermo Scientific LTQ Orbitrap Velos apparatus in infusion mode. An Autolab PGSTAT302 N potentiostat (Ecochemie, The Netherlands) was used for electrochemical measurements. The analytes were dissolved to 0.50 mM in dry acetonitrile (MeCN) with 0.1 M tetrabutylammonium hexafluorophosphate (TBAHFP) supporting electrolyte. A polished glassy carbon (GC) disk electrode (3.0 mm diameter) was used as working electrode, a Pt wire as counter electrode, and the reference electrode consisted of a Ag^0^/Ag^+^ electrode (10 mM AgNO_3_, 0.1 M TBAHFP, − 0.096 V vs. Fc^0^/Fc^+^) that was kept in a separate compartment. The electrolyte was thoroughly degassed with solvent-saturated N_2_ (g) and kept under N_2_ (g) atmosphere throughout the measurements. Formal potentials were determined as the average of cyclic voltammetry (CV) oxidation and reduction peak potentials.

The EPR spectra of **5** were recorded on a Bruker EMXmicro spectrometer using an ER 4119HS resonator (solid sample) and a Bruker E500-ELEXSYS spectrometer using an ER 4122SHQE resonator equipped with ESR900 cryostat and an Oxford ITC503 temperature controller (frozen solution sample).

Magnetic susceptibility data of **5** in a powder sample were measured on a SQUID magnetometer (Quantum Design MPMSXL-5) as a function of temperature between 2 and 160 K at the magnetic field of 0.1 T. Diamagnetic corrections were determined from Pascal’s constants and background subtrated in RSO-operating mode.

### Synthetic procedures

Synthesis of *N*,*N*′-diphenyl-1,5-diazacyclooctane (**1**) was carried out according to the literature procedures with some modifications [48] (see the Supplementary data for details). *N*,*N*′-diphenyl-3,7-diazabicyclo[3.3.1]nonane (**2**) [28], *N*,*N*′-diphenyl-1,5-dicarbomethoxybispidinone (**3**) [48], and *N*,*N*′-diphenylpiperazine (**4**) [48] were prepared according to the literature procedures.

#### 1,8-Diaza-4,11-diazaniumyl-2,3,9,10(1,4)-tetrabenzenatricyclo[9.3.3.3^4^,^8^]eicosaphane bis(tetrafluoroborate) (5, C_36_H_40_N_4_)

*N*,*N***′**-Diphenyl-1,5-diazacyclooctane (**1**, 195 mg, 0.73 mmol) in 4 cm^3^ acetone was added to a solution of 0.44 g AgBF_4_ (1.15 mmol, 3.1 eq.) in 4 cm^3^ acetone. Directly upon mixing the combined solutions turned to an intense dark green colour and a silver mirror started to form on the inside of the vial within seconds. After standing for 48 h (r.t., dark), purification of the reaction mixture was possible by preparative HPLC achieving excellent separation and allowing isolation of the product (Fig. S7). Evaporation of solvents yielded compound **5** as a dark green solid (224 mg, 0.31 mmol, 87%). It should be noted that **5** was stable at r.t. both as a solid as well as in solution for several months. M.p.: 208–210 °C; ^1^H NMR (500 MHz, acetone-*d*_*6*_, 25 °C): *δ* = 6.26 (broad, *w* = 367.3 Hz) ppm; ^19^F NMR (470.34 MHz, acetone-*d*_*6*_, 25 °C): *δ* = − 147.0 ppm; UV/Vis (acetone, r.t.): *λ* = 325, 403, 760 nm; HRMS: [M]^2+^ calcd. for C_36_H_40_N_4_ *m/z* = 264.1621, found 264.1593.

#### 1,4,8,11-Tetraaza-2,3,9,10(1,4)-tetrabenzenatricyclo[9.3.3.3^4^,^8^]eicosaphane (8, C_36_H_40_N_4_)

Dication–diradical **5** (2 mg, 0.0029 mmol) was dissolved in 0.5 cm^3^ acetone and 0.5 cm^3^ of a saturated solution of Na_2_SO_3_ in H_2_O was added dropwise during stirring until the dark green solution turned pale brown. Extraction with CHCl_3_ and evaporation of solvents afforded the bis(benzidino)phane **8** as an oily beige solid (2 mg, quant). ^1^H NMR (500 MHz, CDCl_3_/D_2_O/acetone-*d*_*6*_ 1:1:1, 25 °C): *δ* = 6.62 (d, *J* = 8.6 Hz, 8H, Ar), 6.05 (d, *J* = 8.6 Hz, 8H, Ar), 3.78 (dm, *J* = 15.2 Hz, 8H, CH_2_), 3.13–2.84 (m, 16 H, CH_2_) ppm; UV/Vis (acetone, r.t.): *λ* = 325 nm; HRMS: [M+H]^+^ calcd. for C_36_H_40_N_4_ *m/z* = 529.3315, found 529.3321.

#### 1,8-Diaza-4,11-diazaniumyl-2,3,9,10(1,4)-tetrabenzenatricyclo[9.3.3.3^4,8^.1^6,19^.1^13,16^]docosaphane bis(tetrafluoroborate) (6, C_38_H_40_BF_4_N_4_)

3,7-Diphenyl-3,7-diazabicyclo[3.3.1]nonane **2** (50 mg, 0.18 mmol) in 1 cm^3^ acetone was added to a solution of 105 mg AgBF_4_ (0.54 mmol, 3.1 eq.) in 1 cm^3^ acetone. Directly upon mixing the combined solutions turned into an intense dark green colour and a silver mirror started to form on the inside of the vial within minutes. After standing for 48 h (r.t., dark), purification of the reaction mixture with preparative HPLC afforded the isolation of the product **6** that, after evaporation of solvents, was obtained as a dark green solid (48 mg, 0.07 mmol, 73%). M.p.: 120 °C (dec.); ^1^H NMR (500 MHz, acetone-*d*_*6*_, 25 °C): *δ* = 3.11 (broad, *w* = 182.1 Hz) ppm; ^19^F NMR (470.34 MHz, acetone-*d*_*6*_, 25 °C): *δ* = − 147.1 ppm; UV/Vis (acetone, r.t.): *λ* = 223, 323, 394, 751 nm; HRMS: [M+BF_4_]^+^ calcd. for C_38_H_40_BF_4_N_4_ *m/z* = 639.3271, found 639.3264.

#### 6,13,16,19-Tetracarbomethoxy-1,8-diaza-4,11-diazaniumyl-2,3,9,10(1,4)-tetrabenzenatricyclo[9.3.3.3^4^,^8^.1^6^,^19^.1^13^,^16^]docosaphane-21,22-dione bis(tetrafluoroborate) (7, C_46_H_47_N_4_O_10_)

3,7-Diphenyl-1,5-dicarbomethoxybispidinone **3** (50 mg, 0.12 mmol) in 1 cm^3^ acetone was added to a solution of 71.5 mg AgBF_4_ (0.37 mmol, 3.1 eq.) in 1 cm^3^ acetone. Directly upon mixing the combined solutions turned to a dark brown that within hours shifted into deep green. After standing (r.t., dark) for 48 h, a silver flake had formed on the inside of the vial. The flake could be removed from the flask as a single piece of Ag(s) (see Supplementary for image, Fig. S8). HPLC analysis of the reaction mixture proved a complex mixture and the product **7** could not be fully isolated. ^1^H NMR (500 MHz, acetone-*d*_*6*_, 25 °C, crude mixture): *δ* = 4.70 (broad, *w* = 115.5 Hz) ppm; HRMS: [M +3H]^+^ calcd. for C_46_H_47_N_4_O_10_ *m/z* = 815.3292, found 815.3279; analysis (calcd., found for Ag flake): Ag (100.0, 100.0); UV/Vis (acetone, r.t., crude): *λ* = 332, 389, 746 nm.

### X-ray crystallography data

CCDC 1850267 and CCDC 1850282 contain the crystallographic data for compound **5**. These data can be obtained free of charge from the Cambridge Crystallographic Data Centre via www.ccdc.cam.ac.uk/data_request/cif.

## Electronic supplementary material

Below is the link to the electronic supplementary material.

Supplementary material 1 (PDF 1454 kb)
