# Supplementary material for: Formation of persistent organic diradicals from N,N′-diphenyl-3,7-diazacyclooctanes
Source: Monatsh Chem. 2018 Oct 29;150(1):77–84. doi: 10.1007/s00706-018-2298-4 (PMC6320754; doi:10.1007/s00706-018-2298-4)
Supplement: Supplementary file 1 — Supplementary material 1 (PDF 1454 kb) [file 706_2018_2298_MOESM1_ESM.pdf]

Supplementary material
Formation of persistent organic diradicals from N,N'-diphenyl-3,7-diazacyclooctanes

Sara Norrehed[1], Christoffer Karlsson[2], Mark E. Light[3], Anders Thapper[4], Ping Huang[4] and Adolf Gogoll[1]

[1]*Department of Chemistry-BMC, Uppsala University, S-75123 Uppsala, Sweden*

[2]*Department of Engineering Sciences, Uppsala University, S-751 21 Uppsala, Sweden*

[3]*Department of Chemistry, University of Southampton, Highfield, Southampton SO17 1BJ, U.K.*

[4]*Department of Chemistry – Ångström Laboratory, Uppsala University, S-75120 Uppsala, Sweden*

*e-mail: adolf.gogoll@kemi.uu.se

1 Synthetic route to *N,N′*-diphenyl-1,5-diazacyclooctane 1 2

1.1 1,3-Ditosylamido-propane 1a[15] 2

1.2 1,3-Ditosyloxy-propane 1b[16] 3

1.3 *N,N′*-Ditosyl-1,5-diazacyclooctane 1c[9] 4

1.4 1,5-Diazacyclooctane dihydrobromide 1d[9] 5

1.5 N,N'-Diphenyl-1,5-diazacyclooctan 1[10] 6

2 1,8-Diaza-4,11-diazaniumyl-2,3,9,10(1,4)-tetrabenzenatricyclo[9.3.3.3^{4,8}]eicosaphane bis(tetrafluoroborate) 5 7

3 1,4,8,11-tetraaza-2,3,9,10(1,4)tetrabenzena-tricyclo[9.3.3.3^{4,8}]eicosaphane 8 .. 8

4 1,8-Diaza-4,11-diazaniumyl-2,3,9,10(1,4)tetrabenzena-tricyclo[9.3.3.3^{4,8}.1^{6,19}.1^{13,16}]docosaphane bis(tetrafluoroborate) 6 10

5 6,13,16,19-Tetracarbomethoxy-1,8-Diaza-4,11-diazaniumyl-2,3,9,10(1,4)tetrabenzena-tricyclo[9.3.3.3^{4,8}.1^{6,19}.1^{13,16}]docosaphane-21,22-dione bis(tetrafluoroborate) 7 11

6 Job plots for formation of dication diradicals 5, 6 and 7 12

7 Diffusion coefficients for the discussed species 13

8 EPR and magnetic susceptibility data 14

9 X-ray crystallography data 15

9.1 Crystal from acetone-water solution after purification by HPLC 15

9.2 Crystal from acetone solution 39

10 References 61

1 Synthetic route to *N,N′*-diphenyl-1,5-diazacyclooctane 1

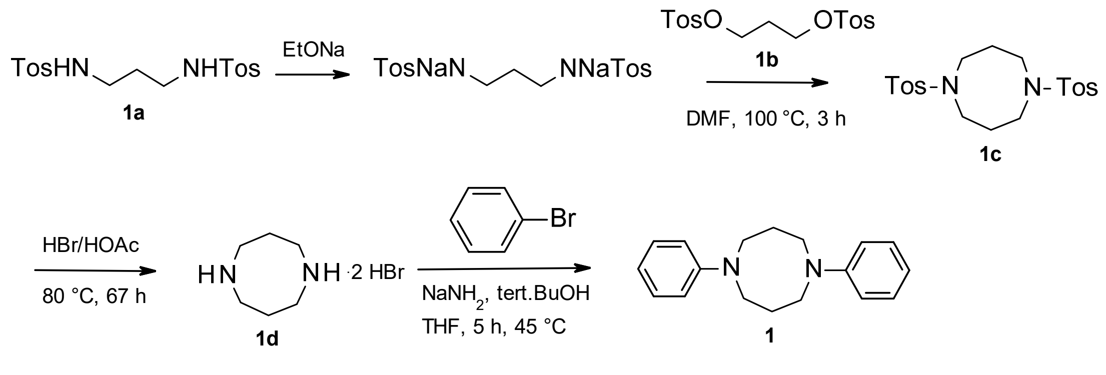

Scheme S1. Synthesis of *N,N′*-diphenyl-1,5-diazacyclooctane **1**.

1.1 1,3-Ditosylamido-propane 1a[15]

In a 250 mL round-bottom flask equipped with a magnetic stir bar and gas lock, 1,3-diaminopropane (6.8 g, 92 mmol) was added to dry pyridine (80 mL) and the solution was cooled to °C. p-Toluenesulfonyl chloride (40 g, 206 mmol) was added in portions during 1.5 h to keep the temperature below 5°C. Pyridine hydrochloride started to precipitate after the first addition, and the solution turned yellow. Stirring at 0°C was continued for 4.5 h. Water (100 mL) and conc. hydrochloric acid (ca. 20 ml) was added. The precipitated yellow solid was filtered off on a Büchner funnel and washed with 3 portions of water. After drying on air, **1a** was obtained as a yellow powder, 18.62 g (48.7 mmol, 53%), m.p. 137°-140°C.
^{1}H NMR ($CDCl_3$) δ: 7.72 (AA'XX', 4H, Tos), 7.30 (AA'XX', 4H, Tos), 5.06 (t, J=6.6 Hz, 2H, NH), 3.00 (q, J=6.4 Hz, 4H, CH_2-N), 2.42 (s, 6H, Me-Ph), 1.66 (q, 2H, CH_2).
^{13}C NMR ($CDCl_3$) δ: 143.5, 136.7, 129.8, 127.0, 39.8, 29.8, 21.5.

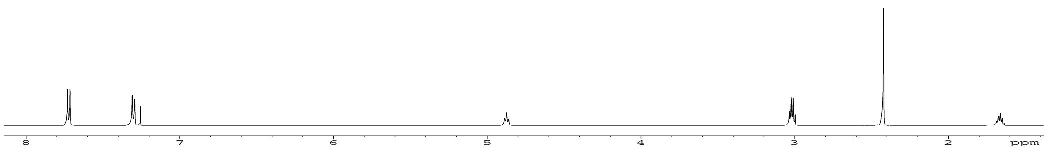

Figure S1. ^{1}H NMR spectrum (500 MHz, $CDCl_3$ solution, 25°C) of 1,3-ditosylamido-propane **1a**.

1.2 1,3-Ditosyloxy-propane 1b[16]

In a 250 mL round-bottom flask fitted with a reflux condenser and gas lock, 1,3-propanediol (7.6 g, 100 mmol) was dissolved in THF (75 mL). NaOH (14 g, 350 mmol) was dissolved in THF (75 mL) and added to the solution which was then cooled to 0 °C. p-Toluenesulfonylchloride (42 g, 220 mmol) was dissolved in THF (75 mL) and added dropwise over 1 h and the oily white mixture was left to stir for 3 h. HCl (10 %) was cooled to 0 °C and added in portions until no more precipitate formed. The white particles were filtered off and washed with H_2O and sat. $NaHCO_3$. Recrystallization from EtOAc/MeOH yielded the product as white needle-shaped crystals (19.6 g, 51 %).

^{1}H NMR ($CDCl_3$) δ: 7.75 (AA'XX', 4H, Tos), 7.35 (AA'XX', 4H, Tos), 4.07 (t, J=6.0 Hz, 4H, CH_2O), 2.46 (s, 6H, Me-Ph), 2.00 (q, J=6.0 Hz, 2H, CH_2).

^{13}C NMR ($CDCl_3$) δ: 145.0 (ipso), 132.6, 130.0, 127.9, 65.8, 28.7, 21.7.

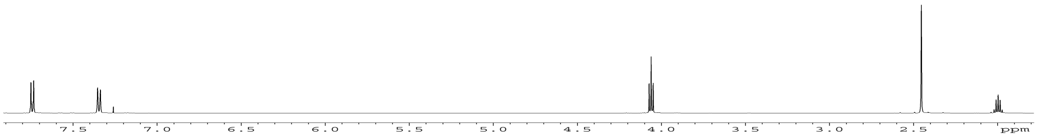

Figure S 2. ^{1}H NMR spectrum (500 MHz, $CDCl_3$ solution, 25°C) of 1,3-ditosyloxy-propane **1b**.

1.3 *N,N′*-Ditosyl-1,5-diazacyclooctane 1c[9]

EtONa (3.6 g, 52.4 mmol) is dissolved in 100 mL EtOH in a 500 mL round-bottomed flask. 1,3-Ditosylamido-propane **1a** (10.0 g, 26.2 mmol) was added and a milky peach-coloured mixture formed. The solvent was evaporated and DMF (dry, 230 mL) was added to the formed solid to give a bright orange solution. 1,3-ditosyl-oxy-propane **1b** (10.0 g, 26.2 mmol) in DMF (130 mL) was added and the mixture was refluxed at 100 °C for 3 h. When the mixture had cooled to room temperature it was transferred to a 1000 mL E-flask and H_2O added (500 mL) (Heat evolves). The product was formed as a white fluffy solid formed which was filtered off and washed with H_2O (500 mL) in several small portions and dried on air. (8.1 g, 19 mmol, 73 %) m.p. 215-222°C (Lit. 214-215°C).[9]

^{1}H NMR ($CDCl_3$) δ: 7.68 (AA'XX', 4H, *o*-Tos), 7.31 (AA'XX', 4H, *m*-Tos), 3.27 (m, 8H, CH_2N), 2.43 (s, 6H, Tos-CH_3), 2.04 (m, 4H, CH_2). ^{13}C NMR ($CDCl_3$) δ: 143.3 (*o*-Tos), 135.5 (*ipso*-Tos), 129.7 (*m*-Tos), 127.0 (*o*-Tos), 46.9 (CH_2-N), 30.1 (CH_2), 21.4 (Tos-CH_3).

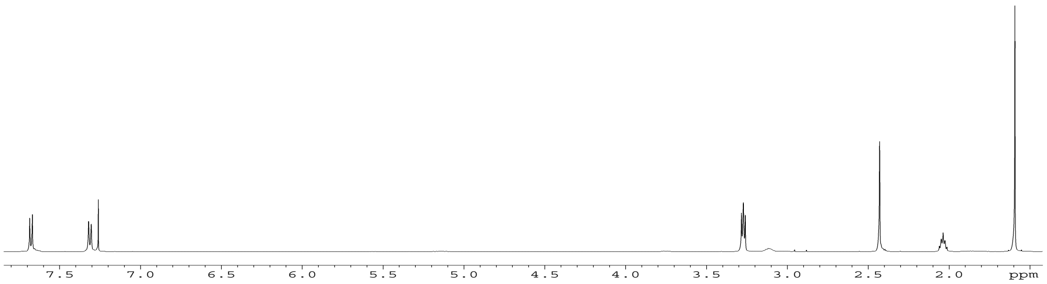

Figure S 3. ^{1}H NMR spectrum (500 MHz, $CDCl_3$ solution, 25°C) of *N,N′*-ditosyl-1,5-diazacyclooctane **1c**.

1.4 1,5-Diazacyclooctane dihydrobromide 1d[9]

In a 500 mL round-bottom flask, equipped with a reflux condenser and gas-lock *N*,*N′*-ditosyl-1,5-diazacyclooctane **1c** (6.62 g, 15.6 mmol) and phenol (4.4 g, 47 mmol) were dissolved in 33% hydrobromic acid in acetic acid. The solution was stirred at 80°C for 67 h and turned dark brown. The solvents were evaporated, removing the acetic acid by twice adding 30 mL ethanol to the initially obtained solid and evaporating to dryness. CH_2Cl_2 (100 mL) and water (100 mL) were added and the organic layer extracted two times with H_2O. The combined aqueous phases were evaporated, and the remaining yellow crystals redissolved in water (50 mL) and boiled up after addition of charcoal (Norite). Filtration and evaporation of the water yielded colourless crystals (3.84 g, 13.9 mmol, 89%) containing dark brown byproducts but was used as is in the following step.

^{1}H NMR (D_2O) δ: 3.13 (m, 8H, CH_2-N), 2.17 (m, 4H, CH_2). ^{13}C NMR (D_2O) δ: 43.8, 20.8.

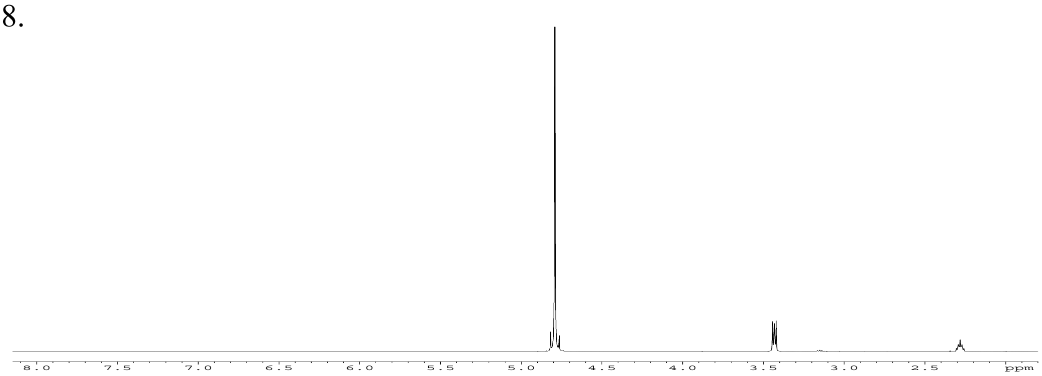

Figure S 4. ^{1}H NMR spectrum (500 MHz, $CDCl_3$ solution, 25°C) of 1,5-diazacyclo-octane dihydrobromide **1d**.

1.5 N,N'-Diphenyl-1,5-diazacyclooctan 1[10]

In a 50 mL round-bottom flask equipped with a reflux condenser and a gas lock $NaNH_2$ (9.8 g, 250 mmol) was added to a solution of tert-butanol (1.6 g, 22 mmol) in THF (dry, 20 ml) with vigorous stirring. 1,5-Diazacyclooctane dihydrobromide **1d** (3 g, 10.9 mmol) was dissolved in THF (dry, 10 mL) and added to the grey mixture resulting in heat and gas formation. The mixture was kept at 45 °C after the initial gas evolution had ceased. Bromobenzene (3.6 g, 23 mmol) was added in portions during 60 min, each addition being followed by gas evolution. The now brown suspension was stirred at 45 °C o.n. H_2O (30 mL) was added when the solution had cooled to r.t., causing violent gas evolution and heating. The mixture was poured into water (130 mL) and left for 2 h at r.t., when dark brown oil had formed on the surface of the yellow solution. The mixture was extracted with Et_2O×3 and solvents evaporated to give a brown solid. The crude product was dissolved in cyclohexane and the solution dried over Na_2SO_4 . Dark brown byproducts stuck to the drying agent and filtration produced a dark yellow solution from which the product precipitated from as a yellow solid upon storage in freezer (0.5 g 1.9 mmol, 17 %) m.p. 116°-117°C. ^{1}H NMR ($CDCl_3$) δ: 7.24 (m, 4H, *m*-Ph), 6.71 (m, 5H, *o*-Ph), 6.67 (m, 2H, *p*-Ph), 3.48 (m, 8H, CH_2-N), 2.02 (m, 4H, CH_2).
^{13}C NMR ($CDCl_3$) δ: 147.5, 129.3, 115.6, 111.3, 48.7, 25.2.

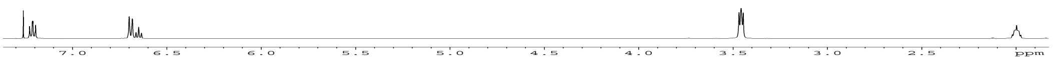

Figure S 5. ^{1}H NMR spectrum (500 MHz, $CDCl_3$ solution, 25°C) of *N*,*N′*-diphenyl-1,5-diazacyclooctane **1**.

2 1,8-Diaza-4,11-diazaniumyl-2,3,9,10(1,4)-tetrabenzenatricyclo[9.3.3.3^{4,8}]-eicosaphane bis(tetrafluoroborate) 5

N,*N′*-Diphenyl-1,5-diazacyclooctane **1** (195 mg, 0.73 mmol) in acetone (4 mL) was added to a solution of $AgBF_4$ (0.44 g, 1.15 mmol, 3.1 eq.) in acetone (4 mL). Directly upon mixing the combined solutions turned to an intense dark green colour and a silver mirror started to form on the inside of the vial within seconds. After standing for 48 h (r.t., dark) purification of the reaction mixture was possible by preparative HPLC achieving excellent separation and allowing isolation of the product (Fig. S8). Evaporation of solvents yielded compound **5** as a dark green solid (224 mg, 0.31 mmol, 87 %). It should be noted that **5** was stable at r.t. both as a solid as well as in solution for several months. mp: 208-210 °C; ^{1}H NMR (500 MHz, acetone-d_6, 25 °C) δ: 6.26 (broad, w = 367.3 Hz); ^{19}F NMR (470.34 MHz, acetone-d_6, 25 °C) δ: -147.0; (UV/Vis (acetone, r.t.) λ_{max} 325, 403, 760 nm; HRMS (m/z): $[M]^{2+}$ calcd. for $C_{36}H_{40}N_4$, 264.1621; found, 264.1593.

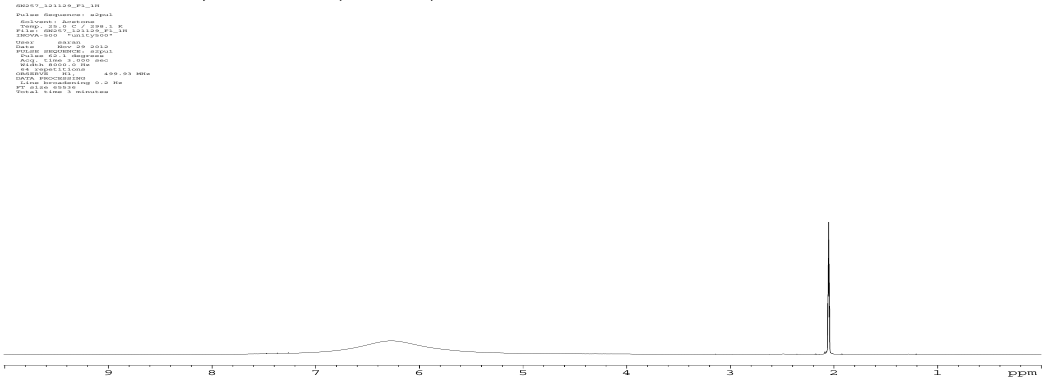

Figure S 6. ^{1}H NMR spectrum (500 MHz, acetone-d_6 solution, 25 °C) of compound **5**.

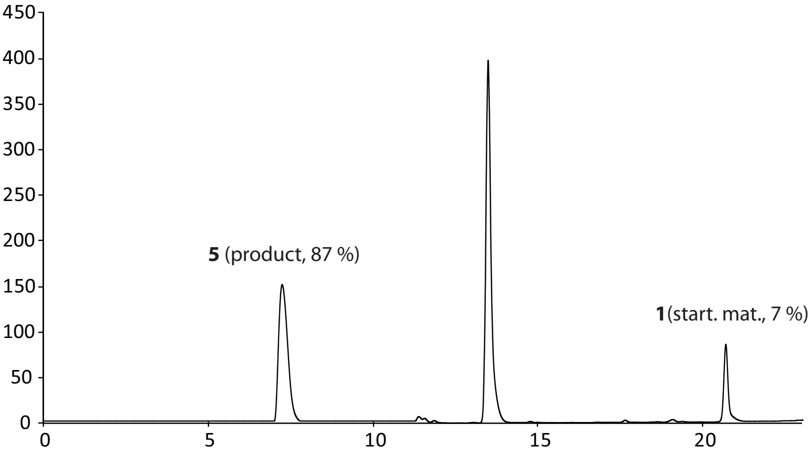

Figure S 7. HPLC chromatogram (254 nm) of a reaction mixture **1**+$AgBF_4$, after standing for 48 h. (50:50 MeCN:H_2O gradient).

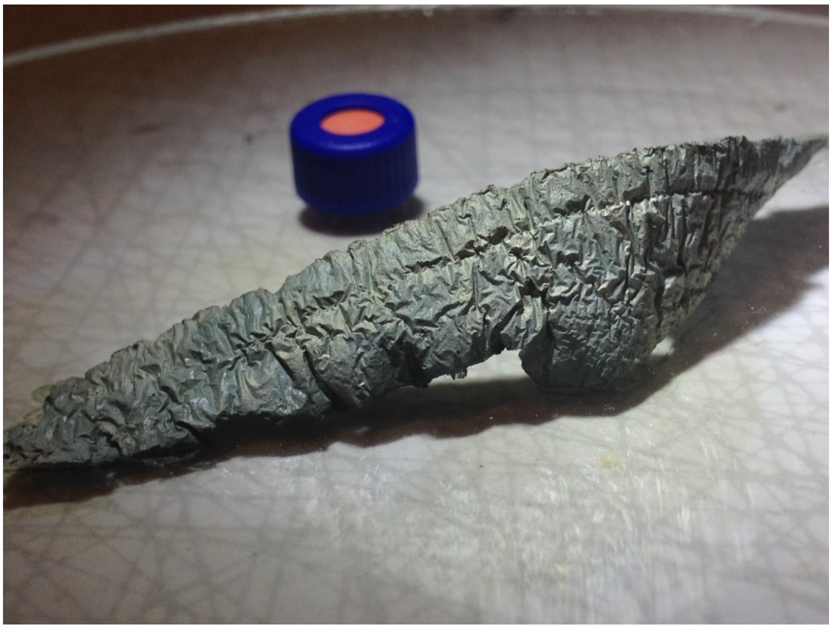

Figure S 8. Piece of silver foil formed during the preparation of **5**.

3 1,4,8,11-tetraaza-2,3,9,10(1,4)tetrabenzena-tricyclo[9.3.3.$3^{4,8}$]eicosaphane 8

Dication diradical **5** (2 mg, 0.0029 mmol) was dissolved in acetone (0.5 mL) and a saturated solution of Na_2SO_3 (0.5 mL, in H_2O) was added dropwise during stirring until the dark green solution turned pale brown. Extraction with $CHCl_3$ and evaporation of solvents afforded the bis(benzidino)phane **8** as an oily beige solid (2 mg, quant). ^{1}H NMR (500 MHz, $CDCl_3$/D_2O/acetone-d_6 1:1:1, 25 °C) δ: 6.62 (d, J = 8.6 Hz, 8H, Ar), 6.05 (d, J = 8.6 Hz, 8H, Ar), 3.78 (dm, J = 15.2 Hz, 8H, CH_2), 3.13-2.84 (m, 16 H, CH_2); UV/Vis (acetone, r.t.) λ_{max} 325 nm; HRMS (m/z): $[M+H]^+$ calcd. for $C_{36}H_{40}N_4$, 529.3315; found, 529.3321.

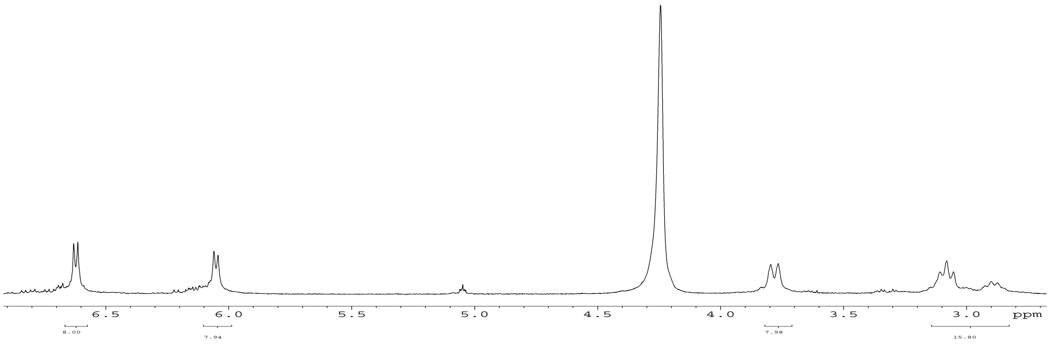

Figure S 9. ^{1}H NMR of 1,4,8,11-tetraaza-2,3,9,10(1,4)tetrabenzena-tricyclo-[9.3.3.$3^{4,8}$]eicosaphane **9** (500 MHz, acetone-d_6 solution, 25 °C).

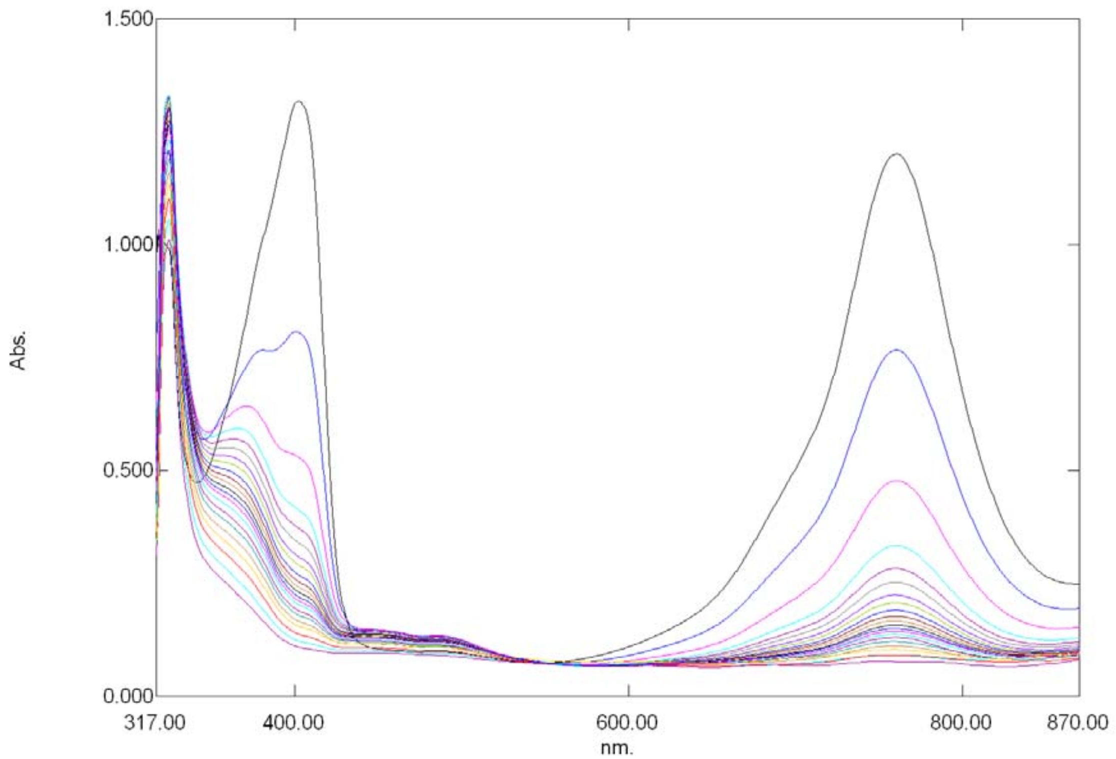

Figure S 10. UV/Vis titration of compound **5** (top line, black) and hydroquinone (acetone solution, r.t.) showing reduction of the radical species.

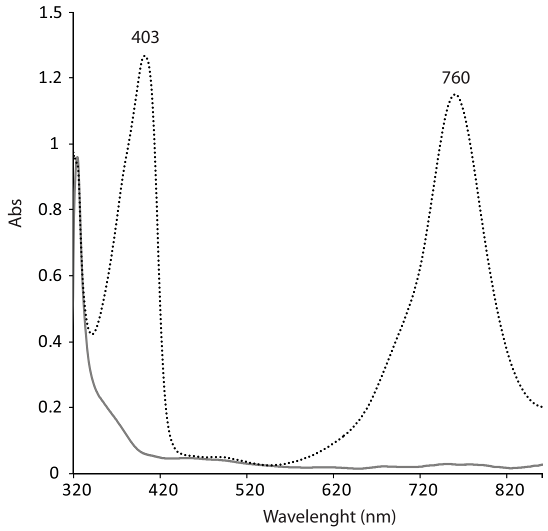

Figure S 11. UV/Vis spectra of dication diradical **5** (¨) and cyclophane **8** (—).

4 1,8-Diaza-4,11-diazaniumyl-2,3,9,10(1,4)tetrabenzena-tricyclo-[9.3.3.3^{4,8}.1^{6,19}.1^{13,16}]docosaphane bis(tetrafluoroborate) 6

3,7-Diphenyl-3,7-diazabicyclo[3.3.1]nonane **2** (50 mg, 0.18 mmol) in acetone (1 mL) was added to a solution of $AgBF_4$ (105 mg, 0.54 mmol, 3.1 eq.) in acetone (1 mL). Directly upon mixing the combined solutions turned into an intense dark green colour and a silver mirror started to form on the inside of the vial within minutes. After standing for 48 h (r.t, dark) purification of the reaction mixture with preparative HPLC afforded isolation of the product **6** that after evaporation of solvents was obtained as a dark green solid (48 mg, 0.07 mmol, 73 %). mp: 120 °C (dec.); ^{1}H NMR (500 MHz, acetone-d$_6$, 25 °C) δ: 3.11 (broad, w = 182.1 Hz); ^{19}F NMR (470.34 MHz, acetone-d$_6$, 25 °C) δ: -147.1; UV/Vis (acetone, r.t.) λ_{max} 223, 323, 394, 751 nm; HRMS (m/z): $[M+BF_4]^+$ calcd. for $C_{38}H_{40}BF_4N_4$, 639.3271; found, 639.3264.

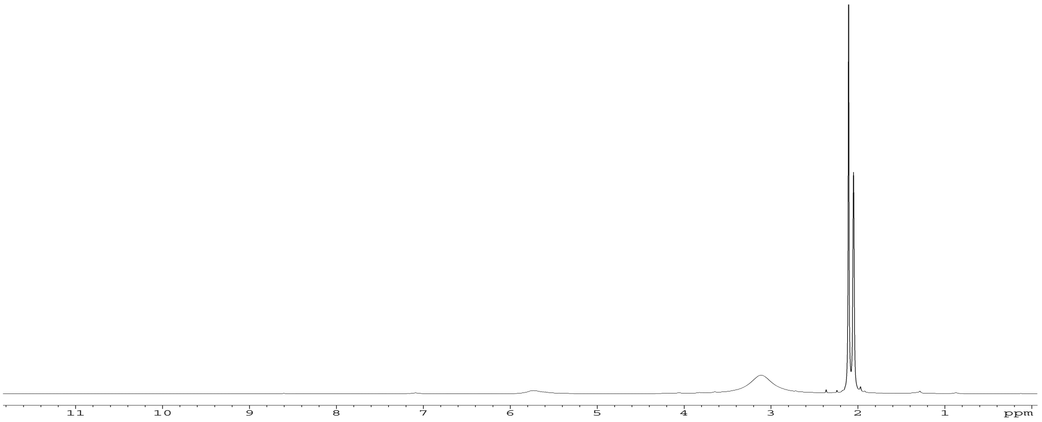

Figure S 12. ^{1}H NMR spectrum of compound **6** (500 MHz, acetone-d$_6$ solution, 25 °C).

5 6,13,16,19-Tetracarbomethoxy-1,8-Diaza-4,11-diazaniumyl-2,3,9,10(1,4)-tetrabenzena-tricyclo[9.3.3.3^{4,8}.1^{6,19}.1^{13,16}]docosaphane-21,22-dione bis(tetrafluoroborate) 7

3,7-Diphenyl-1,5-dicarbomethoxybispidinone **3** (50 mg, 0.12 mmol) in acetone (1 mL) was added to a solution of $AgBF_4$ (71.5 mg, 0.37 mmol, 3.1 eq.) in acetone (1 mL). Directly upon mixing the combined solutions turned to a dark brown that within hours shifted into deep green. After standing (r.t., dark) for 48 h, a silver flake had formed on the inside of the vial. The flake could be removed from the flask as a single piece of Ag(s) (see Supplementary for image). HPLC analysis of the reaction mixture proved a complex mixture and the product **7** could not be fully isolated. ^{1}H NMR (500 MHz, acetone-d$_6$, 25 °C, crude mixture) δ: 4.70 (broad, w = 115.5 Hz); HRMS (m/z): $[M+3H]^+$ calcd. for $C_{46}H_{47}N_4O_{10}$, 815.3292; found, 815.3279; analysis (calcd., found for Ag-flake): Ag (100.0, 100.0); UV/Vis (acetone, r.t., crude) λ_{max} 332, 389, 746 nm.

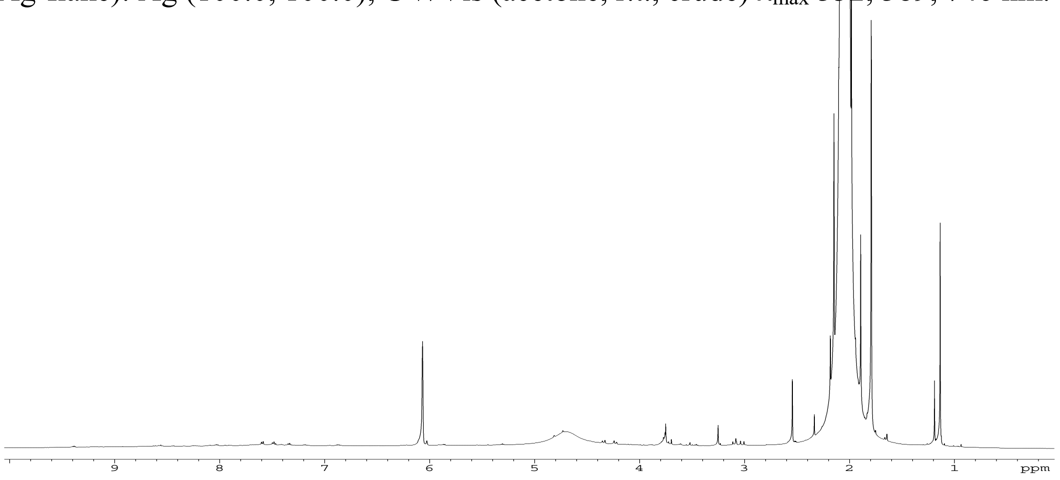

Figure S 13. ^{1}H NMR spectrum of the crude reaction mixture of compound **7** (500 MHz, acetone-d$_6$ solution, 25 °C).

6 Job plots for formation of dication diradicals 5, 6 and 7

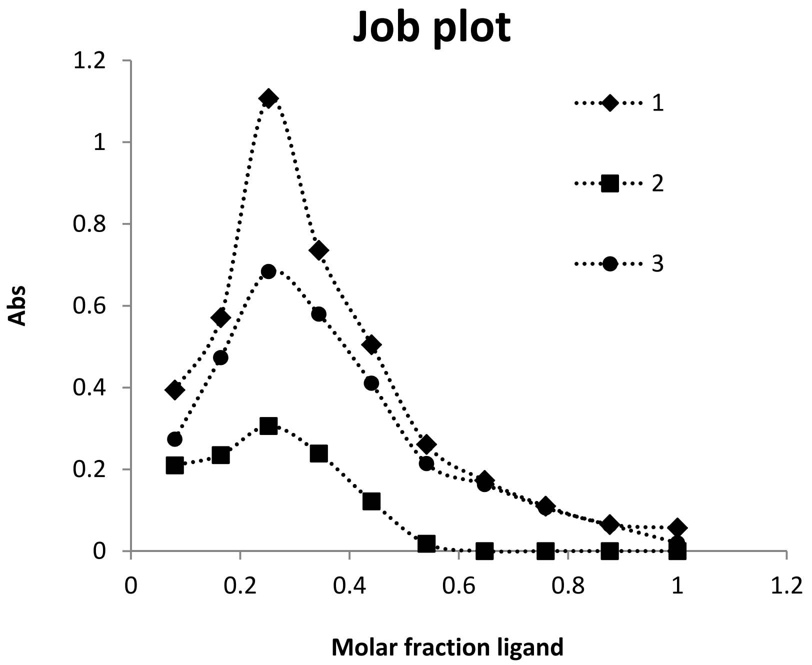

Figure S 14. Job's plot for the mixture of ligands **1** – **3** and $AgBF_4$ solutions showing maximum absorbance for 1:3 ratio of ligand:$AgBF_4$. UV/Vis (acetone, r.t.) absorbance at 760 (compound **1**), 751 (compound **2**) or 746 (compound **3**) nm was monitored.

7 Diffusion coefficients for the discussed species

Table S1. Diffusion coefficients (D) and hydrodynamic radius (r_H) of free ligands or BF_4^- in reaction mixtures of compounds **1 - 4**. D referenced to HDO in H_2O (D = 1.9×10^{-9} m^2 s^{-1}). LED-PGSE experiments[7] were performed monitoring ^1H or ^{19}F.

**Species**	**D (m^2 s^{-1})**	**r_H (Å)[b]**
$AgBF_4$[a]	9.5×10^{-10}	4.2
N,*N′*-Diphenyl-1,5-diazacyclooctane **1**	8.6×10^{-10}	4.7
3,7-Diphenyl-3,7-diazabicyclo[3.3.1]nonane **2**	7.6×10^{-10}	5.3
3,7-Diphenyl-1,5-dicarbomethoxybispidinone **3**	7.6×10^{-10}	5.3
N,*N¢*-Diphenylpiperazine **4**	8.6×10^{-10}	4.7
dication diradical **5**[a]	1.9×10^{-10}	21.2
dication diradical **6**[a]	1.9×10^{-10}	21.2
dication diradical **7**[a]	1.9×10^{-10}	21.2
Piperazine **4** + $AgBF_4$[a]	9.5×10^{-10}	4.2

[a] D measured for ^{19}F. [b] Calculated from D using the Stokes-Einstein equation.

The highest diffusion coefficients were obtained for the tetrafluoroborate ions (MW= 86.80), followed by the diamines **1** – **4** (MW = 266.4, 278.4, 408.5, 238.3). For the reaction products **5** – **7** (MW of dications = 528.7, 552.8, 812.9), considerably lower diffusion coefficients, corresponding to larger hydrodynamic radii, were obtained by monitoring the ^{19}F NMR signals of the BF_4^- counterions.

8 EPR and magnetic susceptibility data

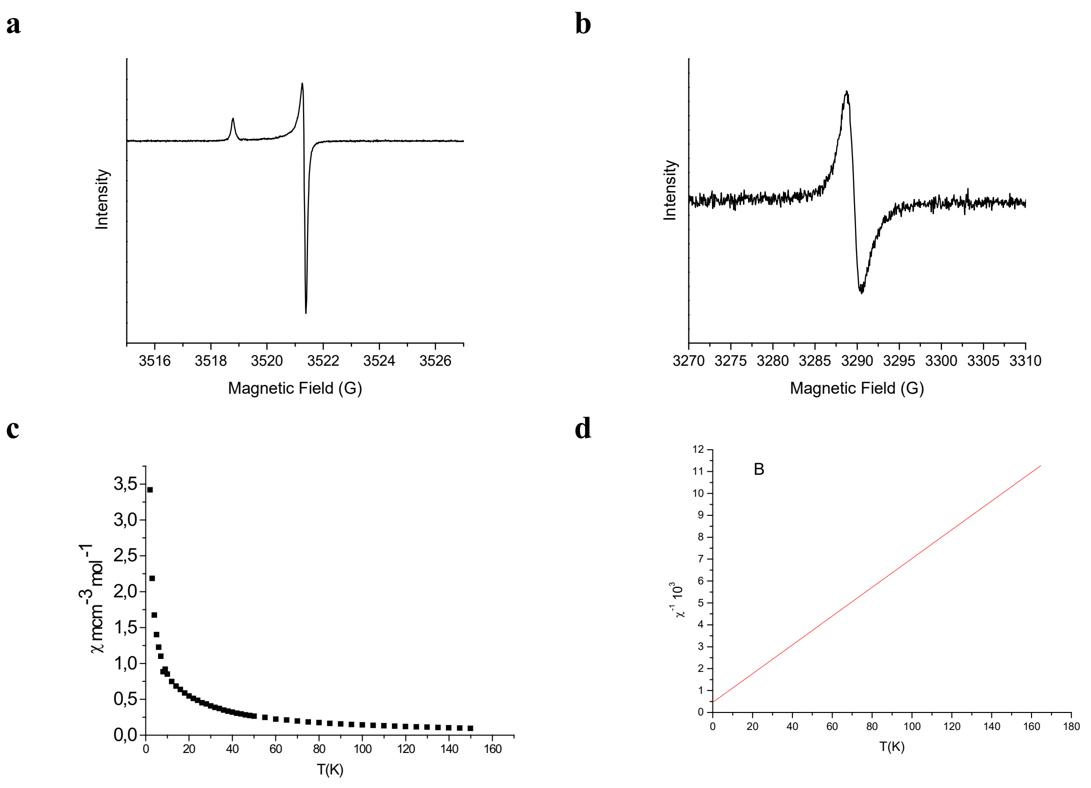

Figure S 15. EPR spectra and susceptibility data of **5** indicating both delocalization of unpaired electrons and effect of π-stacking. a) Solid sample measured at room temperature, $g_{||} = 2.0040$ and $g_{\perp} = 2.0025$; b) frozen solution (7 K) in dimethylsulfoxide, g = 2.004; c) temperature dependence of molar susceptibility; d) Curie-Weiss plot. EPR parameters: microwave frequency/microwave power, a) 9.8696 GHz/20 µW, b) 9.2804 GHz/2 µW.

9 X-ray crystallography data

9.1 Crystal from acetone-water solution after purification by HPLC

Table S 1: Crystal data and structure refinement details.

Identification code	**2013acc0007** (SN130118A)	
Empirical formula	$C_{37.50}H_{43}B_2F_8N_4O_{0.50}$	
	$C_{36}H_{40}N_4$, BF_4, $0.5(C_3H_6O)$	
Formula weight	731.38	
Temperature	100(2) K	
Wavelength	0.71073 Å	
Crystal system	Triclinic	
Space group	*P*–1	
Unit cell dimensions	a = 16.352(2) Å	α = 86.102(10)°
	b = 20.553(3) Å	β = 81.746(8)°
	c = 20.842(3) Å	γ = 80.154(7)°
Volume	6823.5(17) Å^3	
Z	8	
Density (calculated)	1.424 Mg / m^3	
Absorption coefficient	0.115 mm^{-1}	
F(000)	3056	
Crystal	Plate; Green	
Crystal size	0.220 × 0.100 × 0.010 mm^3	
θ range for data collection	2.959 – 25.350°	
Index ranges	$-15 \le h \le 19$, $-24 \le k \le 24$, $-24 \le l \le 25$	
Reflections collected	79085	
Independent reflections	24930 [R_{int} = 0.0659]	
Completeness to θ = 25.242°	99.8 %	
Absorption correction	Semi–empirical from equivalents	
Max. and min. transmission	1.000 and 0.845	
Refinement method	Full-matrix least-squares on F^2	
Data / restraints / parameters	24930 / 1771 / 2099	
Goodness-of-fit on F^2	1.096	
Final R indices [$F^2 > 2\sigma(F^2)$]	$R1$ = 0.1285, $wR2$ = 0.3244	
R indices (all data)	$R1$ = 0.1703, $wR2$ = 0.3559	
Largest diff. peak and hole	1.629 and –0.659 e Å^{-3}	

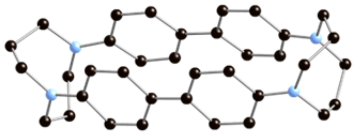

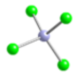

Diffractometer: *Rigaku AFC12* goniometer equipped with an enhanced sensitivity (HG) *Saturn724+* detector mounted at the window of an *FR-E+ SuperBright* molybdenum rotating anode generator with HF *Varimax* optics (100µm focus). **Cell determination, Data collection, Data reduction and cell refinement & Absorption correction**: CrystalClear-SM Expert 2.0 r7 (Rigaku, 2011) , **Structure solution**: SHELXS97 (Sheldrick, G.M. (2008). *Acta Cryst.* **A64**, 112-122). **Structure refinement**: SHELXL2012 (G. M. Sheldrick (2012), University of Göttingen, Germany). **Graphics:** CrystalMaker: a crystal and molecular structures program for Mac and Windows. CrystalMaker Software Ltd, Oxford, England (www.crystalmaker.com)

Special details: All hydrogen atoms were placed in idealised positions and refined using a riding model.
The cations arrange themselves along the a-axis with the anions and solvent molecules arranged in channels between them. The BF4 in particular are disordered along these channels and it is possible to refine many fractionally occupied position for these. The result is a refinement that still contains large residual peaks associated with these disordered regions.

Table S 2: Atomic coordinates [× 10^4], equivalent isotropic displacement parameters [Å^2 × 10^3] and site occupancy factors. U_{eq} is defined as one third of the trace of the orthogonalized U^{ij} tensor.

Atom	*x*	*y*	*z*	U_{eq}	*S.o.f.*
N1	−2623(2)	9781(2)	2271(2)	33(1)	1
N2	−3140(2)	9643(2)	3582(2)	28(1)	1
N3	2511(2)	10212(2)	4335(2)	30(1)	1
N4	3083(2)	10217(2)	3039(2)	30(1)	1
C1	−3331(3)	10341(3)	2276(2)	37(1)	1
C2	−4049(4)	10252(4)	2799(3)	59(2)	1
C3	−3834(3)	10202(2)	3484(2)	34(1)	1
C4	−3298(3)	8983(2)	3457(2)	35(1)	1
C5	−2761(3)	8688(3)	2848(2)	40(1)	1
C6	−2833(3)	9115(3)	2227(2)	39(1)	1
C7	−2419(2)	9733(2)	3796(2)	24(1)	1
C8	−2225(3)	10380(2)	3871(2)	30(1)	1
C9	−1461(3)	10452(2)	4009(2)	27(1)	1
C10	−814(2)	9909(2)	4085(2)	22(1)	1
C11	−1033(3)	9273(2)	4062(2)	26(1)	1
C12	−1801(3)	9184(2)	3922(2)	27(1)	1
C13	20(3)	9993(2)	4181(2)	22(1)	1
C14	254(3)	10629(2)	4187(2)	26(1)	1
C15	1048(3)	10716(2)	4252(2)	26(1)	1
C16	1701(3)	10161(2)	4317(2)	27(1)	1
C17	1448(3)	9519(2)	4350(2)	27(1)	1
C18	653(3)	9453(2)	4285(2)	27(1)	1
C19	3151(3)	9623(2)	4402(2)	33(1)	1
C20	3405(3)	9225(3)	3790(2)	36(1)	1
C21	3687(3)	9625(3)	3167(2)	36(1)	1
C22	3372(3)	10850(2)	3121(2)	33(1)	1
C23	3552(3)	10918(3)	3812(2)	38(1)	1
C24	2805(3)	10857(2)	4332(2)	35(1)	1
C25	2351(3)	10184(2)	2819(2)	28(1)	1
C26	2144(3)	9570(2)	2674(2)	30(1)	1
C27	1372(3)	9519(2)	2522(2)	30(1)	1
C28	723(3)	10074(2)	2499(2)	28(1)	1
C29	949(3)	10689(2)	2604(2)	30(1)	1
C30	1720(3)	10753(2)	2754(2)	29(1)	1
C31	−120(3)	10010(2)	2393(2)	28(1)	1
C32	−339(3)	9388(2)	2284(2)	32(1)	1
C33	−1144(3)	9317(2)	2223(2)	33(1)	1
C34	−1811(3)	9866(2)	2281(2)	30(1)	1
C35	−1587(3)	10496(2)	2340(2)	32(1)	1
C36	−785(3)	10559(2)	2405(2)	30(1)	1
N5	7370(2)	4774(2)	692(2)	25(1)	1

N6	6882(2)	4793(2)	1991(2)	24(1)	1
N7	12630(3)	5204(3)	2640(2)	52(1)	1
N8	13136(2)	5194(2)	1325(2)	43(1)	1
C37	6689(3)	5330(2)	623(2)	29(1)	1
C38	5971(3)	5352(2)	1181(2)	31(1)	1
C39	6247(3)	5366(2)	1846(2)	28(1)	1
C40	6650(3)	4136(2)	1950(2)	32(1)	1
C41	7134(3)	3747(2)	1378(2)	33(1)	1
C42	7125(3)	4112(2)	713(2)	34(1)	1
C43	7608(3)	4861(2)	2212(2)	24(1)	1
C44	7835(3)	5501(2)	2280(2)	27(1)	1
C45	8609(3)	5551(2)	2424(2)	29(1)	1
C46	9231(3)	4986(2)	2522(2)	26(1)	1
C47	8965(3)	4365(2)	2518(2)	32(1)	1
C48	8195(3)	4295(2)	2376(2)	28(1)	1
C49	10084(3)	5049(2)	2599(2)	31(1)	1
C50	10355(3)	5672(3)	2592(2)	34(1)	1
C51	11166(3)	5737(3)	2622(2)	41(1)	1
C52	11794(3)	5170(3)	2664(2)	40(1)	1
C53	11523(3)	4541(3)	2713(2)	45(1)	1
C54	10710(3)	4487(3)	2685(2)	37(1)	1
C55	13270(4)	4604(4)	2688(3)	72(2)	1
C56	13463(4)	4168(4)	2070(4)	79(2)	1
C57	13746(3)	4587(4)	1461(3)	65(2)	1
C58	13415(3)	5828(3)	1379(3)	55(2)	1
C59	13668(4)	5852(4)	2066(3)	77(2)	1
C60	12936(3)	5852(4)	2605(3)	66(2)	1
C61	12381(3)	5142(2)	1143(2)	30(1)	1
C62	12163(3)	4520(3)	1038(2)	35(1)	1
C63	11380(3)	4463(2)	922(2)	31(1)	1
C64	10726(2)	5021(2)	896(2)	21(1)	1
C65	10976(3)	5643(2)	943(2)	30(1)	1
C66	11762(3)	5706(2)	1063(2)	34(1)	1
C67	9885(2)	4960(2)	818(2)	20(1)	1
C68	9636(3)	4340(2)	751(2)	25(1)	1
C69	8832(3)	4282(2)	697(2)	27(1)	1
C70	8182(2)	4839(2)	708(2)	21(1)	1
C71	8424(3)	5469(2)	752(2)	28(1)	1
C72	9236(3)	5522(2)	809(2)	27(1)	1
N9	−3123(2)	3652(2)	4899(2)	35(1)	1
N10	−2738(2)	2348(2)	5282(2)	35(1)	1
N11	3057(2)	3015(2)	5572(2)	37(1)	1
N12	2621(2)	4338(2)	5281(2)	30(1)	1
C73	−3772(3)	3713(3)	5471(3)	44(1)	1
C74	−3590(3)	3195(3)	6031(3)	45(1)	1
C75	−3404(3)	2472(3)	5838(3)	42(1)	1
C76	−3029(3)	2199(3)	4677(3)	42(1)	1
C77	−3674(3)	2749(3)	4438(3)	44(1)	1
C78	−3376(3)	3412(3)	4317(3)	42(1)	1

C79	−1917(3)	2337(2)	5343(2)	31(1)	1
C80	−1643(3)	2384(2)	5957(2)	34(1)	1
C81	−830(3)	2425(2)	6008(2)	33(1)	1
C82	−197(3)	2433(2)	5456(2)	27(1)	1
C83	−462(3)	2352(2)	4860(2)	30(1)	1
C84	−1273(3)	2305(2)	4796(2)	32(1)	1
C85	654(3)	2516(2)	5511(2)	30(1)	1
C86	926(3)	2620(2)	6109(2)	31(1)	1
C87	1715(3)	2761(2)	6143(2)	31(1)	1
C88	2311(3)	2810(2)	5571(2)	34(1)	1
C89	2068(3)	2633(2)	4984(2)	34(1)	1
C90	1273(3)	2513(2)	4957(2)	33(1)	1
C91	3328(3)	3219(3)	6169(3)	45(1)	1
C92	3597(3)	3898(3)	6097(3)	51(2)	1
C93	2912(3)	4454(3)	5895(2)	36(1)	1
C94	3294(3)	4261(2)	4720(2)	35(1)	1
C95	3490(3)	3570(3)	4474(3)	42(1)	1
C96	3687(3)	3012(3)	4987(3)	42(1)	1
C97	1822(3)	4316(2)	5215(2)	25(1)	1
C98	1561(3)	4254(2)	4597(2)	27(1)	1
C99	770(3)	4188(2)	4524(2)	23(1)	1
C100	132(3)	4179(2)	5065(2)	22(1)	1
C101	377(3)	4259(2)	5676(2)	29(1)	1
C102	1179(3)	4322(2)	5757(2)	27(1)	1
C103	−703(3)	4090(2)	5000(2)	24(1)	1
C104	−955(3)	3959(2)	4398(2)	28(1)	1
C105	−1741(3)	3837(2)	4352(2)	32(1)	1
C106	−2364(3)	3841(2)	4908(2)	29(1)	1
C107	−2152(3)	4045(2)	5491(2)	31(1)	1
C108	−1358(3)	4150(2)	5539(2)	30(1)	1
N13	6851(2)	1910(2)	10101(2)	22(1)	1
N14	7417(2)	630(2)	9822(2)	22(1)	1
N15	13093(2)	1279(2)	10492(2)	24(1)	1
N16	12534(2)	2611(2)	10616(2)	22(1)	1
C109	6265(3)	1651(2)	10628(2)	27(1)	1
C110	6019(3)	999(2)	10470(2)	28(1)	1
C111	6767(2)	441(2)	10339(2)	25(1)	1
C112	6559(3)	1997(2)	9451(2)	28(1)	1
C113	7059(3)	1514(2)	8959(2)	28(1)	1
C114	7127(2)	775(2)	9180(2)	24(1)	1
C115	8215(2)	652(2)	9915(2)	21(1)	1
C116	8499(2)	556(2)	10538(2)	21(1)	1
C117	9291(2)	636(2)	10618(2)	20(1)	1
C118	9885(2)	806(2)	10099(2)	20(1)	1
C119	9610(2)	876(2)	9475(2)	22(1)	1
C120	8828(2)	795(2)	9385(2)	23(1)	1
C121	10713(2)	900(2)	10192(2)	21(1)	1
C122	10993(2)	828(2)	10807(2)	22(1)	1
C123	11776(2)	924(2)	10907(2)	22(1)	1

C124	12349(2)	1126(2)	10385(2)	22(1)	1
C125	12089(3)	1182(2)	9754(2)	24(1)	1
C126	11306(2)	1075(2)	9671(2)	22(1)	1
C127	13334(3)	1264(2)	11150(2)	27(1)	1
C128	12855(3)	1834(2)	11565(2)	25(1)	1
C129	12841(3)	2528(2)	11253(2)	25(1)	1
C130	13186(3)	2688(2)	10051(2)	27(1)	1
C131	13941(3)	2138(2)	10028(2)	30(1)	1
C132	13740(3)	1450(2)	9965(2)	32(1)	1
C133	11710(2)	2647(2)	10559(2)	21(1)	1
C134	11400(3)	2709(2)	9951(2)	26(1)	1
C135	10583(3)	2670(2)	9898(2)	25(1)	1
C136	9985(2)	2563(2)	10448(2)	19(1)	1
C137	10280(2)	2554(2)	11059(2)	22(1)	1
C138	11096(3)	2602(2)	11118(2)	23(1)	1
C139	9156(2)	2465(2)	10377(2)	23(1)	1
C140	8834(3)	2554(2)	9779(2)	24(1)	1
C141	8077(3)	2407(2)	9690(2)	24(1)	1
C142	7551(2)	2137(2)	10210(2)	22(1)	1
C143	7826(2)	2090(2)	10829(2)	24(1)	1
C144	8594(3)	2245(2)	10912(2)	27(1)	1
B1	5184(4)	3544(3)	188(3)	49(1)	1
F1	5148(2)	4227(1)	174(2)	52(1)	1
F2	5063(2)	3338(2)	–412(2)	60(1)	1
F3	4559(3)	3353(2)	647(2)	96(1)	1
F4	5946(2)	3235(2)	328(2)	84(1)	1
B2	5197(3)	4775(3)	3565(3)	47(1)	1
F5	6045(2)	4765(2)	3338(1)	44(1)	1
F6	4730(2)	5344(2)	3317(2)	62(1)	1
F7	4956(2)	4217(2)	3364(2)	76(1)	1
F8	5102(2)	4774(2)	4233(2)	79(1)	1
B3A	7983(6)	2312(4)	2811(4)	113(1)	0.765(4)
F9A	8774(5)	2415(3)	2448(3)	128(2)	0.765(4)
F10A	7470(5)	2903(2)	2985(3)	122(2)	0.765(4)
F11A	7608(5)	2040(3)	2380(2)	112(1)	0.765(4)
F12A	8182(5)	1918(3)	3331(2)	108(2)	0.765(4)
B3B	8298(8)	2324(6)	2842(6)	115(1)	0.235(4)
F9B	9071(9)	2069(8)	2478(7)	115(2)	0.235(4)
F10B	8368(13)	2842(6)	3215(6)	118(2)	0.235(4)
F11B	7741(10)	2600(8)	2413(6)	120(2)	0.235(4)
F12B	8007(13)	1824(6)	3226(7)	114(2)	0.235(4)
B4	1656(5)	2755(4)	3225(3)	77(1)	1
F13	2136(3)	3164(2)	3441(2)	93(1)	1
F14	1670(3)	2798(2)	2582(2)	89(1)	1
F15	832(3)	3028(2)	3505(2)	95(1)	1
F16	1871(3)	2117(2)	3460(2)	83(1)	1
B5	4815(3)	1325(3)	5199(3)	47(1)	1
F17	5413(2)	1328(2)	4643(2)	73(1)	1
F18	4015(2)	1450(2)	5035(2)	55(1)	1
F19	4940(2)	712(2)	5530(2)	68(1)	1

F20	4931(2)	1802(2)	5595(1)	48(1)	1
B6A	2922(7)	2750(5)	8156(5)	61(1)	0.306(4)
F21A	3622(6)	2927(5)	7740(5)	67(2)	0.306(4)
F22A	2270(6)	2790(6)	7783(5)	70(2)	0.306(4)
F23A	2647(8)	3200(5)	8641(5)	65(2)	0.306(4)
F24A	3173(8)	2128(4)	8416(5)	58(2)	0.306(4)
B6B	2614(5)	2810(4)	8278(4)	58(1)	0.694(4)
F21B	2860(4)	2890(2)	7609(2)	63(1)	0.694(4)
F22B	1748(3)	3015(3)	8400(3)	73(1)	0.694(4)
F23B	2977(3)	3240(2)	8593(2)	55(1)	0.694(4)
F24B	2852(4)	2172(2)	8499(2)	58(1)	0.694(4)
B7A	5126(6)	327(5)	8655(4)	38(1)	0.436(4)
F25A	5528(4)	865(3)	8430(3)	50(1)	0.436(4)
F26A	4266(4)	528(4)	8764(4)	41(2)	0.436(4)
F27A	5428(4)	68(4)	9226(3)	38(2)	0.436(4)
F28A	5321(4)	−151(3)	8190(3)	43(1)	0.436(4)
B7B	5105(5)	437(4)	8719(4)	36(1)	0.564(4)
F25B	5212(3)	1093(2)	8782(3)	39(1)	0.564(4)
F26B	4274(3)	407(3)	8679(3)	40(1)	0.564(4)
F27B	5326(3)	80(3)	9279(2)	36(1)	0.564(4)
F28B	5629(4)	172(3)	8192(3)	62(1)	0.564(4)
B8A	9147(8)	2438(6)	7836(6)	74(1)	0.1896(12)
F29A	8528(8)	2691(8)	7431(6)	77(2)	0.1896(12)
F30A	9297(12)	2924(6)	8221(7)	72(2)	0.1896(12)
F31A	8921(12)	1910(7)	8241(7)	74(2)	0.1896(12)
F32A	9897(8)	2185(8)	7469(7)	74(2)	0.1896(12)
B8B	8767(7)	2433(5)	7880(5)	73(1)	0.4726(12)
F29B	8396(6)	2119(4)	7446(3)	83(1)	0.4726(12)
F30B	8161(5)	2889(3)	8202(3)	73(2)	0.4726(12)
F31B	9037(6)	1906(4)	8301(3)	70(2)	0.4726(12)
F32B	9415(5)	2707(4)	7516(3)	73(1)	0.4726(12)
B8C	9518(7)	2348(5)	8067(5)	72(1)	0.3372(12)
F29C	10147(6)	2049(5)	8445(4)	71(2)	0.3372(12)
F31C	8898(6)	1976(5)	7976(5)	77(1)	0.3372(12)
F30C	9131(8)	2948(4)	8325(5)	73(2)	0.3372(12)
F32C	9878(8)	2505(5)	7448(4)	79(2)	0.3372(12)
O1A	4663(7)	1926(5)	2150(6)	85(1)	0.450(8)
C21A	5066(11)	2133(8)	3184(6)	85(1)	0.450(8)
C22A	5051(8)	2239(6)	2473(6)	85(1)	0.450(8)
C23A	5618(9)	2696(7)	2213(8)	85(1)	0.450(8)
O1B	4910(6)	1755(4)	2133(5)	85(1)	0.550(8)
C21B	4416(9)	2479(7)	3060(6)	85(1)	0.550(8)
C22B	4888(7)	2295(5)	2421(5)	85(1)	0.550(8)
C23B	5383(8)	2770(6)	2095(7)	85(1)	0.550(8)
O2	6209(4)	2015(4)	7400(3)	120(2)	1
C301	5134(5)	2548(5)	8174(3)	108(3)	1
C302	5421(4)	2140(4)	7623(3)	72(2)	1
C303	4802(4)	1863(4)	7333(4)	89(3)	1

Table S 3: Bond lengths [Å] and angles [°].

N1–C34	1.372(6)
N1–C6	1.480(7)
N1–C1	1.485(6)
N2–C7	1.364(5)
N2–C4	1.471(6)
N2–C3	1.496(6)
N3–C16	1.353(6)
N3–C19	1.472(6)
N3–C24	1.485(6)
N4–C25	1.357(6)
N4–C21	1.464(6)
N4–C22	1.486(6)
C1–C2	1.509(8)
C2–C3	1.512(8)
C4–C5	1.533(7)
C5–C6	1.519(7)
C7–C12	1.416(6)
C7–C8	1.442(6)
C8–C9	1.356(6)
C9–C10	1.418(6)
C10–C11	1.419(6)
C10–C13	1.447(6)
C11–C12	1.374(6)
C13–C18	1.411(6)
C13–C14	1.425(6)
C14–C15	1.365(6)
C15–C16	1.437(6)
C16–C17	1.442(6)
C17–C18	1.357(6)
C19–C20	1.530(7)
C20–C21	1.544(7)
C22–C23	1.531(7)
C23–C24	1.528(6)
C25–C26	1.422(7)
C25–C30	1.434(6)
C26–C27	1.367(6)
C27–C28	1.421(6)
C28–C29	1.416(7)
C28–C31	1.457(6)
C29–C30	1.371(6)
C31–C32	1.424(7)
C31–C36	1.426(6)
C32–C33	1.373(6)
C33–C34	1.427(6)
C34–C35	1.422(7)
C35–C36	1.366(6)
N5–C70	1.363(5)
N5–C37	1.468(6)
N5–C42	1.479(6)
N6–C43	1.365(6)
N6–C39	1.476(5)
N6–C40	1.475(6)
N7–C52	1.374(6)
N7–C55	1.482(8)
N7–C60	1.495(8)
N8–C61	1.366(6)
N8–C58	1.469(7)
N8–C57	1.497(8)
C37–C38	1.528(6)
C38–C39	1.523(6)
C40–C41	1.526(6)
C41–C42	1.531(7)
C43–C48	1.432(6)
C43–C44	1.447(6)
C44–C45	1.365(6)
C45–C46	1.432(6)
C46–C47	1.417(7)
C46–C49	1.455(6)
C47–C48	1.366(6)
C49–C50	1.423(7)
C49–C54	1.426(7)
C50–C51	1.367(7)
C51–C52	1.423(7)
C52–C53	1.430(8)
C53–C54	1.363(7)
C55–C56	1.582(10)
C56–C57	1.545(10)
C58–C59	1.554(8)
C59–C60	1.519(8)
C61–C66	1.419(7)
C61–C62	1.424(7)
C62–C63	1.361(6)
C63–C64	1.433(6)
C64–C65	1.420(6)
C64–C67	1.434(6)
C65–C66	1.373(6)
C67–C68	1.425(6)
C67–C72	1.431(6)
C68–C69	1.360(6)
C69–C70	1.422(6)
C70–C71	1.428(6)
C71–C72	1.373(6)

N9–C106	1.365(6)	N13–C109	1.484(5)
N9–C73	1.475(6)	N13–C112	1.490(5)
N9–C78	1.477(7)	N14–C115	1.355(5)
N10–C79	1.364(6)	N14–C114	1.477(5)
N10–C75	1.475(6)	N14–C111	1.483(5)
N10–C76	1.478(6)	N15–C124	1.359(5)
N11–C88	1.358(6)	N15–C127	1.477(5)
N11–C96	1.478(6)	N15–C132	1.481(5)
N11–C91	1.487(7)	N16–C133	1.357(5)
N12–C97	1.342(6)	N16–C129	1.475(5)
N12–C93	1.475(6)	N16–C130	1.490(5)
N12–C94	1.483(6)	C109–C110	1.532(6)
C73–C74	1.551(8)	C110–C111	1.534(6)
C74–C75	1.534(7)	C112–C113	1.523(6)
C76–C77	1.518(7)	C113–C114	1.549(6)
C77–C78	1.519(7)	C115–C120	1.432(6)
C79–C80	1.427(7)	C115–C116	1.429(6)
C79–C84	1.433(6)	C116–C117	1.369(6)
C80–C81	1.366(7)	C117–C118	1.413(5)
C81–C82	1.435(6)	C118–C119	1.426(6)
C82–C83	1.401(6)	C118–C121	1.442(5)
C82–C85	1.451(6)	C119–C120	1.358(6)
C83–C84	1.373(6)	C121–C122	1.412(6)
C85–C86	1.423(7)	C121–C126	1.420(6)
C85–C90	1.422(6)	C122–C123	1.375(6)
C86–C87	1.382(7)	C123–C124	1.418(6)
C87–C88	1.437(7)	C124–C125	1.432(6)
C88–C89	1.427(7)	C125–C126	1.372(6)
C89–C90	1.374(7)	C127–C128	1.538(6)
C91–C92	1.525(8)	C128–C129	1.525(6)
C92–C93	1.541(7)	C130–C131	1.524(6)
C94–C95	1.510(7)	C131–C132	1.524(7)
C95–C96	1.539(7)	C133–C134	1.422(6)
C97–C102	1.427(6)	C133–C138	1.434(6)
C97–C98	1.435(6)	C134–C135	1.373(6)
C98–C99	1.354(6)	C135–C136	1.426(6)
C99–C100	1.424(6)	C136–C137	1.423(6)
C100–C101	1.418(6)	C136–C139	1.433(6)
C100–C103	1.435(6)	C137–C138	1.377(6)
C101–C102	1.374(6)	C139–C140	1.411(6)
C103–C104	1.430(6)	C139–C144	1.439(6)
C103–C108	1.433(6)	C140–C141	1.363(6)
C104–C105	1.368(6)	C141–C142	1.427(6)
C105–C106	1.428(6)	C142–C143	1.417(6)
C106–C107	1.417(7)	C143–C144	1.383(6)
C107–C108	1.371(6)	B1–F4	1.361(6)
N13–C142	1.361(5)	B1–F3	1.385(7)

B1–F1	1.394(6)	B8C–F30C	1.391(11)
B1–F2	1.398(6)	B8C–F31C	1.410(12)
B2–F8	1.380(6)	B8C–F29C	1.418(11)
B2–F7	1.382(7)	O1A–C22A	1.260(12)
B2–F6	1.394(6)	C21A–C22A	1.488(13)
B2–F5	1.396(6)	C22A–C23A	1.455(13)
B3A–F12A	1.352(9)	O1B–C22B	1.290(11)
B3A–F10A	1.392(9)	C21B–C22B	1.478(12)
B3A–F11A	1.356(10)	C22B–C23B	1.446(12)
B3A–F9A	1.442(10)	O2–C302	1.294(8)
B3B–F12B	1.367(12)	C301–C302	1.443(9)
B3B–F10B	1.390(12)	C302–C303	1.459(9)
B3B–F11B	1.393(13)	C34–N1–C6	120.9(4)
B3B–F9B	1.414(12)	C34–N1–C1	122.9(4)
B4–F14	1.334(7)	C6–N1–C1	116.2(4)
B4–F16	1.373(7)	C7–N2–C4	121.9(4)
B4–F13	1.380(8)	C7–N2–C3	122.5(4)
B4–F15	1.426(8)	C4–N2–C3	115.6(4)
B5–F18	1.377(6)	C16–N3–C19	121.4(4)
B5–F20	1.378(6)	C16–N3–C24	122.9(4)
B5–F19	1.393(6)	C19–N3–C24	115.6(4)
B5–F17	1.405(6)	C25–N4–C21	122.2(4)
B6A–F24A	1.372(11)	C25–N4–C22	123.3(4)
B6A–F22A	1.395(12)	C21–N4–C22	114.4(4)
B6A–F23A	1.391(11)	N1–C1–C2	112.8(4)
B6A–F21A	1.418(11)	C1–C2–C3	114.9(5)
B6B–F24B	1.369(8)	N2–C3–C2	112.1(4)
B6B–F23B	1.391(9)	N2–C4–C5	113.3(4)
B6B–F22B	1.398(9)	C6–C5–C4	115.1(4)
B6B–F21B	1.403(8)	N1–C6–C5	113.2(4)
B7A–F26A	1.387(9)	N2–C7–C12	120.6(4)
B7A–F27A	1.391(10)	N2–C7–C8	122.5(4)
B7A–F28A	1.394(10)	C12–C7–C8	116.8(4)
B7A–F25A	1.401(10)	C9–C8–C7	120.8(4)
B7B–F28B	1.374(8)	C8–C9–C10	122.8(4)
B7B–F26B	1.385(8)	C9–C10–C11	116.0(4)
B7B–F27B	1.396(8)	C9–C10–C13	122.4(4)
B7B–F25B	1.407(9)	C11–C10–C13	121.6(4)
B8A–F32A	1.388(12)	C12–C11–C10	122.3(4)
B8A–F30A	1.398(13)	C11–C12–C7	120.9(4)
B8A–F31A	1.396(12)	C18–C13–C14	115.4(4)
B8A–F29A	1.418(12)	C18–C13–C10	122.4(4)
B8B–F30B	1.374(10)	C14–C13–C10	122.2(4)
B8B–F32B	1.388(10)	C15–C14–C13	122.8(4)
B8B–F31B	1.400(10)	C14–C15–C16	121.2(4)
B8B–F29B	1.406(11)	N3–C16–C15	124.1(4)
B8C–F32C	1.381(11)	N3–C16–C17	120.3(4)

C15–C16–C17	115.6(4)	N6–C43–C48	121.1(4)
C18–C17–C16	121.4(4)	N6–C43–C44	122.3(4)
C17–C18–C13	123.3(4)	C48–C43–C44	116.5(4)
N3–C19–C20	114.4(4)	C45–C44–C43	120.8(4)
C19–C20–C21	115.3(4)	C44–C45–C46	122.6(4)
N4–C21–C20	113.3(4)	C47–C46–C45	115.4(4)
N4–C22–C23	112.6(4)	C47–C46–C49	122.7(4)
C24–C23–C22	113.7(4)	C45–C46–C49	121.8(4)
N3–C24–C23	112.6(4)	C48–C47–C46	123.4(4)
N4–C25–C26	121.4(4)	C47–C48–C43	120.6(4)
N4–C25–C30	122.6(4)	C50–C49–C54	115.3(4)
C26–C25–C30	115.9(4)	C50–C49–C46	122.6(4)
C27–C26–C25	122.1(4)	C54–C49–C46	122.1(4)
C26–C27–C28	122.4(4)	C51–C50–C49	123.2(5)
C29–C28–C27	115.2(4)	C50–C51–C52	120.7(5)
C29–C28–C31	122.4(4)	N7–C52–C51	123.3(5)
C27–C28–C31	122.3(4)	N7–C52–C53	120.0(5)
C30–C29–C28	123.3(4)	C51–C52–C53	116.7(4)
C29–C30–C25	120.8(4)	C54–C53–C52	121.6(5)
C32–C31–C36	115.5(4)	C53–C54–C49	122.3(5)
C32–C31–C28	122.0(4)	N7–C55–C56	115.0(5)
C36–C31–C28	122.5(4)	C57–C56–C55	110.2(7)
C33–C32–C31	122.3(4)	N8–C57–C56	114.6(5)
C32–C33–C34	121.2(5)	N8–C58–C59	108.8(5)
N1–C34–C35	122.5(4)	C60–C59–C58	113.2(5)
N1–C34–C33	120.8(4)	N7–C60–C59	109.5(5)
C35–C34–C33	116.7(4)	N8–C61–C66	121.7(5)
C36–C35–C34	121.1(4)	N8–C61–C62	122.0(4)
C35–C36–C31	122.8(4)	C66–C61–C62	116.3(4)
C70–N5–C37	124.3(4)	C63–C62–C61	121.8(4)
C70–N5–C42	120.7(3)	C62–C63–C64	122.5(4)
C37–N5–C42	114.9(3)	C65–C64–C63	114.8(4)
C43–N6–C39	122.4(4)	C65–C64–C67	122.4(4)
C43–N6–C40	121.2(4)	C63–C64–C67	122.8(4)
C39–N6–C40	116.0(3)	C66–C65–C64	122.9(4)
C52–N7–C55	122.0(5)	C65–C66–C61	121.2(4)
C52–N7–C60	121.6(5)	C68–C67–C72	115.4(4)
C55–N7–C60	116.3(5)	C68–C67–C64	122.7(4)
C61–N8–C58	123.6(4)	C72–C67–C64	121.9(4)
C61–N8–C57	120.4(5)	C69–C68–C67	122.5(4)
C58–N8–C57	116.0(4)	C68–C69–C70	122.1(4)
N5–C37–C38	113.2(4)	N5–C70–C69	121.7(4)
C39–C38–C37	113.6(4)	N5–C70–C71	121.9(4)
N6–C39–C38	113.2(4)	C69–C70–C71	116.4(4)
N6–C40–C41	114.0(4)	C72–C71–C70	121.2(4)
C42–C41–C40	115.5(4)	C71–C72–C67	122.5(4)
N5–C42–C41	113.8(4)	C106–N9–C73	121.4(4)

C106–N9–C78	123.4(4)	C98–C99–C100	121.6(4)
C73–N9–C78	115.1(4)	C101–C100–C99	115.6(4)
C79–N10–C75	121.6(4)	C101–C100–C103	121.9(4)
C79–N10–C76	123.5(4)	C99–C100–C103	122.5(4)
C75–N10–C76	114.8(4)	C102–C101–C100	123.2(4)
C88–N11–C96	122.4(4)	C101–C102–C97	121.1(4)
C88–N11–C91	122.8(4)	C104–C103–C108	115.1(4)
C96–N11–C91	114.7(4)	C104–C103–C100	123.3(4)
C97–N12–C93	124.5(4)	C108–C103–C100	121.6(4)
C97–N12–C94	121.5(4)	C105–C104–C103	122.5(4)
C93–N12–C94	114.0(4)	C104–C105–C106	121.4(4)
N9–C73–C74	114.2(4)	N9–C106–C107	120.4(4)
C75–C74–C73	115.3(5)	N9–C106–C105	123.3(4)
N10–C75–C74	113.3(4)	C107–C106–C105	116.3(4)
N10–C76–C77	113.3(4)	C108–C107–C106	121.7(4)
C76–C77–C78	114.0(4)	C107–C108–C103	122.3(4)
N9–C78–C77	113.1(4)	C142–N13–C109	123.0(3)
N10–C79–C80	122.1(4)	C142–N13–C112	121.4(3)
N10–C79–C84	122.5(4)	C109–N13–C112	115.1(3)
C80–C79–C84	115.3(4)	C115–N14–C114	122.2(3)
C81–C80–C79	121.8(4)	C115–N14–C111	123.8(3)
C80–C81–C82	122.8(4)	C114–N14–C111	113.9(3)
C83–C82–C81	114.9(4)	C124–N15–C127	122.3(3)
C83–C82–C85	122.6(4)	C124–N15–C132	123.4(3)
C81–C82–C85	122.5(4)	C127–N15–C132	114.3(3)
C84–C83–C82	123.3(4)	C133–N16–C129	121.8(3)
C83–C84–C79	121.7(4)	C133–N16–C130	123.1(3)
C86–C85–C90	115.2(4)	C129–N16–C130	114.9(3)
C86–C85–C82	123.4(4)	N13–C109–C110	113.0(3)
C90–C85–C82	121.4(4)	C109–C110–C111	113.7(3)
C87–C86–C85	122.4(4)	N14–C111–C110	112.4(3)
C86–C87–C88	121.6(4)	N13–C112–C113	113.8(3)
N11–C88–C89	120.9(4)	C112–C113–C114	115.3(4)
N11–C88–C87	123.5(4)	N14–C114–C113	113.1(3)
C89–C88–C87	115.6(4)	N14–C115–C120	121.0(4)
C90–C89–C88	121.5(4)	N14–C115–C116	123.4(4)
C89–C90–C85	123.1(4)	C120–C115–C116	115.6(4)
N11–C91–C92	113.8(4)	C117–C116–C115	121.4(4)
C91–C92–C93	113.4(4)	C116–C117–C118	123.0(4)
N12–C93–C92	113.1(4)	C117–C118–C119	115.4(3)
N12–C94–C95	113.3(4)	C117–C118–C121	122.4(4)
C94–C95–C96	115.5(4)	C119–C118–C121	122.2(4)
N11–C96–C95	115.3(4)	C120–C119–C118	122.4(4)
N12–C97–C102	122.5(4)	C119–C120–C115	122.0(4)
N12–C97–C98	122.3(4)	C122–C121–C126	115.1(4)
C102–C97–C98	115.2(4)	C122–C121–C118	122.7(4)
C99–C98–C97	123.3(4)	C126–C121–C118	122.3(4)

C123–C122–C121	123.5(4)	F10A–B3A–F11A	106.9(8)
C122–C123–C124	120.8(4)	F12A–B3A–F9A	105.3(8)
N15–C124–C123	120.9(4)	F10A–B3A–F9A	112.3(8)
N15–C124–C125	122.5(4)	F11A–B3A–F9A	103.8(7)
C123–C124–C125	116.6(4)	F12B–B3B–F10B	110.7(12)
C126–C125–C124	121.0(4)	F12B–B3B–F11B	111.5(13)
C125–C126–C121	122.9(4)	F10B–B3B–F11B	105.1(12)
N15–C127–C128	114.1(3)	F12B–B3B–F9B	108.9(12)
C129–C128–C127	116.1(4)	F10B–B3B–F9B	112.1(13)
N16–C129–C128	113.9(3)	F11B–B3B–F9B	108.6(12)
N16–C130–C131	113.2(4)	F14–B4–F16	112.7(6)
C132–C131–C130	114.4(4)	F14–B4–F13	112.9(6)
N15–C132–C131	111.9(3)	F16–B4–F13	110.7(6)
N16–C133–C134	123.1(4)	F14–B4–F15	107.3(6)
N16–C133–C138	121.5(4)	F16–B4–F15	110.6(6)
C134–C133–C138	115.5(4)	F13–B4–F15	102.1(5)
C135–C134–C133	122.3(4)	F18–B5–F20	110.4(4)
C134–C135–C136	122.5(4)	F18–B5–F19	109.3(4)
C137–C136–C135	115.0(4)	F20–B5–F19	108.7(5)
C137–C136–C139	123.5(4)	F18–B5–F17	111.0(5)
C135–C136–C139	121.5(4)	F20–B5–F17	107.9(4)
C138–C137–C136	122.8(4)	F19–B5–F17	109.6(4)
C137–C138–C133	121.5(4)	F24A–B6A–F22A	114.1(11)
C140–C139–C136	123.2(4)	F24A–B6A–F23A	111.0(10)
C140–C139–C144	114.5(4)	F22A–B6A–F23A	106.6(10)
C136–C139–C144	122.3(4)	F24A–B6A–F21A	106.8(9)
C141–C140–C139	124.0(4)	F22A–B6A–F21A	107.2(9)
C140–C141–C142	121.2(4)	F23A–B6A–F21A	111.1(10)
N13–C142–C143	122.8(4)	F24B–B6B–F23B	110.1(6)
N13–C142–C141	120.9(4)	F24B–B6B–F22B	112.5(6)
C143–C142–C141	116.2(4)	F23B–B6B–F22B	106.4(6)
C144–C143–C142	121.7(4)	F24B–B6B–F21B	111.5(6)
C143–C144–C139	122.0(4)	F23B–B6B–F21B	108.2(6)
F4–B1–F3	109.8(5)	F22B–B6B–F21B	108.0(6)
F4–B1–F1	109.9(4)	F26A–B7A–F27A	110.6(7)
F3–B1–F1	110.4(4)	F26A–B7A–F28A	109.6(8)
F4–B1–F2	108.3(4)	F27A–B7A–F28A	109.4(7)
F3–B1–F2	107.4(4)	F26A–B7A–F25A	110.3(7)
F1–B1–F2	111.0(5)	F27A–B7A–F25A	108.0(8)
F8–B2–F7	110.4(5)	F28A–B7A–F25A	108.8(7)
F8–B2–F6	110.2(4)	F28B–B7B–F26B	112.0(7)
F7–B2–F6	110.3(4)	F28B–B7B–F27B	108.8(6)
F8–B2–F5	107.8(4)	F26B–B7B–F27B	108.4(6)
F7–B2–F5	108.7(4)	F28B–B7B–F25B	110.1(6)
F6–B2–F5	109.4(4)	F26B–B7B–F25B	110.5(6)
F12A–B3A–F10A	112.5(7)	F27B–B7B–F25B	107.0(6)
F12A–B3A–F11A	115.8(8)	F32A–B8A–F30A	108.3(12)

F32A–B8A–F31A	105.3(12)
F30A–B8A–F31A	108.6(12)
F32A–B8A–F29A	110.9(12)
F30A–B8A–F29A	111.7(12)
F31A–B8A–F29A	111.7(13)
F30B–B8B–F32B	113.9(8)
F30B–B8B–F31B	110.9(8)
F32B–B8B–F31B	113.9(9)
F30B–B8B–F29B	108.5(9)
F32B–B8B–F29B	107.0(8)
F31B–B8B–F29B	101.8(8)
F32C–B8C–F30C	105.5(9)
F32C–B8C–F31C	104.8(10)
F30C–B8C–F31C	109.0(10)
F32C–B8C–F29C	109.9(10)
F30C–B8C–F29C	109.3(10)
F31C–B8C–F29C	117.6(10)
O1A–C22A–C23A	126.5(12)
O1A–C22A–C21A	123.9(12)
C23A–C22A–C21A	109.3(11)
O1B–C22B–C23B	117.3(10)
O1B–C22B–C21B	126.7(10)
C23B–C22B–C21B	116.0(10)
O2–C302–C301	120.5(7)
O2–C302–C303	121.1(7)
C301–C302–C303	118.4(6)

Table S 4: Anisotropic displacement parameters [Å^2× 10^3]. The anisotropic displacement factor exponent takes the form: $-2\pi^2[h^2a^{*2}U^{11} + ... + 2\,h\,k\,a^*\,b^*\,U^{12}]$.

Atom	U^{11}	U^{22}	U^{33}	U^{23}	U^{13}	U^{12}
N1	32(2)	37(2)	32(2)	−9(2)	−9(2)	−2(2)
N2	26(2)	25(2)	32(2)	−2(2)	−9(2)	0(2)
N3	30(2)	38(2)	25(2)	5(2)	−9(1)	−11(2)
N4	25(2)	38(2)	26(2)	4(2)	−2(2)	−4(2)
C1	29(2)	43(3)	38(2)	−3(2)	−12(2)	−1(2)
C2	43(3)	71(4)	61(4)	−6(3)	−14(3)	7(3)
C3	23(2)	36(3)	43(3)	−8(2)	−6(2)	1(2)
C4	31(2)	33(3)	44(3)	−3(2)	−13(2)	−7(2)
C5	37(3)	34(3)	52(3)	−13(2)	−12(2)	−6(2)
C6	36(3)	44(3)	38(2)	−11(2)	−13(2)	5(2)
C7	18(2)	33(2)	20(2)	−4(2)	−2(2)	−2(2)
C8	35(2)	25(2)	30(2)	−9(2)	−9(2)	6(2)
C9	29(2)	24(2)	32(2)	−6(2)	−15(2)	−3(2)
C10	24(2)	21(2)	18(2)	−1(2)	−2(2)	−1(2)
C11	26(2)	18(2)	34(2)	0(2)	−7(2)	−1(2)
C12	29(2)	18(2)	35(2)	6(2)	−6(2)	−4(2)
C13	25(2)	21(2)	18(2)	2(2)	−2(2)	−5(2)
C14	30(2)	22(2)	23(2)	1(2)	−2(2)	−3(2)
C15	32(2)	21(2)	25(2)	2(2)	−5(2)	−3(2)
C16	31(2)	30(2)	22(2)	−1(2)	−5(2)	−11(2)
C17	29(2)	22(2)	30(2)	2(2)	−7(2)	−2(2)
C18	30(2)	23(2)	27(2)	1(2)	−5(2)	−6(2)
C19	22(2)	39(3)	38(2)	8(2)	−11(2)	−5(2)
C20	23(2)	40(3)	44(3)	8(2)	−8(2)	−3(2)
C21	27(2)	42(3)	41(3)	−4(2)	−10(2)	−6(2)
C22	27(2)	41(3)	31(2)	6(2)	−2(2)	−12(2)
C23	36(3)	45(3)	35(2)	−4(2)	−4(2)	−13(2)
C24	38(2)	41(3)	32(2)	−5(2)	−4(2)	−21(2)
C25	24(2)	38(3)	20(2)	−3(2)	−1(2)	−2(2)
C26	27(2)	35(3)	24(2)	−9(2)	0(2)	4(2)
C27	33(2)	31(2)	26(2)	−9(2)	−1(2)	−5(2)
C28	27(2)	39(3)	16(2)	−3(2)	−2(2)	−1(2)
C29	32(2)	32(3)	24(2)	−3(2)	−4(2)	1(2)
C30	27(2)	34(3)	26(2)	−2(2)	2(2)	−6(2)
C31	30(2)	32(2)	19(2)	−2(2)	−2(2)	−1(2)
C32	30(2)	43(3)	23(2)	−6(2)	−3(2)	1(2)
C33	34(2)	38(3)	27(2)	−11(2)	−4(2)	−2(2)
C34	30(2)	40(3)	18(2)	−4(2)	−6(2)	3(2)
C35	26(2)	41(3)	25(2)	−6(2)	−6(2)	9(2)
C36	34(2)	32(2)	25(2)	−9(2)	−5(2)	−2(2)

N5	26(2)	25(2)	26(2)	−2(2)	−5(1)	−7(1)
N6	18(2)	22(2)	28(2)	1(2)	4(1)	−2(1)
N7	31(2)	99(4)	28(2)	−2(2)	−7(2)	−18(2)
N8	21(2)	74(3)	33(2)	−4(2)	−3(2)	−9(2)
C37	21(2)	38(3)	29(2)	5(2)	−8(2)	−5(2)
C38	24(2)	29(2)	40(2)	3(2)	−5(2)	−4(2)
C39	18(2)	33(2)	32(2)	−2(2)	−3(2)	1(2)
C40	25(2)	29(2)	44(3)	4(2)	0(2)	−10(2)
C41	30(2)	17(2)	53(3)	−2(2)	1(2)	−11(2)
C42	22(2)	40(3)	43(3)	−9(2)	0(2)	−12(2)
C43	24(2)	24(2)	21(2)	3(2)	7(2)	−2(2)
C44	28(2)	22(2)	29(2)	1(2)	−5(2)	1(2)
C45	33(2)	24(2)	32(2)	4(2)	−10(2)	−4(2)
C46	27(2)	34(2)	18(2)	0(2)	−3(2)	−5(2)
C47	30(2)	32(3)	30(2)	6(2)	−1(2)	−2(2)
C48	31(2)	19(2)	34(2)	6(2)	−7(2)	−3(2)
C49	33(2)	41(3)	18(2)	5(2)	−5(2)	−8(2)
C50	28(2)	45(3)	27(2)	0(2)	−2(2)	−7(2)
C51	38(3)	64(3)	25(2)	0(2)	−6(2)	−17(2)
C52	23(2)	74(4)	23(2)	−3(2)	−6(2)	−12(2)
C53	39(3)	62(4)	30(2)	11(2)	−11(2)	0(3)
C54	30(2)	52(3)	29(2)	14(2)	−8(2)	−7(2)
C55	31(3)	135(7)	44(3)	17(4)	−17(2)	0(3)
C56	58(4)	93(6)	81(5)	0(4)	−22(3)	12(4)
C57	24(3)	114(6)	50(3)	0(4)	−6(2)	9(3)
C58	36(2)	94(4)	48(3)	−9(3)	−4(2)	−42(2)
C59	52(3)	122(6)	67(4)	3(4)	−22(3)	−35(4)
C60	45(3)	126(5)	38(3)	−27(3)	−2(2)	−42(3)
C61	19(2)	45(3)	23(2)	3(2)	−2(2)	−6(2)
C62	24(2)	37(3)	41(3)	0(2)	−3(2)	4(2)
C63	26(2)	32(3)	35(2)	1(2)	−3(2)	−6(2)
C64	21(2)	24(2)	18(2)	−5(2)	2(2)	−5(2)
C65	27(2)	26(2)	38(2)	−7(2)	−8(2)	−3(2)
C66	32(2)	34(3)	39(2)	−8(2)	−4(2)	−9(2)
C67	23(2)	20(2)	14(2)	−4(2)	1(2)	1(2)
C68	29(2)	21(2)	24(2)	−6(2)	−4(2)	−1(2)
C69	29(2)	17(2)	33(2)	−4(2)	1(2)	−6(2)
C70	23(2)	23(2)	18(2)	−1(2)	−1(2)	−6(2)
C71	27(2)	21(2)	34(2)	−5(2)	−3(2)	2(2)
C72	24(2)	24(2)	33(2)	−7(2)	−3(2)	−6(2)
N9	31(2)	21(2)	54(2)	−3(2)	−5(2)	−4(2)
N10	37(2)	26(2)	44(2)	−8(2)	−2(2)	−12(2)
N11	33(2)	32(2)	44(2)	8(2)	−10(2)	0(2)
N12	29(2)	27(2)	32(2)	−2(2)	−6(2)	−1(2)
C73	23(2)	32(3)	74(4)	−13(3)	4(2)	−3(2)
C74	34(3)	42(3)	57(3)	−15(2)	15(2)	−14(2)
C75	42(3)	37(3)	47(3)	−6(2)	7(2)	−17(2)

C76	34(2)	44(3)	50(3)	−12(2)	−8(2)	−11(2)
C77	31(2)	39(3)	66(3)	−6(3)	−13(2)	−10(2)
C78	39(3)	34(3)	58(3)	6(2)	−22(2)	−6(2)
C79	39(2)	19(2)	36(2)	−3(2)	−2(2)	−10(2)
C80	43(3)	30(2)	30(2)	−5(2)	2(2)	−11(2)
C81	49(3)	24(2)	30(2)	−2(2)	−3(2)	−16(2)
C82	34(2)	15(2)	30(2)	0(2)	1(2)	−2(2)
C83	35(2)	28(2)	28(2)	−2(2)	−2(2)	−7(2)
C84	41(3)	19(2)	35(2)	−6(2)	−8(2)	−5(2)
C85	39(2)	16(2)	35(2)	−1(2)	−4(2)	−5(2)
C86	39(2)	23(2)	29(2)	8(2)	−5(2)	−2(2)
C87	43(2)	21(2)	32(2)	6(2)	−18(2)	−2(2)
C88	38(2)	19(2)	45(3)	8(2)	−13(2)	1(2)
C89	33(2)	26(2)	38(2)	−3(2)	6(2)	−1(2)
C90	44(3)	23(2)	31(2)	−1(2)	−6(2)	−4(2)
C91	38(3)	47(3)	55(3)	13(3)	−26(2)	−8(2)
C92	36(3)	61(4)	63(3)	2(3)	−28(2)	−10(2)
C93	28(2)	40(3)	44(3)	−1(2)	−14(2)	−7(2)
C94	28(2)	35(3)	40(3)	4(2)	−2(2)	−6(2)
C95	31(3)	42(3)	50(3)	−4(2)	6(2)	−2(2)
C96	29(2)	28(3)	63(3)	3(2)	−2(2)	6(2)
C97	24(2)	18(2)	32(2)	−2(2)	−6(2)	0(2)
C98	30(2)	21(2)	28(2)	−4(2)	2(2)	−4(2)
C99	26(2)	16(2)	24(2)	−2(2)	−1(2)	1(2)
C100	30(2)	12(2)	23(2)	1(2)	−5(2)	−1(2)
C101	33(2)	26(2)	24(2)	1(2)	2(2)	−2(2)
C102	32(2)	27(2)	21(2)	4(2)	−6(2)	−5(2)
C103	26(2)	15(2)	28(2)	−1(2)	−2(2)	1(2)
C104	30(2)	24(2)	30(2)	−1(2)	−2(2)	−2(2)
C105	35(2)	27(2)	37(2)	−6(2)	−10(2)	−4(2)
C106	29(2)	14(2)	43(2)	−2(2)	−4(2)	−1(2)
C107	30(2)	20(2)	41(2)	−8(2)	11(2)	−7(2)
C108	35(2)	21(2)	32(2)	−6(2)	0(2)	−5(2)
N13	16(2)	16(2)	32(2)	−1(1)	−6(1)	3(1)
N14	18(2)	18(2)	30(2)	2(1)	−8(1)	−5(1)
N15	18(2)	21(2)	33(2)	−4(2)	−7(1)	−2(1)
N16	24(2)	20(2)	24(2)	0(1)	−6(1)	−5(1)
C109	18(2)	26(2)	37(2)	−10(2)	0(2)	−1(2)
C110	17(2)	27(2)	42(2)	0(2)	−7(2)	−7(2)
C111	15(2)	28(2)	33(2)	2(2)	−6(2)	−8(2)
C112	19(2)	29(2)	38(2)	2(2)	−14(2)	1(2)
C113	28(2)	28(2)	31(2)	1(2)	−12(2)	−5(2)
C114	18(2)	24(2)	33(2)	−7(2)	−6(2)	−3(2)
C115	18(2)	14(2)	31(2)	−2(2)	−4(2)	−2(2)
C116	20(2)	20(2)	23(2)	−1(2)	−2(2)	−2(2)
C117	18(2)	19(2)	25(2)	−1(2)	−5(2)	−1(2)
C118	17(2)	17(2)	27(2)	−4(2)	−5(2)	−4(2)

C119	17(2)	22(2)	26(2)	1(2)	−5(2)	−4(2)
C120	22(2)	22(2)	24(2)	−2(2)	−5(2)	1(2)
C121	23(2)	11(2)	29(2)	−3(2)	−7(2)	0(2)
C122	15(2)	23(2)	27(2)	−3(2)	−2(2)	−2(2)
C123	18(2)	21(2)	28(2)	−2(2)	−9(2)	1(2)
C124	18(2)	17(2)	29(2)	−6(2)	−4(2)	1(2)
C125	22(2)	18(2)	30(2)	−1(2)	−2(2)	0(2)
C126	23(2)	19(2)	26(2)	3(2)	−9(2)	−3(2)
C127	21(2)	27(2)	36(2)	−1(2)	−12(2)	−6(2)
C128	24(2)	25(2)	30(2)	−1(2)	−16(2)	−8(2)
C129	21(2)	24(2)	30(2)	−4(2)	−6(2)	−3(2)
C130	26(2)	25(2)	33(2)	3(2)	−1(2)	−11(2)
C131	19(2)	36(3)	35(2)	−2(2)	−2(2)	−7(2)
C132	15(2)	45(3)	38(2)	−12(2)	2(2)	−8(2)
C133	27(2)	11(2)	26(2)	−5(2)	−8(2)	−3(2)
C134	22(2)	27(2)	29(2)	−1(2)	1(2)	−6(2)
C135	27(2)	23(2)	25(2)	−2(2)	−8(2)	−2(2)
C136	21(2)	9(2)	27(2)	2(2)	−4(2)	0(2)
C137	19(2)	20(2)	28(2)	−2(2)	0(2)	−5(2)
C138	27(2)	22(2)	20(2)	−5(2)	−6(2)	−4(2)
C139	21(2)	15(2)	30(2)	−6(2)	−4(2)	3(2)
C140	26(2)	16(2)	30(2)	−1(2)	−6(2)	−3(2)
C141	24(2)	19(2)	30(2)	−2(2)	−6(2)	−3(2)
C142	17(2)	12(2)	36(2)	−6(2)	−2(2)	3(2)
C143	18(2)	23(2)	31(2)	−8(2)	3(2)	−3(2)
C144	25(2)	27(2)	27(2)	−6(2)	−4(2)	1(2)
B1	52(2)	36(2)	65(2)	−9(2)	−13(2)	−16(2)
F1	52(2)	30(1)	73(2)	−5(1)	−14(2)	1(1)
F2	71(2)	53(2)	61(2)	−8(2)	−16(1)	−16(2)
F3	124(2)	101(2)	77(2)	−34(2)	27(2)	−78(2)
F4	80(2)	38(2)	144(3)	−25(2)	−64(2)	9(2)
B2	35(2)	60(3)	47(2)	2(2)	−17(2)	−1(2)
F5	36(1)	59(2)	37(1)	−2(1)	−3(1)	−10(1)
F6	59(2)	64(2)	60(2)	−6(2)	−27(2)	18(2)
F7	52(2)	65(2)	119(3)	4(2)	−36(2)	−17(2)
F8	64(2)	112(3)	41(2)	10(2)	7(2)	25(2)
B3A	228(2)	53(2)	44(2)	5(2)	−5(2)	2(2)
F9A	233(3)	83(3)	56(2)	7(2)	−1(2)	−13(2)
F10A	248(4)	40(2)	60(3)	8(2)	5(3)	3(3)
F11A	221(3)	53(3)	47(2)	1(2)	−16(2)	16(3)
F12A	219(4)	54(2)	39(2)	4(2)	−11(2)	−3(3)
B3B	229(2)	56(2)	47(2)	6(2)	−4(2)	1(2)
F9B	227(3)	54(4)	49(4)	7(3)	−2(3)	0(4)
F10B	233(4)	56(3)	51(3)	4(3)	−2(3)	−2(3)
F11B	231(3)	62(4)	51(3)	9(3)	−6(3)	4(3)
F12B	229(4)	55(3)	43(4)	6(3)	−3(4)	0(3)
B4	107(3)	68(3)	54(2)	−10(3)	−15(3)	−6(3)

F13	148(3)	78(2)	67(2)	9(2)	−35(2)	−40(2)
F14	92(3)	124(3)	45(2)	−21(2)	−9(2)	7(2)
F15	117(2)	83(3)	66(2)	−3(2)	9(2)	16(2)
F16	121(3)	51(2)	70(2)	−15(2)	−1(2)	2(2)
B5	38(2)	40(2)	66(3)	3(2)	−11(2)	−15(2)
F17	75(2)	73(2)	75(2)	−15(2)	11(2)	−33(2)
F18	50(1)	52(2)	70(2)	−6(2)	−28(1)	−15(1)
F19	42(2)	41(2)	115(3)	16(2)	1(2)	−3(1)
F20	42(1)	51(2)	58(2)	4(1)	−27(1)	−12(1)
B6A	97(2)	42(2)	38(2)	2(2)	0(2)	−5(2)
F21A	103(3)	54(4)	39(3)	7(3)	3(3)	−9(3)
F22A	99(3)	57(4)	48(3)	2(3)	−6(2)	−1(3)
F23A	102(4)	41(3)	43(3)	0(2)	3(3)	0(3)
F24A	96(4)	37(3)	38(4)	−2(3)	−2(3)	−10(3)
B6B	94(2)	40(2)	36(2)	2(2)	0(2)	−6(2)
F21B	103(3)	45(2)	31(2)	2(2)	3(2)	5(2)
F22B	91(2)	62(3)	58(2)	6(2)	2(2)	0(2)
F23B	95(3)	30(2)	36(2)	3(2)	−5(2)	−3(2)
F24B	103(3)	32(2)	35(2)	−3(2)	0(2)	−6(2)
B7A	36(2)	42(2)	38(2)	2(2)	−9(2)	−10(2)
F25A	52(3)	49(2)	51(3)	15(2)	−13(2)	−17(2)
F26A	35(2)	47(3)	43(3)	−11(3)	−14(2)	−3(2)
F27A	35(3)	42(3)	37(2)	4(2)	−9(2)	−9(3)
F28A	42(3)	50(3)	36(2)	−3(2)	−3(2)	−2(2)
B7B	35(2)	40(2)	36(2)	1(2)	−8(2)	−11(2)
F25B	30(2)	34(2)	55(3)	9(2)	−10(2)	−11(2)
F26B	37(2)	44(3)	46(2)	−5(2)	−18(2)	−13(2)
F27B	38(2)	39(2)	36(2)	4(2)	−13(2)	−17(2)
F28B	66(3)	59(3)	47(2)	3(2)	15(2)	11(3)
B8A	118(2)	50(2)	45(2)	4(2)	13(2)	−9(2)
F29A	118(3)	54(3)	50(3)	0(3)	10(2)	−11(3)
F30A	119(3)	46(3)	41(3)	6(2)	12(3)	−6(3)
F31A	114(3)	52(3)	45(3)	3(3)	16(3)	−12(3)
F32A	118(3)	48(3)	47(3)	4(3)	15(3)	−11(3)
B8B	117(2)	49(2)	44(2)	5(2)	13(2)	−9(2)
F29B	126(3)	64(3)	55(3)	−1(2)	9(2)	−18(2)
F30B	123(3)	42(3)	42(3)	11(2)	11(2)	−1(2)
F31B	109(3)	48(2)	41(2)	5(2)	20(2)	−4(2)
F32B	113(2)	51(2)	45(2)	3(2)	13(2)	−6(2)
B8C	117(2)	48(2)	44(2)	2(2)	12(2)	−9(2)
F29C	129(4)	38(4)	40(3)	−11(3)	2(3)	−5(3)
F31C	116(2)	54(2)	53(3)	−2(2)	13(2)	−12(2)
F30C	123(3)	45(2)	41(3)	4(2)	12(3)	−6(2)
F32C	123(3)	60(3)	46(2)	2(3)	14(3)	−11(3)
O1A	96(3)	69(2)	95(3)	−20(2)	4(2)	−32(2)
C21A	96(3)	69(2)	95(3)	−20(2)	4(2)	−32(2)
C22A	96(3)	69(2)	95(3)	−20(2)	4(2)	−32(2)

C23A	96(3)	69(2)	95(3)	−20(2)	4(2)	−32(2)
O1B	96(3)	69(2)	95(3)	−20(2)	4(2)	−32(2)
C21B	96(3)	69(2)	95(3)	−20(2)	4(2)	−32(2)
C22B	96(3)	69(2)	95(3)	−20(2)	4(2)	−32(2)
C23B	96(3)	69(2)	95(3)	−20(2)	4(2)	−32(2)
O2	119(5)	176(7)	62(3)	−1(4)	−29(3)	−1(5)
C301	94(6)	178(9)	62(4)	−34(5)	−12(4)	−43(5)
C302	49(4)	92(5)	73(4)	4(4)	−14(3)	−6(4)
C303	81(5)	114(7)	71(4)	−15(4)	−26(4)	4(5)

Table S 5: Hydrogen coordinates [× 10⁴] and isotropic displacement parameters [Å² × 10³].

Atom	*x*	*y*	*z*	U_{eq}	*S.o.f.*
H1A	–3536	10387	1849	44	1
H1B	–3126	10754	2343	44	1
H2A	–4263	9847	2719	71	1
H2B	–4507	10630	2762	71	1
H3A	–3667	10621	3581	41	1
H3B	–4338	10137	3791	41	1
H4A	–3897	9012	3408	42	1
H4B	–3185	8681	3836	42	1
H5A	–2168	8605	2923	48	1
H5B	–2922	8256	2784	48	1
H6A	–2455	8888	1867	47	1
H6B	–3413	9160	2124	47	1
H8	–2638	10759	3824	36	1
H9	–1354	10886	4056	33	1
H11	–633	8895	4147	31	1
H12	–1920	8749	3909	33	1
H14	–159	11009	4144	31	1
H15	1170	11151	4254	32	1
H17	1846	9136	4420	32	1
H18	516	9020	4311	32	1
H19A	2935	9331	4761	39	1
H19B	3655	9763	4523	39	1
H20A	2925	9019	3714	43	1
H20B	3869	8864	3869	43	1
H21A	4230	9759	3206	43	1
H21B	3773	9338	2793	43	1
H22A	2938	11222	3011	39	1
H22B	3888	10879	2814	39	1
H23A	4027	10572	3904	45	1
H23B	3724	11353	3839	45	1
H24A	2966	10911	4763	42	1
H24B	2339	11218	4257	42	1
H26	2555	9184	2684	36	1
H27	1266	9098	2428	36	1
H29	546	11077	2569	36	1
H30	1837	11179	2816	35	1
H32	86	9010	2253	39	1
H33	–1259	8895	2141	40	1
H35	–2003	10880	2335	39	1
H36	–665	10987	2460	36	1
H37A	6468	5297	210	35	1
H37B	6918	5748	598	35	1
H38A	5550	5751	1115	37	1
H38B	5696	4961	1171	37	1

H39A	6479	5777	1870	34	1
H39B	5752	5375	2182	34	1
H40A	6044	4193	1916	39	1
H40B	6746	3875	2357	39	1
H41A	7724	3618	1458	40	1
H41B	6899	3336	1364	40	1
H42A	7511	3840	387	41	1
H42B	6554	4160	590	41	1
H44	7440	5890	2224	32	1
H45	8741	5979	2460	35	1
H47	9341	3978	2620	38	1
H48	8048	3866	2387	34	1
H50	9952	6061	2566	40	1
H51	11312	6166	2614	49	1
H53	11918	4152	2766	54	1
H54	10554	4060	2723	45	1
H55A	13080	4326	3069	86	1
H55B	13797	4740	2768	86	1
H56A	13910	3789	2138	95	1
H56B	12954	3992	2007	95	1
H57A	14285	4721	1514	78	1
H57B	13847	4307	1080	78	1
H58A	12958	6198	1308	66	1
H58B	13900	5874	1045	66	1
H59A	14102	5465	2139	92	1
H59B	13918	6255	2087	92	1
H60A	13116	5925	3025	79	1
H60B	12478	6215	2519	79	1
H62	12575	4134	1049	42	1
H63	11264	4037	856	37	1
H65	10582	6033	888	36	1
H66	11894	6134	1093	41	1
H68	10046	3951	744	30	1
H69	8702	3857	649	32	1
H71	8016	5857	743	34	1
H72	9372	5949	842	32	1
H73A	–4314	3669	5334	53	1
H73B	–3830	4161	5637	53	1
H74A	–3105	3293	6220	54	1
H74B	–4079	3248	6374	54	1
H75A	–3235	2187	6215	50	1
H75B	–3923	2345	5728	50	1
H76A	–3275	1788	4751	50	1
H76B	–2541	2119	4335	50	1
H77A	–4183	2799	4763	53	1
H77B	–3832	2619	4030	53	1
H78A	–2894	3373	3967	51	1
H78B	–3831	3742	4165	51	1
H80	–2037	2386	6339	41	1

H81	−678	2450	6428	40	1
H83	−60	2328	4481	37	1
H84	−1412	2249	4378	38	1
H86	552	2591	6499	37	1
H87	1867	2827	6554	38	1
H89	2466	2597	4603	40	1
H90	1130	2424	4550	40	1
H91A	3802	2887	6288	54	1
H91B	2861	3222	6528	54	1
H92A	4100	3883	5768	61	1
H92B	3754	4002	6515	61	1
H93A	2429	4498	6243	44	1
H93B	3133	4877	5848	44	1
H94A	3809	4378	4849	42	1
H94B	3124	4575	4363	42	1
H95A	3976	3548	4128	51	1
H95B	3006	3487	4273	51	1
H96A	3746	2583	4782	51	1
H96B	4234	3043	5124	51	1
H98	1963	4260	4220	32	1
H99	638	4146	4101	27	1
H101	−34	4269	6049	35	1
H102	1307	4372	6179	32	1
H104	−561	3957	4016	34	1
H105	−1875	3748	3942	39	1
H107	−2571	4110	5857	38	1
H108	−1238	4267	5945	36	1
H10A	5752	1985	10709	32	1
H10B	6530	1583	11031	32	1
H11A	5721	1076	10084	34	1
H11B	5626	857	10838	34	1
H11C	7019	319	10743	30	1
H11D	6566	47	10210	30	1
H11E	6593	2453	9278	34	1
H11F	5963	1941	9502	34	1
H11G	7632	1623	8855	34	1
H11H	6798	1583	8555	34	1
H11I	6572	639	9197	29	1
H11J	7521	508	8854	29	1
H116	8129	435	10903	25	1
H117	9450	573	11042	25	1
H119	9988	983	9108	26	1
H120	8683	833	8957	28	1
H122	10620	707	11171	26	1
H123	11935	855	11330	27	1
H125	12463	1294	9387	29	1
H126	11153	1121	9245	27	1
H12A	13941	1280	11111	32	1
H12B	13237	840	11377	32	1
H12C	12269	1759	11684	30	1

H12D	13106	1816	11972	30	1
H12E	12479	2848	11549	30	1
H12F	13415	2636	11200	30	1
H13A	13377	3118	10070	33	1
H13B	12933	2697	9645	33	1
H13C	14359	2235	9656	36	1
H13D	14201	2139	10429	36	1
H13E	13541	1429	9541	39	1
H13F	14257	1120	9975	39	1
H134	11771	2780	9568	31	1
H135	10408	2717	9480	30	1
H137	9898	2514	11443	27	1
H138	11256	2604	11538	27	1
H140	9165	2726	9415	28	1
H141	7895	2487	9275	29	1
H143	7474	1948	11196	29	1
H144	8755	2205	11335	32	1
H21C	4716	1802	3355	128	0.450(8)
H21D	4849	2551	3398	128	0.450(8)
H21E	5643	1978	3269	128	0.450(8)
H23C	5746	2661	1741	128	0.450(8)
H23D	6137	2585	2409	128	0.450(8)
H23E	5352	3148	2313	128	0.450(8)
H21F	3918	2263	3142	128	0.550(8)
H21G	4243	2959	3062	128	0.550(8)
H21H	4773	2336	3400	128	0.550(8)
H23F	5954	2547	1957	128	0.550(8)
H23G	5399	3115	2393	128	0.550(8)
H23H	5131	2971	1715	128	0.550(8)
H30A	5612	2707	8310	161	1
H30B	4872	2287	8532	161	1
H30C	4724	2926	8053	161	1
H30D	5081	1611	6958	133	1
H30E	4381	2223	7192	133	1
H30F	4529	1571	7654	133	1

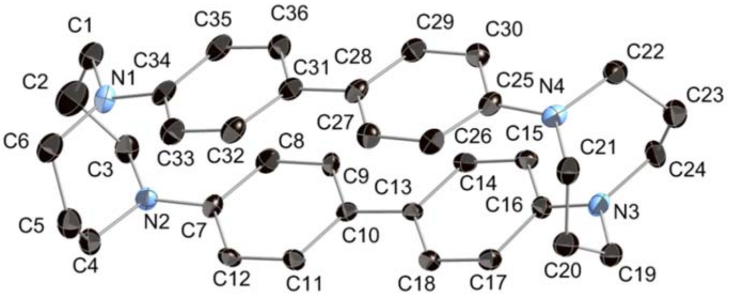

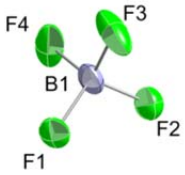

Thermal ellipsoids drawn at the 35% probability level, selected hydrogens and solvent omitted for clarity. Remainder of molecules in the asymmetric unit are labelled in a similar fashion.

9.2 Crystal from acetone solution

Table S 6: Crystal data and structure refinement details.

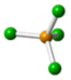
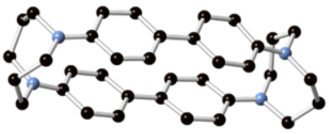
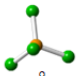

Identification code	**2012com0039** (SN205)	
Empirical formula	$C_{75}H_{86}B_4F_{16}N_8O$<br>$2(C_{36}H_{40}N_4^{2+})$, $4(BF_4^-)$, C_3H_6O	
Formula weight	1462.76	
Temperature	100(2) K	
Wavelength	0.71075 Å	
Crystal system	Triclinic	
Space group	*P*–1	
Unit cell dimensions	a = 16.340(2) Å	α = 86.397(5)°
	b = 20.486(3) Å	β = 82.004(5)°
	c = 20.799(3) Å	γ = 80.186(5)°
Volume	6788.2(16) $Å^3$	
Z	4	
Density (calculated)	1.431 Mg / m^3	
Absorption coefficient	0.115 mm^{-1}	
F(000)	3056	
Crystal	Fragment; Black	
Crystal size	0.22 × 0.18 × 0.04 mm^3	
θ range for data collection	2.96 – 25.03°	
Index ranges	$-18 \leq h \leq 19$, $-23 \leq k \leq 24$, $-24 \leq l \leq 24$	
Reflections collected	71433	
Independent reflections	23888 [R_{int} = 0.0652]	
Completeness to θ = 25.03°	99.6 %	
Absorption correction	Semi–empirical from equivalents	
Max. and min. transmission	0.9954 and 0.9751	
Refinement method	Full-matrix least-squares on F^2	
Data / restraints / parameters	23888 / 834 / 1869	
Goodness-of-fit on F^2	1.056	
Final R indices [$F^2 > 2\sigma(F^2)$]	$R1$ = 0.1154, $wR2$ = 0.2984	
R indices (all data)	$R1$ = 0.1571, $wR2$ = 0.3348	
Largest diff. peak and hole	1.801 and –0.886 e $Å^{-3}$	

Diffractometer: *Rigaku AFC12* goniometer equipped with an enhanced sensitivity (HG) *Saturn724+* detector mounted at the window of an *FR-E+ SuperBright* molybdenum rotating anode generator with HF *Varimax* optics (100µm focus). **Cell determination, Data collection, Data reduction and cell refinement & Absorption correction**: CrystalClear-SM Expert 2.0 r7 (Rigaku, 2011) , **Structure solution**: SHELXS97 (G. M. Sheldrick, Acta Cryst. (1990) A**46** 467–473). **Structure refinement**: SHELXL97 (G. M. Sheldrick (1997), University of Göttingen, Germany). **Graphics:** CrystalMaker: a crystal and molecular structures program for Mac and Windows. CrystalMaker Software Ltd, Oxford, England (www.crystalmaker.com)

Special details: DIFMX01_ALERT_2_A The maximum difference density is > 0.1*ZMAX*2.00 _refine_diff_density_max given = 1.801 (Test value = 1.800). There are several residual electron density peaks, the highest of which is 1.45A from F84B. These are associated with further disorder of the BF4 anions (2 of which are modelled as split over two positions) – further splitting to accommodate the residual electron density did not improve the refinement. All BF4 anions were modelled with 'Similar' geometries and thermal parameter restraints. The counter charge to the BF4's is believed to be located on the nitrogen atoms with partial charge delocalised/averaged across 2 of the 4 nitrogen atoms in each molecule.

Table S 7: Atomic coordinates [× 10^4], equivalent isotropic displacement parameters [$Å^2 \times 10^3$] and site occupancy factors. U_{eq} is defined as one third of the trace of the orthogonalized U^{ij} tensor.

Atom	*x*	*y*	*z*	U_{eq}	*S.o.f.*
N1	2359(2)	−166(3)	2372(2)	48(1)	1
N2	1863(2)	−154(2)	3688(2)	39(1)	1
N3	7644(2)	209(2)	4306(2)	24(1)	1
N4	8117(2)	194(2)	3005(2)	23(1)	1
C1	1727(3)	450(4)	2329(3)	64(2)	1
C2	1551(4)	876(3)	2950(3)	64(2)	1
C3	1266(3)	456(3)	3561(3)	54(2)	1
C4	1572(3)	−783(3)	3629(2)	53(2)	1
C5	1309(4)	−801(4)	2948(3)	70(2)	1
C6	2037(3)	−786(4)	2397(3)	65(2)	1
C7	2621(3)	−120(2)	3867(2)	29(1)	1
C8	3240(3)	−685(2)	3936(2)	34(1)	1
C9	4028(3)	−634(2)	4048(2)	28(1)	1
C10	4285(2)	−11(2)	4107(2)	22(1)	1
C11	3637(3)	546(2)	4086(2)	30(1)	1
C12	2848(3)	502(2)	3977(2)	33(1)	1
C13	5128(2)	39(2)	4183(2)	21(1)	1
C14	5773(2)	−520(2)	4174(2)	25(1)	1
C15	6584(3)	−476(2)	4230(2)	26(1)	1
C16	6838(2)	150(2)	4292(2)	21(1)	1
C17	6190(2)	715(2)	4311(2)	24(1)	1
C18	5381(3)	655(2)	4263(2)	26(1)	1
C19	8326(3)	−359(2)	4368(2)	29(1)	1
C20	9032(2)	−364(2)	3806(2)	28(1)	1
C21	8755(2)	−377(2)	3140(2)	25(1)	1
C22	8360(3)	852(2)	3055(2)	30(1)	1
C23	7882(3)	1233(2)	3633(2)	33(1)	1
C24	7896(3)	871(2)	4294(2)	32(1)	1
C25	7400(2)	136(2)	2781(2)	24(1)	1
C26	6805(3)	707(2)	2625(2)	26(1)	1
C27	6032(3)	641(2)	2487(2)	30(1)	1
C28	5765(3)	18(2)	2478(2)	26(1)	1
C29	6386(3)	−542(2)	2560(2)	30(1)	1
C30	7163(3)	−495(2)	2701(2)	25(1)	1
C31	4906(3)	−37(2)	2397(2)	30(1)	1
C32	4293(3)	525(3)	2325(2)	39(1)	1
C33	3469(3)	489(3)	2298(2)	41(1)	1
C34	3190(3)	−135(3)	2344(2)	39(1)	1
C35	3814(3)	−710(3)	2379(2)	39(1)	1
N5	1913(2)	3734(2)	4494(2)	21(1)	1
N6	2463(2)	2401(2)	4375(2)	22(1)	1
N7	8145(2)	3084(2)	4908(2)	23(1)	1
N8	7582(2)	4365(2)	5190(2)	21(1)	1

C36	4633(3)	−654(2)	2401(2)	33(1)	1
C37	1259(2)	3570(2)	5022(2)	29(1)	1
C38	1053(2)	2886(2)	4960(2)	27(1)	1
C39	1816(3)	2329(2)	4935(2)	28(1)	1
C40	2158(2)	2484(2)	3731(2)	24(1)	1
C41	2143(3)	3180(2)	3422(2)	24(1)	1
C42	1667(2)	3751(2)	3835(2)	24(1)	1
C43	3291(2)	2364(2)	4434(2)	21(1)	1
C44	3897(2)	2413(2)	3874(2)	22(1)	1
C45	4713(2)	2447(2)	3933(2)	21(1)	1
C46	5007(2)	2435(2)	4551(2)	20(1)	1
C47	4411(2)	2330(2)	5089(2)	24(1)	1
C48	3599(3)	2288(2)	5039(2)	25(1)	1
C49	5844(2)	2537(2)	4618(2)	20(1)	1
C50	6403(2)	2747(2)	4089(2)	25(1)	1
C51	7160(2)	2905(2)	4174(2)	24(1)	1
C52	7442(2)	2861(2)	4793(2)	22(1)	1
C53	6924(2)	2586(2)	5312(2)	24(1)	1
C54	6159(2)	2438(2)	5225(2)	24(1)	1
C55	8726(2)	3345(2)	4378(2)	25(1)	1
C56	8974(2)	3995(2)	4547(2)	27(1)	1
C57	8233(2)	4556(2)	4678(2)	25(1)	1
C58	7867(2)	4219(2)	5833(2)	24(1)	1
C59	7936(3)	3488(2)	6048(2)	28(1)	1
C60	8433(2)	2999(2)	5551(2)	23(1)	1
C61	6789(2)	4347(2)	5090(2)	21(1)	1
C62	6181(2)	4194(2)	5622(2)	20(1)	1
C63	5398(2)	4127(2)	5526(2)	22(1)	1
C64	5125(2)	4191(2)	4897(2)	19(1)	1
C65	5713(2)	4367(2)	4384(2)	20(1)	1
C66	6503(2)	4437(2)	4470(2)	23(1)	1
C67	4291(2)	4104(2)	4800(2)	20(1)	1
C68	3702(2)	3925(2)	5320(2)	22(1)	1
C69	2918(2)	3822(2)	5234(2)	23(1)	1
C70	2656(2)	3883(2)	4607(2)	20(1)	1
C71	3236(2)	4086(2)	4086(2)	22(1)	1
C72	4005(2)	4184(2)	4183(2)	21(1)	1
C73	1843(3)	5378(2)	578(2)	32(1)	1
N9	2482(2)	4783(2)	649(2)	24(1)	1
N10	1894(2)	4783(2)	1942(2)	25(1)	1
N11	8125(2)	5340(2)	1432(2)	27(1)	1
N12	7600(2)	5193(2)	2746(2)	29(1)	1
C74	1592(3)	5777(2)	1191(2)	28(1)	1
C75	1296(3)	5384(2)	1804(2)	32(1)	1
C76	1610(3)	4155(2)	1859(2)	32(1)	1
C77	1439(3)	4080(2)	1161(2)	32(1)	1
C78	2191(3)	4139(2)	647(2)	31(1)	1
C79	2633(3)	4815(2)	2163(2)	26(1)	1
C80	3257(2)	4248(2)	2230(2)	26(1)	1
C81	4024(3)	4306(2)	2389(2)	28(1)	1

C82	4257(3)	4924(2)	2496(2)	26(1)	1
C83	3603(3)	5484(2)	2473(2)	27(1)	1
C84	2832(3)	5436(2)	2319(2)	29(1)	1
C85	5092(3)	4980(2)	2607(2)	25(1)	1
C86	5749(3)	4430(2)	2598(2)	29(1)	1
C87	6558(3)	4483(2)	2664(2)	29(1)	1
C88	6784(3)	5114(2)	2734(2)	26(1)	1
C89	6124(3)	5664(2)	2787(2)	29(1)	1
C90	5320(3)	5598(2)	2725(2)	31(1)	1
C91	8298(3)	4636(2)	2742(2)	33(1)	1
C92	9029(3)	4728(3)	2217(3)	49(2)	1
C93	8819(3)	4785(2)	1526(2)	31(1)	1
C94	8277(3)	6003(2)	1569(2)	29(1)	1
C95	7737(3)	6296(2)	2174(2)	33(1)	1
C96	7804(3)	5863(2)	2792(2)	32(1)	1
C97	7408(2)	5252(2)	1216(2)	23(1)	1
C98	6794(3)	5798(2)	1083(2)	25(1)	1
C99	6027(3)	5711(2)	935(2)	24(1)	1
C100	5800(2)	5079(2)	918(2)	20(1)	1
C101	6446(2)	4538(2)	1002(2)	24(1)	1
C102	7212(3)	4607(2)	1142(2)	28(1)	1
C103	4970(3)	5001(2)	815(2)	22(1)	1
C104	4339(2)	5545(2)	708(2)	23(1)	1
C105	3547(2)	5473(2)	636(2)	23(1)	1
C106	3289(2)	4841(2)	667(2)	22(1)	1
C107	3930(3)	4289(2)	740(2)	24(1)	1
C108	4734(3)	4359(2)	809(2)	26(1)	1
N13	1879(2)	8653(2)	−59(2)	32(1)	1
N14	2272(2)	7340(2)	322(2)	31(1)	1
N15	8068(2)	8027(2)	549(2)	34(1)	1
N16	7632(2)	9348(2)	255(2)	27(1)	1
C109	1244(3)	8708(2)	525(3)	41(1)	1
C110	1440(3)	8191(2)	1070(2)	40(1)	1
C111	1618(3)	7471(2)	879(2)	35(1)	1
C112	1968(3)	7192(2)	−284(2)	36(1)	1
C113	1318(3)	7742(2)	−511(2)	35(1)	1
C114	1610(3)	8404(2)	−638(2)	41(1)	1
C115	3100(3)	7336(2)	372(2)	28(1)	1
C116	3716(3)	7304(2)	−184(2)	29(1)	1
C117	4537(3)	7349(2)	−129(2)	28(1)	1
C118	4817(3)	7425(2)	475(2)	27(1)	1
C119	4199(3)	7417(2)	1026(2)	31(1)	1
C120	3382(3)	7372(2)	984(2)	31(1)	1
C121	5664(3)	7516(2)	515(2)	28(1)	1
C122	6269(3)	7515(2)	−39(2)	29(1)	1
C123	7064(3)	7641(2)	−24(2)	31(1)	1
C124	7324(3)	7817(2)	560(2)	29(1)	1
C125	6742(3)	7761(2)	1139(2)	32(1)	1
C126	5955(3)	7620(2)	1107(2)	30(1)	1

C127	8682(3)	8019(2)	−46(2)	37(1)	1
C128	8487(3)	8573(2)	−555(2)	37(1)	1
C129	8288(3)	9270(2)	−310(2)	33(1)	1
C130	7937(3)	9469(2)	868(2)	36(1)	1
C131	8609(3)	8917(3)	1065(3)	44(1)	1
C132	8347(3)	8229(3)	1140(2)	42(1)	1
C133	6827(3)	9320(2)	197(2)	24(1)	1
C134	6192(3)	9328(2)	743(2)	26(1)	1
C135	5397(3)	9254(2)	677(2)	27(1)	1
C136	5135(2)	9181(2)	70(2)	23(1)	1
C137	5763(2)	9197(2)	−481(2)	22(1)	1
C138	6557(3)	9265(2)	−422(2)	25(1)	1
C139	4296(2)	9090(2)	6(2)	22(1)	1
C140	3657(3)	9147(2)	553(2)	29(1)	1
C141	2864(3)	9039(2)	517(2)	29(1)	1
C142	2638(3)	8836(2)	−61(2)	29(1)	1
C143	3242(2)	8830(2)	−628(2)	26(1)	1
C144	4033(3)	8952(2)	−584(2)	29(1)	1
B1	2388(4)	2192(3)	6738(3)	45(2)	1
F11	3263(2)	1983(2)	6602(2)	79(1)	1
F12	2155(2)	2831(1)	6511(1)	61(1)	1
F13	2147(3)	2116(2)	7391(1)	70(1)	1
F14	2057(2)	1775(2)	6393(1)	63(1)	1
B2	189(3)	3667(2)	9772(2)	33(1)	1
F21	69(2)	4276(1)	9433(2)	51(1)	1
F22	1008(2)	3529(1)	9928(1)	46(1)	1
F23	71(2)	3170(1)	9386(1)	40(1)	1
F24	−379(2)	3681(2)	10333(2)	60(1)	1
B3	9804(3)	1468(2)	4829(2)	32(1)	1
F31	10392(2)	1687(2)	4349(2)	85(1)	1
F32	9870(2)	784(1)	4830(1)	42(1)	1
F33	9937(2)	1671(1)	5419(1)	48(1)	1
F34	9025(2)	1760(2)	4694(2)	79(1)	1
B4	9790(3)	241(3)	1449(2)	35(1)	1
F41	8946(2)	249(1)	1656(1)	38(1)	1
F42	9912(2)	236(2)	774(1)	69(1)	1
F43	10253(2)	−324(2)	1701(1)	57(1)	1
F44	10027(2)	806(2)	1650(2)	69(1)	1
B5	116(3)	5407(2)	3704(2)	32(1)	1
F51	244(2)	6065(2)	3736(2)	85(1)	1
F52	376(2)	5065(1)	4255(1)	35(1)	1
F53	535(3)	5113(3)	3172(2)	131(2)	1
F54	−729(2)	5436(2)	3708(2)	49(1)	1
B6A	7057(6)	2679(4)	2202(4)	59(3)	0.613(4)
F61A	6910(5)	3114(3)	1667(3)	87(1)	0.613(4)
F62A	7512(5)	2979(3)	2586(3)	87(1)	0.613(4)
F63A	6307(4)	2581(3)	2541(3)	87(1)	0.613(4)
F64A	7477(5)	2080(3)	1969(3)	87(1)	0.613(4)
B6B	7461(7)	2747(5)	2065(5)	59(3)	0.387(4)

F61B	6822(7)	2942(5)	1673(5)	87(1)	0.387(4)
F62B	7165(8)	2871(5)	2702(4)	87(1)	0.387(4)
F63B	8031(6)	3156(4)	1845(4)	87(1)	0.387(4)
F64B	7853(8)	2102(4)	2064(5)	87(1)	0.387(4)
B7	6673(5)	7737(3)	−1775(3)	77(3)	1
F71	6903(3)	7107(2)	−1518(2)	77(1)	1
F72	5835(3)	8010(2)	−1495(2)	117(2)	1
F73	6654(3)	7766(2)	−2403(2)	89(1)	1
F74	7106(3)	8163(2)	−1547(2)	106(2)	1
B8A	5484(6)	2648(5)	6944(4)	73(3)	0.528(3)
F81A	6124(5)	3039(4)	6980(4)	96(1)	0.528(3)
F82A	5884(6)	2049(3)	6715(4)	96(1)	0.528(3)
F83A	5069(6)	2593(4)	7548(3)	96(1)	0.528(3)
F84A	4870(5)	2942(4)	6551(3)	96(1)	0.528(3)
B8B	6268(6)	2525(5)	7148(5)	73(3)	0.472(3)
F81B	6024(7)	3080(4)	6713(4)	96(1)	0.472(3)
F82B	6891(6)	2090(4)	6782(4)	96(1)	0.472(3)
F83B	5594(6)	2271(4)	7475(4)	96(1)	0.472(3)
F84B	6667(6)	2826(4)	7552(4)	96(1)	0.472(3)
O1	1159(3)	6980(3)	2411(2)	76(2)	1
C145	−230(3)	6852(3)	2346(3)	54(2)	1
C146	423(3)	7132(3)	2614(2)	48(1)	1
C147	121(4)	7591(3)	3164(3)	72(2)	1
O2	192(3)	3167(2)	2863(2)	81(1)	1
C148	−489(5)	2251(3)	2871(3)	75(2)	1
C149	26(4)	2726(3)	2576(3)	55(2)	1
C150	211(8)	2695(6)	1872(5)	184(5)	1

Table S 8: Bond lengths [Å] and angles [°].

N1–C34	1.364(6)	C34–C35	1.426(7)
N1–C6	1.451(8)	C35–C36	1.368(6)
N1–C1	1.495(8)	N5–C70	1.355(5)
N2–C7	1.355(6)	N5–C42	1.478(5)
N2–C4	1.466(7)	N5–C37	1.486(5)
N2–C3	1.482(7)	N6–C43	1.364(5)
N3–C16	1.347(5)	N6–C39	1.477(5)
N3–C24	1.480(6)	N6–C40	1.482(5)
N3–C19	1.482(5)	N7–C52	1.362(5)
N4–C25	1.345(5)	N7–C60	1.468(5)
N4–C21	1.470(5)	N7–C55	1.486(5)
N4–C22	1.482(6)	N8–C61	1.347(5)
C1–C2	1.571(9)	N8–C58	1.473(5)
C2–C3	1.551(8)	N8–C57	1.481(5)
C4–C5	1.539(8)	C37–C38	1.514(6)
C5–C6	1.536(8)	C38–C39	1.538(6)
C7–C8	1.416(6)	C40–C41	1.525(6)
C7–C12	1.427(7)	C41–C42	1.530(6)
C8–C9	1.362(6)	C43–C48	1.410(6)
C9–C10	1.427(6)	C43–C44	1.430(5)
C10–C11	1.422(6)	C44–C45	1.370(6)
C10–C13	1.429(6)	C45–C46	1.431(6)
C11–C12	1.359(6)	C46–C47	1.409(5)
C13–C14	1.417(6)	C46–C49	1.445(5)
C13–C18	1.419(6)	C47–C48	1.363(6)
C14–C15	1.364(6)	C49–C54	1.419(6)
C15–C16	1.433(6)	C49–C50	1.423(5)
C16–C17	1.426(6)	C50–C51	1.368(6)
C17–C18	1.366(6)	C51–C52	1.419(6)
C19–C20	1.523(6)	C52–C53	1.426(6)
C20–C21	1.518(6)	C53–C54	1.374(6)
C22–C23	1.520(6)	C55–C56	1.531(6)
C23–C24	1.523(6)	C56–C57	1.528(6)
C25–C30	1.436(6)	C58–C59	1.525(6)
C25–C26	1.441(6)	C59–C60	1.535(6)
C26–C27	1.362(6)	C61–C66	1.423(6)
C27–C28	1.421(6)	C61–C62	1.438(5)
C28–C29	1.415(6)	C62–C63	1.353(5)
C28–C31	1.461(6)	C63–C64	1.432(6)
C29–C30	1.362(6)	C64–C65	1.406(5)
C31–C32	1.406(7)	C64–C67	1.446(5)
C31–C36	1.411(7)	C65–C66	1.360(6)
C32–C33	1.370(6)	C67–C72	1.417(6)
C33–C34	1.421(8)	C67–C68	1.418(5)
		C68–C69	1.371(6)
		C69–C70	1.420(6)
		C70–C71	1.428(5)

C71–C72	1.350(6)	N13–C142	1.356(6)
C73–N9	1.477(5)	N13–C109	1.481(6)
C73–C74	1.527(6)	N13–C114	1.483(6)
N9–C106	1.350(5)	N14–C115	1.370(6)
N9–C78	1.476(6)	N14–C111	1.466(5)
N10–C79	1.364(5)	N14–C112	1.483(6)
N10–C76	1.468(6)	N15–C124	1.354(6)
N10–C75	1.474(5)	N15–C132	1.475(6)
N11–C97	1.356(5)	N15–C127	1.479(6)
N11–C94	1.474(6)	N16–C133	1.348(5)
N11–C93	1.486(5)	N16–C129	1.475(5)
N12–C88	1.375(5)	N16–C130	1.481(6)
N12–C91	1.469(5)	C109–C110	1.531(7)
N12–C96	1.477(6)	C110–C111	1.520(7)
C74–C75	1.531(6)	C112–C113	1.511(7)
C76–C77	1.537(6)	C113–C114	1.510(7)
C77–C78	1.529(6)	C115–C116	1.421(6)
C79–C80	1.422(6)	C115–C120	1.423(6)
C79–C84	1.432(6)	C116–C117	1.380(6)
C80–C81	1.366(6)	C117–C118	1.422(6)
C81–C82	1.422(6)	C118–C119	1.420(6)
C82–C83	1.431(6)	C118–C121	1.441(6)
C82–C85	1.440(6)	C119–C120	1.368(6)
C83–C84	1.363(6)	C121–C122	1.410(6)
C85–C86	1.418(6)	C121–C126	1.420(6)
C85–C90	1.423(6)	C122–C123	1.371(6)
C86–C87	1.371(6)	C123–C124	1.426(7)
C87–C88	1.425(6)	C124–C125	1.439(6)
C88–C89	1.418(6)	C125–C126	1.377(6)
C89–C90	1.367(6)	C127–C128	1.526(7)
C91–C92	1.530(7)	C128–C129	1.513(7)
C92–C93	1.517(7)	C130–C131	1.516(7)
C94–C95	1.525(6)	C131–C132	1.534(7)
C95–C96	1.523(6)	C133–C134	1.426(6)
C97–C98	1.410(6)	C133–C138	1.435(6)
C97–C102	1.434(6)	C134–C135	1.360(6)
C98–C99	1.375(6)	C135–C136	1.412(6)
C99–C100	1.409(6)	C136–C137	1.430(5)
C100–C101	1.415(5)	C136–C139	1.439(6)
C100–C103	1.439(6)	C137–C138	1.351(6)
C101–C102	1.357(6)	C139–C144	1.414(6)
C103–C104	1.414(6)	C139–C140	1.430(5)
C103–C108	1.433(6)	C140–C141	1.364(6)
C104–C105	1.356(6)	C141–C142	1.411(6)
C105–C106	1.424(6)	C142–C143	1.429(6)
C106–C107	1.420(6)	C143–C144	1.375(6)
C107–C108	1.373(6)	B1–F13	1.367(6)

B1–F12	1.371(6)	C6–N1–C1	115.8(4)
B1–F14	1.372(6)	C7–N2–C4	122.9(4)
B1–F11	1.415(6)	C7–N2–C3	121.0(4)
B2–F24	1.384(5)	C4–N2–C3	116.1(4)
B2–F23	1.390(5)	C16–N3–C24	120.7(3)
B2–F21	1.392(5)	C16–N3–C19	124.1(3)
B2–F22	1.398(5)	C24–N3–C19	115.1(3)
B3–F34	1.370(5)	C25–N4–C21	123.2(4)
B3–F33	1.376(6)	C25–N4–C22	121.3(3)
B3–F32	1.387(5)	C21–N4–C22	115.2(3)
B3–F31	1.395(5)	N1–C1–C2	114.7(4)
B4–F41	1.384(5)	C3–C2–C1	110.7(6)
B4–F44	1.385(6)	N2–C3–C2	114.6(4)
B4–F43	1.387(6)	N2–C4–C5	109.1(5)
B4–F42	1.392(5)	C6–C5–C4	113.3(5)
B5–F53	1.337(6)	N1–C6–C5	111.0(5)
B5–F54	1.371(5)	N2–C7–C8	122.9(4)
B5–F52	1.385(5)	N2–C7–C12	121.0(4)
B5–F51	1.406(6)	C8–C7–C12	116.0(4)
B6A–F63A	1.365(9)	C9–C8–C7	121.9(4)
B6A–F64A	1.380(9)	C8–C9–C10	122.6(4)
B6A–F62A	1.397(10)	C11–C10–C9	114.5(4)
B6A–F61A	1.403(9)	C11–C10–C13	123.5(4)
B6B–F64B	1.366(11)	C9–C10–C13	122.0(4)
B6B–F62B	1.369(10)	C12–C11–C10	123.4(4)
B6B–F63B	1.370(11)	C11–C12–C7	121.2(4)
B6B–F61B	1.404(11)	C14–C13–C18	115.2(4)
B7–F73	1.308(7)	C14–C13–C10	122.6(4)
B7–F74	1.358(8)	C18–C13–C10	122.3(4)
B7–F71	1.376(7)	C15–C14–C13	123.0(4)
B7–F72	1.444(8)	C14–C15–C16	121.4(4)
B8A–F83A	1.350(10)	N3–C16–C17	121.6(4)
B8A–F82A	1.369(10)	N3–C16–C15	122.3(4)
B8A–F84A	1.413(10)	C17–C16–C15	116.0(4)
B8A–F81A	1.435(11)	C18–C17–C16	121.4(4)
B8B–F84B	1.367(11)	C17–C18–C13	123.0(4)
B8B–F83B	1.375(10)	N3–C19–C20	111.9(3)
B8B–F82B	1.403(10)	C21–C20–C19	114.1(3)
B8B–F81B	1.443(11)	N4–C21–C20	112.7(3)
O1–C146	1.212(6)	N4–C22–C23	113.6(3)
C145–C146	1.481(8)	C22–C23–C24	116.3(4)
C146–C147	1.502(8)	N3–C24–C23	113.0(4)
O2–C149	1.202(7)	N4–C25–C30	122.7(4)
C148–C149	1.447(9)	N4–C25–C26	121.8(4)
C149–C150	1.455(11)	C30–C25–C26	115.4(4)
C34–N1–C6	123.0(5)	C27–C26–C25	121.0(4)
C34–N1–C1	121.0(5)	C26–C27–C28	123.1(4)

C29–C28–C27	115.3(4)	C50–C51–C52	122.2(4)
C29–C28–C31	122.7(4)	N7–C52–C51	123.7(4)
C27–C28–C31	122.0(4)	N7–C52–C53	120.3(4)
C30–C29–C28	122.9(4)	C51–C52–C53	116.0(4)
C29–C30–C25	121.5(4)	C54–C53–C52	121.2(4)
C32–C31–C36	115.7(4)	C53–C54–C49	122.6(4)
C32–C31–C28	122.0(4)	N7–C55–C56	112.3(3)
C36–C31–C28	122.3(4)	C57–C56–C55	113.9(3)
C33–C32–C31	123.1(5)	N8–C57–C56	112.0(3)
C32–C33–C34	120.6(5)	N8–C58–C59	113.4(3)
N1–C34–C33	120.4(5)	C58–C59–C60	115.5(3)
N1–C34–C35	122.8(5)	N7–C60–C59	114.2(3)
C33–C34–C35	116.8(4)	N8–C61–C66	124.1(3)
C36–C35–C34	120.9(5)	N8–C61–C62	120.3(4)
C70–N5–C42	122.7(3)	C66–C61–C62	115.6(3)
C70–N5–C37	123.1(3)	C63–C62–C61	121.3(4)
C42–N5–C37	114.1(3)	C62–C63–C64	122.9(4)
C43–N6–C39	123.1(3)	C65–C64–C63	115.4(3)
C43–N6–C40	121.5(3)	C65–C64–C67	122.4(4)
C39–N6–C40	115.3(3)	C63–C64–C67	122.2(3)
C52–N7–C60	121.6(3)	C66–C65–C64	122.7(4)
C52–N7–C55	122.3(3)	C65–C66–C61	122.2(4)
C60–N7–C55	115.7(3)	C72–C67–C68	115.1(3)
C61–N8–C58	122.3(3)	C72–C67–C64	122.8(3)
C61–N8–C57	123.7(3)	C68–C67–C64	122.1(4)
C58–N8–C57	114.0(3)	C69–C68–C67	122.8(4)
C35–C36–C31	122.7(5)	C68–C69–C70	121.1(4)
N5–C37–C38	111.8(3)	N5–C70–C69	122.8(3)
C37–C38–C39	114.0(3)	N5–C70–C71	120.8(4)
N6–C39–C38	113.6(3)	C69–C70–C71	116.3(3)
N6–C40–C41	114.0(3)	C72–C71–C70	121.4(4)
C40–C41–C42	116.3(3)	C71–C72–C67	123.3(4)
N5–C42–C41	113.7(3)	N9–C73–C74	114.0(3)
N6–C43–C48	122.8(3)	C106–N9–C78	123.3(3)
N6–C43–C44	121.0(4)	C106–N9–C73	120.6(3)
C48–C43–C44	116.1(3)	C78–N9–C73	115.9(3)
C45–C44–C43	121.3(4)	C79–N10–C76	123.0(4)
C44–C45–C46	122.3(3)	C79–N10–C75	122.0(4)
C47–C46–C45	114.8(3)	C76–N10–C75	114.9(3)
C47–C46–C49	122.7(4)	C97–N11–C94	121.5(3)
C45–C46–C49	122.5(3)	C97–N11–C93	122.9(4)
C48–C47–C46	123.5(4)	C94–N11–C93	115.6(3)
C47–C48–C43	121.5(4)	C88–N12–C91	123.3(4)
C54–C49–C50	115.5(4)	C88–N12–C96	119.8(4)
C54–C49–C46	121.6(3)	C91–N12–C96	116.8(4)
C50–C49–C46	122.9(4)	C73–C74–C75	115.3(4)
C51–C50–C49	122.0(4)	N10–C75–C74	113.2(3)

N10–C76–C77	112.7(4)	C108–C107–C106	122.5(4)
C78–C77–C76	113.7(4)	C107–C108–C103	121.3(4)
N9–C78–C77	112.6(4)	C142–N13–C109	121.6(4)
N10–C79–C80	122.7(4)	C142–N13–C114	123.3(4)
N10–C79–C84	120.9(4)	C109–N13–C114	115.1(4)
C80–C79–C84	116.3(4)	C115–N14–C111	121.9(4)
C81–C80–C79	121.3(4)	C115–N14–C112	123.2(4)
C80–C81–C82	123.1(4)	C111–N14–C112	114.9(4)
C81–C82–C83	114.9(4)	C124–N15–C132	122.2(4)
C81–C82–C85	122.3(4)	C124–N15–C127	122.1(4)
C83–C82–C85	122.8(4)	C132–N15–C127	115.5(4)
C84–C83–C82	122.6(4)	C133–N16–C129	121.3(4)
C83–C84–C79	121.4(4)	C133–N16–C130	124.5(3)
C86–C85–C90	115.1(4)	C129–N16–C130	114.2(3)
C86–C85–C82	122.7(4)	N13–C109–C110	114.4(4)
C90–C85–C82	122.2(4)	C111–C110–C109	116.1(4)
C87–C86–C85	123.3(4)	N14–C111–C110	113.6(4)
C86–C87–C88	120.6(4)	N14–C112–C113	113.1(4)
N12–C88–C89	121.2(4)	C114–C113–C112	114.3(4)
N12–C88–C87	122.1(4)	N13–C114–C113	113.2(4)
C89–C88–C87	116.8(4)	N14–C115–C116	121.7(4)
C90–C89–C88	121.4(4)	N14–C115–C120	121.4(4)
C89–C90–C85	122.6(4)	C116–C115–C120	116.8(4)
N12–C91–C92	112.5(4)	C117–C116–C115	120.8(4)
C93–C92–C91	115.1(4)	C116–C117–C118	122.9(4)
N11–C93–C92	112.0(4)	C119–C118–C117	114.9(4)
N11–C94–C95	113.7(3)	C119–C118–C121	123.2(4)
C96–C95–C94	114.8(4)	C117–C118–C121	121.9(4)
N12–C96–C95	114.1(4)	C120–C119–C118	123.2(4)
N11–C97–C98	121.0(4)	C119–C120–C115	121.1(4)
N11–C97–C102	122.3(4)	C122–C121–C126	114.6(4)
C98–C97–C102	116.6(4)	C122–C121–C118	122.1(4)
C99–C98–C97	121.2(4)	C126–C121–C118	123.3(4)
C98–C99–C100	122.6(4)	C123–C122–C121	123.5(4)
C99–C100–C101	115.3(4)	C122–C123–C124	121.6(4)
C99–C100–C103	121.6(4)	N15–C124–C123	120.8(4)
C101–C100–C103	123.1(4)	N15–C124–C125	123.8(4)
C102–C101–C100	123.5(4)	C123–C124–C125	115.4(4)
C101–C102–C97	120.5(4)	C126–C125–C124	120.8(4)
C104–C103–C108	115.6(4)	C125–C126–C121	123.4(4)
C104–C103–C100	122.7(4)	N15–C127–C128	115.9(4)
C108–C103–C100	121.7(4)	C129–C128–C127	115.9(4)
C105–C104–C103	122.7(4)	N16–C129–C128	113.5(4)
C104–C105–C106	122.3(4)	N16–C130–C131	113.0(4)
N9–C106–C107	123.3(4)	C130–C131–C132	114.4(4)
N9–C106–C105	121.3(4)	N15–C132–C131	113.2(4)
C107–C106–C105	115.3(4)	N16–C133–C134	122.7(4)

N16–C133–C138	121.8(4)
C134–C133–C138	115.6(4)
C135–C134–C133	121.5(4)
C134–C135–C136	123.1(4)
C135–C136–C137	115.4(4)
C135–C136–C139	122.7(4)
C137–C136–C139	121.9(4)
C138–C137–C136	122.2(4)
C137–C138–C133	122.1(4)
C144–C139–C140	115.0(4)
C144–C139–C136	124.2(4)
C140–C139–C136	120.8(4)
C141–C140–C139	122.5(4)
C140–C141–C142	121.4(4)
N13–C142–C141	120.0(4)
N13–C142–C143	122.9(4)
C141–C142–C143	117.0(4)
C144–C143–C142	120.1(4)
C143–C144–C139	123.3(4)
F13–B1–F12	112.0(4)
F13–B1–F14	111.4(4)
F12–B1–F14	108.6(4)
F13–B1–F11	109.3(5)
F12–B1–F11	111.5(4)
F14–B1–F11	103.7(4)
F24–B2–F23	109.1(4)
F24–B2–F21	109.9(4)
F23–B2–F21	109.6(4)
F24–B2–F22	110.1(4)
F23–B2–F22	108.6(4)
F21–B2–F22	109.5(4)
F34–B3–F33	109.4(4)
F34–B3–F32	109.7(4)
F33–B3–F32	111.2(4)
F34–B3–F31	107.7(4)
F33–B3–F31	108.4(4)
F32–B3–F31	110.4(4)
F41–B4–F44	109.3(4)
F41–B4–F43	109.7(4)
F44–B4–F43	110.6(4)
F41–B4–F42	108.0(4)
F44–B4–F42	109.7(4)
F43–B4–F42	109.5(4)
F53–B5–F54	110.2(4)
F53–B5–F52	109.9(4)
F54–B5–F52	110.5(3)
F53–B5–F51	112.9(4)
F54–B5–F51	106.1(4)
F52–B5–F51	107.2(4)
F63A–B6A–F64A	108.6(7)
F63A–B6A–F62A	110.6(6)
F64A–B6A–F62A	113.4(8)
F63A–B6A–F61A	109.2(7)
F64A–B6A–F61A	107.9(6)
F62A–B6A–F61A	106.9(7)
F64B–B6B–F62B	105.5(9)
F64B–B6B–F63B	109.7(10)
F62B–B6B–F63B	109.1(10)
F64B–B6B–F61B	118.6(10)
F62B–B6B–F61B	110.4(10)
F63B–B6B–F61B	103.3(9)
F73–B7–F74	116.3(6)
F73–B7–F71	114.2(5)
F74–B7–F71	110.0(5)
F73–B7–F72	105.3(5)
F74–B7–F72	99.5(5)
F71–B7–F72	110.3(6)
F83A–B8A–F82A	112.6(8)
F83A–B8A–F84A	105.5(8)
F82A–B8A–F84A	111.0(8)
F83A–B8A–F81A	107.4(8)
F82A–B8A–F81A	106.4(8)
F84A–B8A–F81A	114.0(8)
F84B–B8B–F83B	112.1(9)
F84B–B8B–F82B	106.5(9)
F83B–B8B–F82B	117.7(9)
F84B–B8B–F81B	99.9(8)
F83B–B8B–F81B	112.7(9)
F82B–B8B–F81B	106.1(8)
O1–C146–C145	121.4(5)
O1–C146–C147	122.5(6)
C145–C146–C147	116.0(5)
O2–C149–C148	123.8(6)
O2–C149–C150	121.7(7)
C148–C149–C150	113.7(6)

Table S 9: Anisotropic displacement parameters [Å^2× 10^3]. The anisotropic displacement
factor exponent takes the form: $-2\pi^2[h^2a^{*2}U^{11} + ... + 2\, h\, k\, a^*\, b^*\, U^{12}]$.

Atom	U^{11}	U^{22}	U^{33}	U^{23}	U^{13}	U^{12}
N1	25(2)	93(4)	28(2)	−2(2)	−7(2)	−17(2)
N2	16(2)	74(3)	27(2)	−5(2)	−1(2)	−10(2)
N3	20(2)	30(2)	23(2)	−1(1)	−3(1)	−7(1)
N4	18(2)	26(2)	23(2)	3(1)	1(1)	−2(1)
C1	31(3)	122(6)	35(3)	10(3)	−15(2)	2(3)
C2	51(3)	69(4)	67(4)	4(3)	−14(3)	10(3)
C3	25(2)	91(5)	40(3)	1(3)	−8(2)	10(3)
C4	28(2)	96(4)	39(3)	−13(3)	5(2)	−30(3)
C5	58(3)	101(5)	62(4)	−6(3)	−13(3)	−38(3)
C6	38(3)	125(5)	38(3)	−26(3)	2(2)	−33(3)
C7	20(2)	44(3)	20(2)	−2(2)	0(2)	−3(2)
C8	29(2)	36(3)	38(2)	−11(2)	−2(2)	−12(2)
C9	24(2)	23(2)	39(2)	−7(2)	−7(2)	−4(2)
C10	25(2)	26(2)	12(2)	−2(2)	1(2)	−1(2)
C11	26(2)	27(2)	37(2)	−2(2)	−6(2)	0(2)
C12	24(2)	37(3)	36(2)	0(2)	−3(2)	2(2)
C13	21(2)	26(2)	13(2)	−2(2)	0(2)	−1(2)
C14	25(2)	24(2)	28(2)	−2(2)	−5(2)	−7(2)
C15	24(2)	26(2)	25(2)	−3(2)	−2(2)	5(2)
C16	22(2)	26(2)	16(2)	−2(2)	−1(2)	−5(2)
C17	27(2)	23(2)	25(2)	−4(2)	1(2)	−8(2)
C18	27(2)	17(2)	30(2)	−2(2)	2(2)	2(2)
C19	22(2)	37(3)	28(2)	7(2)	−7(2)	−5(2)
C20	14(2)	34(2)	37(2)	6(2)	−8(2)	−6(2)
C21	17(2)	24(2)	32(2)	0(2)	1(2)	1(2)
C22	25(2)	29(2)	35(2)	3(2)	2(2)	−9(2)
C23	29(2)	24(2)	46(3)	−5(2)	0(2)	−9(2)
C24	26(2)	35(2)	38(2)	−9(2)	1(2)	−13(2)
C25	25(2)	29(2)	16(2)	4(2)	4(2)	−4(2)
C26	27(2)	24(2)	27(2)	4(2)	−5(2)	−4(2)
C27	26(2)	35(3)	25(2)	4(2)	−1(2)	2(2)
C28	25(2)	33(2)	17(2)	5(2)	0(2)	−2(2)
C29	35(2)	27(2)	29(2)	2(2)	−7(2)	−6(2)
C30	23(2)	22(2)	27(2)	3(2)	−4(2)	5(2)
C31	27(2)	45(3)	20(2)	2(2)	−6(2)	−9(2)
C32	22(2)	61(3)	30(2)	15(2)	−7(2)	−4(2)
C33	28(2)	58(3)	33(2)	12(2)	−10(2)	2(2)
C34	20(2)	82(4)	18(2)	−2(2)	−4(2)	−12(2)
C35	33(2)	61(3)	27(2)	−3(2)	−5(2)	−19(2)
N5	17(2)	21(2)	26(2)	−4(1)	−3(1)	0(1)
N6	22(2)	18(2)	25(2)	0(1)	−2(1)	−3(1)

N7	18(2)	21(2)	30(2)	−1(1)	−5(1)	0(1)
N8	17(2)	19(2)	26(2)	−1(1)	−3(1)	−2(1)
C36	21(2)	50(3)	26(2)	−4(2)	−2(2)	−5(2)
C37	16(2)	37(2)	34(2)	−9(2)	3(2)	−8(2)
C38	12(2)	37(2)	31(2)	−1(2)	3(2)	−8(2)
C39	25(2)	34(2)	25(2)	5(2)	−2(2)	−12(2)
C40	22(2)	29(2)	22(2)	−2(2)	−6(2)	−7(2)
C41	26(2)	27(2)	24(2)	0(2)	−9(2)	−8(2)
C42	20(2)	27(2)	25(2)	4(2)	−8(2)	−5(2)
C43	21(2)	13(2)	30(2)	−2(2)	−4(2)	−4(2)
C44	28(2)	16(2)	22(2)	−1(2)	−6(2)	−4(2)
C45	23(2)	19(2)	20(2)	−4(2)	0(2)	−1(2)
C46	18(2)	13(2)	28(2)	−5(2)	−3(2)	2(2)
C47	24(2)	26(2)	22(2)	−2(2)	−5(2)	−4(2)
C48	29(2)	22(2)	23(2)	−2(2)	0(2)	−6(2)
C49	21(2)	13(2)	25(2)	−5(2)	−2(2)	2(2)
C50	25(2)	27(2)	24(2)	−9(2)	−6(2)	1(2)
C51	20(2)	25(2)	24(2)	−4(2)	3(2)	3(2)
C52	16(2)	15(2)	32(2)	−6(2)	−5(2)	7(2)
C53	23(2)	23(2)	26(2)	−1(2)	−5(2)	−1(2)
C54	23(2)	18(2)	30(2)	−2(2)	−3(2)	−1(2)
C55	16(2)	28(2)	30(2)	−7(2)	0(2)	−1(2)
C56	15(2)	30(2)	37(2)	−1(2)	−5(2)	−2(2)
C57	17(2)	26(2)	33(2)	2(2)	−6(2)	−5(2)
C58	14(2)	30(2)	30(2)	−6(2)	−9(2)	−4(2)
C59	19(2)	31(2)	33(2)	−3(2)	−5(2)	−5(2)
C60	19(2)	23(2)	29(2)	2(2)	−14(2)	1(2)
C61	18(2)	18(2)	24(2)	−1(2)	−4(2)	2(2)
C62	20(2)	16(2)	24(2)	0(2)	−5(2)	−2(2)
C63	18(2)	23(2)	23(2)	1(2)	−3(2)	−2(2)
C64	15(2)	13(2)	28(2)	−5(2)	−1(2)	2(2)
C65	19(2)	20(2)	21(2)	−2(2)	−5(2)	−4(2)
C66	21(2)	24(2)	21(2)	−1(2)	1(2)	−3(2)
C67	19(2)	14(2)	26(2)	−1(2)	−3(2)	1(2)
C68	24(2)	18(2)	22(2)	3(2)	−6(2)	−3(2)
C69	17(2)	25(2)	25(2)	0(2)	−1(2)	−2(2)
C70	18(2)	11(2)	30(2)	−4(2)	−2(2)	−1(2)
C71	24(2)	24(2)	19(2)	−2(2)	−6(2)	−2(2)
C72	18(2)	19(2)	26(2)	−4(2)	2(2)	−2(2)
C73	19(2)	43(3)	31(2)	10(2)	−3(2)	−4(2)
N9	23(2)	30(2)	21(2)	−1(1)	−5(1)	−5(2)
N10	17(2)	34(2)	24(2)	−2(2)	−2(1)	−1(1)
N11	22(2)	31(2)	26(2)	−5(2)	−5(1)	−1(2)
N12	28(2)	32(2)	26(2)	−3(2)	−7(2)	−2(2)
C74	20(2)	28(2)	35(2)	−2(2)	−4(2)	2(2)
C75	19(2)	41(3)	34(2)	−2(2)	−1(2)	−4(2)
C76	33(2)	33(2)	29(2)	3(2)	0(2)	−12(2)

C77	29(2)	38(3)	30(2)	0(2)	−4(2)	−11(2)
C78	31(2)	39(3)	27(2)	−6(2)	−1(2)	−17(2)
C79	23(2)	34(2)	19(2)	0(2)	2(2)	−5(2)
C80	25(2)	32(2)	20(2)	0(2)	1(2)	−9(2)
C81	28(2)	32(2)	22(2)	−1(2)	−2(2)	1(2)
C82	26(2)	32(2)	18(2)	−6(2)	−2(2)	−2(2)
C83	31(2)	26(2)	23(2)	−6(2)	−2(2)	−3(2)
C84	24(2)	30(2)	28(2)	−7(2)	−1(2)	3(2)
C85	26(2)	31(2)	15(2)	0(2)	0(2)	−3(2)
C86	31(2)	28(2)	26(2)	−6(2)	−2(2)	−1(2)
C87	22(2)	37(3)	27(2)	−1(2)	−4(2)	2(2)
C88	26(2)	34(2)	16(2)	−1(2)	−2(2)	−5(2)
C89	31(2)	35(2)	21(2)	−7(2)	0(2)	−4(2)
C90	33(2)	34(3)	22(2)	−5(2)	−1(2)	2(2)
C91	25(2)	39(3)	35(2)	3(2)	−10(2)	−1(2)
C92	37(3)	54(3)	54(3)	1(3)	−10(2)	0(2)
C93	22(2)	35(3)	34(2)	−4(2)	−3(2)	1(2)
C94	29(2)	27(2)	33(2)	−1(2)	−11(2)	−7(2)
C95	29(2)	31(2)	42(3)	−10(2)	−8(2)	−4(2)
C96	28(2)	37(3)	34(2)	−8(2)	−9(2)	−3(2)
C97	23(2)	29(2)	16(2)	−1(2)	−2(2)	−4(2)
C98	29(2)	19(2)	29(2)	3(2)	−5(2)	−6(2)
C99	27(2)	17(2)	28(2)	4(2)	−5(2)	−1(2)
C100	19(2)	26(2)	16(2)	3(2)	0(2)	−5(2)
C101	25(2)	20(2)	26(2)	−3(2)	−4(2)	−1(2)
C102	27(2)	24(2)	33(2)	−4(2)	−7(2)	0(2)
C103	31(2)	18(2)	16(2)	4(2)	0(2)	−4(2)
C104	27(2)	20(2)	23(2)	1(2)	−6(2)	−3(2)
C105	25(2)	24(2)	19(2)	3(2)	−1(2)	−2(2)
C106	25(2)	28(2)	14(2)	1(2)	−4(2)	−5(2)
C107	31(2)	19(2)	24(2)	2(2)	−4(2)	−9(2)
C108	28(2)	24(2)	25(2)	−3(2)	−3(2)	−2(2)
N13	23(2)	29(2)	44(2)	1(2)	−3(2)	−3(2)
N14	39(2)	23(2)	30(2)	−4(2)	0(2)	−9(2)
N15	29(2)	31(2)	41(2)	6(2)	−9(2)	−5(2)
N16	25(2)	29(2)	27(2)	−2(2)	−6(2)	−3(2)
C109	19(2)	36(3)	66(3)	−16(2)	6(2)	−5(2)
C110	31(2)	42(3)	46(3)	−13(2)	16(2)	−14(2)
C111	38(3)	34(3)	33(2)	−3(2)	7(2)	−12(2)
C112	37(2)	37(3)	36(2)	−10(2)	−2(2)	−13(2)
C113	27(2)	35(3)	45(3)	0(2)	−7(2)	−5(2)
C114	39(3)	38(3)	49(3)	9(2)	−17(2)	−8(2)
C115	31(2)	17(2)	36(2)	−5(2)	1(2)	−6(2)
C116	37(2)	21(2)	29(2)	−6(2)	−2(2)	−7(2)
C117	39(2)	20(2)	24(2)	0(2)	4(2)	−9(2)
C118	37(2)	15(2)	28(2)	0(2)	−1(2)	−4(2)
C119	37(2)	26(2)	29(2)	−4(2)	−4(2)	−7(2)

C120	40(3)	24(2)	27(2)	0(2)	2(2)	−7(2)
C121	37(2)	13(2)	34(2)	2(2)	−8(2)	−1(2)
C122	37(2)	23(2)	27(2)	3(2)	−4(2)	−5(2)
C123	30(2)	29(2)	31(2)	5(2)	4(2)	−4(2)
C124	31(2)	16(2)	37(2)	6(2)	−7(2)	3(2)
C125	36(2)	30(2)	31(2)	5(2)	−12(2)	−1(2)
C126	37(2)	22(2)	28(2)	7(2)	−3(2)	−1(2)
C127	25(2)	35(3)	48(3)	0(2)	−6(2)	2(2)
C128	31(2)	36(3)	39(3)	0(2)	5(2)	−5(2)
C129	21(2)	37(3)	38(2)	3(2)	3(2)	−5(2)
C130	30(2)	40(3)	39(3)	−5(2)	−7(2)	−3(2)
C131	36(3)	52(3)	46(3)	6(2)	−19(2)	−8(2)
C132	40(3)	46(3)	44(3)	12(2)	−23(2)	−8(2)
C133	24(2)	17(2)	29(2)	−2(2)	−5(2)	2(2)
C134	31(2)	23(2)	24(2)	−5(2)	−2(2)	−3(2)
C135	30(2)	28(2)	21(2)	2(2)	0(2)	−2(2)
C136	21(2)	15(2)	30(2)	−1(2)	0(2)	0(2)
C137	23(2)	17(2)	24(2)	1(2)	−3(2)	0(2)
C138	25(2)	23(2)	26(2)	−1(2)	−1(2)	1(2)
C139	23(2)	16(2)	24(2)	−1(2)	5(2)	0(2)
C140	34(2)	22(2)	27(2)	−4(2)	7(2)	−3(2)
C141	28(2)	25(2)	33(2)	−6(2)	5(2)	−3(2)
C142	28(2)	17(2)	41(2)	−4(2)	0(2)	−2(2)
C143	23(2)	24(2)	32(2)	−1(2)	−3(2)	−4(2)
C144	34(2)	21(2)	30(2)	−1(2)	−3(2)	0(2)
B1	56(4)	34(3)	37(3)	−2(3)	6(3)	−1(3)
F11	85(3)	77(3)	67(2)	11(2)	1(2)	−5(2)
F12	116(3)	28(2)	38(2)	2(1)	−11(2)	−7(2)
F13	129(3)	42(2)	27(2)	0(1)	9(2)	6(2)
F14	117(3)	33(2)	38(2)	0(1)	−18(2)	0(2)
B2	30(3)	30(3)	41(3)	−2(2)	−7(2)	−8(2)
F21	34(2)	35(2)	79(2)	10(2)	−1(1)	−4(1)
F22	43(2)	44(2)	58(2)	−2(1)	−28(1)	−10(1)
F23	38(1)	41(2)	46(2)	−3(1)	−21(1)	−8(1)
F24	70(2)	64(2)	50(2)	−11(2)	10(2)	−27(2)
B3	28(3)	33(3)	36(3)	−3(2)	−1(2)	−6(2)
F31	111(3)	92(2)	62(2)	−26(2)	26(2)	−71(2)
F32	40(2)	24(1)	61(2)	−5(1)	−7(1)	−1(1)
F33	61(2)	40(2)	45(2)	−4(1)	−16(1)	−12(1)
F34	69(2)	37(2)	141(3)	−26(2)	−64(2)	12(2)
B4	27(3)	49(3)	26(2)	1(2)	−5(2)	0(2)
F41	33(1)	48(2)	32(1)	−1(1)	−3(1)	−5(1)
F42	54(2)	103(3)	34(2)	14(2)	9(1)	18(2)
F43	53(2)	67(2)	46(2)	−4(2)	−20(1)	16(2)
F44	49(2)	63(2)	103(3)	−2(2)	−28(2)	−18(2)
B5	29(3)	36(3)	31(3)	7(2)	−5(2)	−8(2)
F51	77(2)	60(2)	136(3)	53(2)	−65(2)	−42(2)

F52	34(1)	37(2)	36(1)	6(1)	−11(1)	−10(1)
F53	128(3)	179(5)	34(2)	14(2)	22(2)	94(3)
F54	37(2)	59(2)	56(2)	5(2)	−20(1)	−12(1)
B6A	121(8)	29(4)	18(4)	4(3)	17(5)	−6(5)
F61A	155(3)	53(2)	45(1)	5(1)	−10(2)	−6(2)
F62A	155(3)	53(2)	45(1)	5(1)	−10(2)	−6(2)
F63A	155(3)	53(2)	45(1)	5(1)	−10(2)	−6(2)
F64A	155(3)	53(2)	45(1)	5(1)	−10(2)	−6(2)
B6B	121(8)	29(4)	18(4)	4(3)	17(5)	−6(5)
F61B	155(3)	53(2)	45(1)	5(1)	−10(2)	−6(2)
F62B	155(3)	53(2)	45(1)	5(1)	−10(2)	−6(2)
F63B	155(3)	53(2)	45(1)	5(1)	−10(2)	−6(2)
F64B	155(3)	53(2)	45(1)	5(1)	−10(2)	−6(2)
B7	133(7)	45(4)	53(4)	−14(3)	−31(4)	7(4)
F71	122(3)	43(2)	59(2)	−10(2)	−6(2)	5(2)
F72	155(4)	95(3)	67(3)	3(2)	27(3)	38(3)
F73	96(3)	130(4)	36(2)	−26(2)	−10(2)	4(3)
F74	183(4)	83(3)	75(3)	14(2)	−52(3)	−60(3)
B8A	119(8)	63(6)	42(5)	8(4)	14(5)	−50(6)
F81A	150(3)	65(2)	63(2)	−10(2)	15(2)	−7(2)
F82A	150(3)	65(2)	63(2)	−10(2)	15(2)	−7(2)
F83A	150(3)	65(2)	63(2)	−10(2)	15(2)	−7(2)
F84A	150(3)	65(2)	63(2)	−10(2)	15(2)	−7(2)
B8B	119(8)	63(6)	42(5)	8(4)	14(5)	−50(6)
F81B	150(3)	65(2)	63(2)	−10(2)	15(2)	−7(2)
F82B	150(3)	65(2)	63(2)	−10(2)	15(2)	−7(2)
F83B	150(3)	65(2)	63(2)	−10(2)	15(2)	−7(2)
F84B	150(3)	65(2)	63(2)	−10(2)	15(2)	−7(2)
O1	46(2)	125(4)	54(3)	2(3)	−1(2)	−14(2)
C145	52(3)	64(4)	44(3)	−17(3)	−18(2)	9(3)
C146	47(3)	59(3)	36(3)	9(2)	−4(2)	−13(3)
C147	83(4)	70(4)	70(4)	−20(3)	−22(4)	−10(4)
O2	95(3)	77(3)	83(3)	−10(2)	−9(3)	−47(3)
C148	95(5)	65(4)	66(4)	−10(3)	8(4)	−31(4)
C149	69(4)	52(3)	46(3)	−4(3)	−6(3)	−22(3)
C150	309(12)	177(8)	93(7)	−21(6)	45(8)	−168(8)

Table S 10: Hydrogen coordinates [× 10⁴] and isotropic displacement parameters [Å² × 10³].

Atom	*x*	*y*	*z*	U_{eq}	*S.o.f.*
H1A	1193	324	2246	77	1
H1B	1923	729	1952	77	1
H2A	2065	1046	3013	77	1
H2B	1109	1262	2889	77	1
H3A	1177	735	3944	65	1
H3B	721	329	3511	65	1
H4A	1090	–826	3965	63	1
H4B	2026	–1158	3694	63	1
H5A	1067	–1208	2923	84	1
H5B	867	–415	2886	84	1
H6A	2492	–1159	2466	77	1
H6B	1842	–842	1978	77	1
H8	3102	–1113	3902	40	1
H9	4421	–1028	4090	34	1
H11	3763	971	4151	36	1
H12	2440	893	3975	40	1
H14	5635	–945	4128	30	1
H15	6988	–869	4227	32	1
H17	6325	1141	4358	29	1
H18	4969	1043	4285	31	1
H19A	8096	–778	4381	35	1
H19B	8553	–337	4782	35	1
H20A	9294	35	3822	34	1
H20B	9465	–757	3865	34	1
H21A	9249	–383	2804	31	1
H21B	8527	–791	3112	31	1
H22A	8263	1119	2651	36	1
H22B	8967	790	3089	36	1
H23A	7291	1361	3555	39	1
H23B	8114	1646	3650	39	1
H24A	8468	819	4417	39	1
H24B	7513	1144	4622	39	1
H26	6953	1136	2617	32	1
H27	5654	1031	2393	36	1
H29	6256	–970	2515	36	1
H30	7556	–888	2747	30	1
H32	4458	950	2294	46	1
H33	3081	884	2249	49	1
H35	3660	–1137	2386	46	1
H36	5033	–1048	2419	39	1
H37A	746	3903	5009	35	1
H37B	1458	3592	5448	35	1
H38A	788	2885	4560	32	1
H38B	638	2791	5334	32	1

H39A	2071	2321	5341	33	1
H39B	1624	1898	4916	33	1
H40A	1585	2375	3780	28	1
H40B	2522	2163	3435	28	1
H41A	1892	3198	3013	29	1
H41B	2729	3255	3304	29	1
H42A	1765	4176	3609	29	1
H42B	1060	3736	3874	29	1
H44	3730	2423	3453	26	1
H45	5098	2479	3550	26	1
H47	4584	2286	5509	29	1
H48	3232	2207	5421	30	1
H50	6245	2780	3664	30	1
H51	7509	3048	3806	29	1
H53	7112	2503	5727	29	1
H54	5827	2264	5585	29	1
H55A	8455	3419	3977	30	1
H55B	9238	3010	4290	30	1
H56A	9267	3913	4937	33	1
H56B	9372	4137	4184	33	1
H57A	8436	4949	4812	30	1
H57B	7982	4681	4271	30	1
H58A	7470	4486	6158	28	1
H58B	8421	4357	5821	28	1
H59A	7363	3381	6156	33	1
H59B	8204	3421	6451	33	1
H60A	9030	3050	5501	28	1
H60B	8394	2542	5722	28	1
H62	6332	4139	6050	24	1
H63	5012	4034	5892	26	1
H65	5552	4440	3960	24	1
H66	6876	4551	4104	27	1
H68	3857	3875	5746	26	1
H69	2544	3707	5599	28	1
H71	3078	4153	3661	27	1
H72	4371	4314	3821	26	1
H73A	1337	5239	454	38	1
H73B	2061	5669	220	38	1
H74A	1138	6146	1109	34	1
H74B	2079	5974	1274	34	1
H75A	1203	5674	2180	38	1
H75B	752	5254	1755	38	1
H76A	1090	4129	2161	38	1
H76B	2042	3783	1975	38	1
H77A	1270	3642	1133	38	1
H77B	964	4424	1064	38	1
H78A	2657	3779	727	37	1
H78B	2037	4082	213	37	1
H80	3137	3822	2163	31	1
H81	4422	3914	2429	34	1

H83	3711	5906	2570	32	1
H84	2419	5822	2314	34	1
H86	5623	4003	2542	34	1
H87	6971	4096	2664	35	1
H89	6242	6087	2867	35	1
H90	4897	5979	2762	37	1
H91A	8093	4222	2669	40	1
H91B	8500	4587	3173	40	1
H92A	9243	5134	2304	59	1
H92B	9486	4348	2254	59	1
H93A	9323	4856	1224	37	1
H93B	8657	4364	1419	37	1
H94A	8167	6307	1190	34	1
H94B	8874	5975	1624	34	1
H95A	7898	6728	2244	40	1
H95B	7144	6382	2093	40	1
H96A	8382	5818	2901	39	1
H96B	7421	6088	3152	39	1
H98	6914	6235	1096	31	1
H99	5634	6091	841	29	1
H101	6337	4103	957	29	1
H102	7622	4225	1192	33	1
H104	4475	5980	685	28	1
H105	3151	5858	562	28	1
H107	3798	3855	740	29	1
H108	5142	3974	853	31	1
H10A	695	8667	396	49	1
H10B	1193	9156	697	49	1
H11Z	1932	8288	1253	48	1
H11Y	959	8243	1420	48	1
H11A	1791	7187	1256	42	1
H11B	1095	7345	773	42	1
H11C	1725	6779	–211	43	1
H11D	2450	7115	–631	43	1
H11E	1145	7610	–915	42	1
H11F	818	7793	–177	42	1
H11G	2084	8363	–993	49	1
H11H	1149	8733	–785	49	1
H116	3562	7252	–599	35	1
H117	4932	7327	–511	34	1
H119	4360	7443	1444	37	1
H120	2997	7365	1370	37	1
H122	6118	7423	–445	35	1
H123	7450	7609	–411	38	1
H125	6903	7823	1550	39	1
H126	5588	7590	1500	36	1
H12A	8727	7589	–250	44	1
H12B	9237	8043	83	44	1
H12C	8974	8550	–899	44	1

H12D	8005	8488	−758	44	1
H12E	8805	9393	−190	40	1
H12F	8105	9579	−667	40	1
H13A	7459	9519	1220	43	1
H13B	8164	9890	817	43	1
H13C	8772	9024	1482	52	1
H13D	9110	8901	734	52	1
H13E	8827	7898	1254	51	1
H13F	7887	8229	1504	51	1
H134	6328	9386	1163	31	1
H135	5000	9252	1056	32	1
H137	5618	9157	−901	26	1
H138	6950	9278	−803	30	1
H140	3790	9263	956	35	1
H141	2454	9103	889	35	1
H143	3097	8742	−1037	32	1
H144	4423	8944	−969	35	1
H14A	31	6562	1992	81	1
H14B	−630	7214	2180	81	1
H14C	−525	6596	2689	81	1
H14D	600	7748	3303	109	1
H14E	−163	7353	3529	109	1
H14F	−271	7970	3019	109	1
H14G	−582	2294	3343	112	1
H14H	−205	1801	2769	112	1
H14I	−1029	2335	2700	112	1
H15A	560	3028	1705	276	1
H15B	−314	2783	1680	276	1
H15C	511	2253	1758	276	1

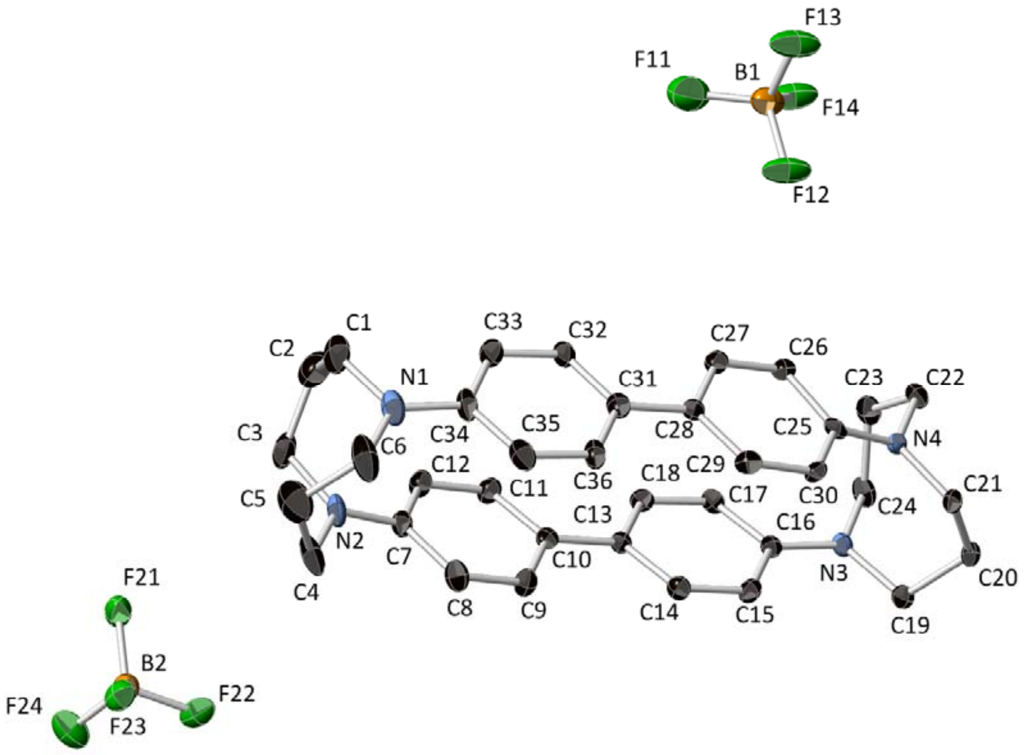

One cation anion pairing, thermal ellipsoids drawn at the 35% probability level. Solvent molecules, hydrogens and remaining anion/cations omitted for clarity.

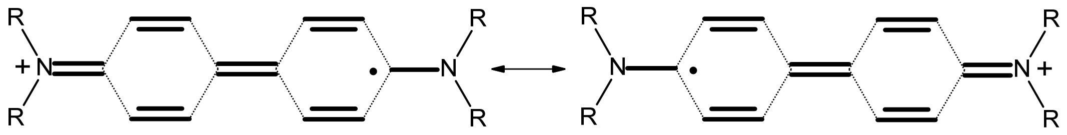

Figure S 16. Quinoidal resonance structure is indicated by alternation of bond lengths in the solid state. Relative bond lengths in the benzidine segments of **5** compared to benzidine (▬ = shorter, ⋯ = longer).

10 References

1. L. Mueller, *J. Magn. Reson.* **1987**, *72*, 191-196.
2. A. L. Davis, J. Keeler, E. D. Laue, D. Moskau, *J. Magn. Reson.* **1992**, *98*, 207-216.
3. R. E. Hurd, B. K. John, *J. Magn. Reson.* **1991**, *91*, 648-653.
4. R.Wagner, S. Berger, *J. Magn. Reson. A* **1996**, *123*, 119-121.
5. A. Bax, D. G. Davis, *J. Magn. Reson.* **1985**, *63*, 207-213.
6. L. Braunschweiler, R. R. Ernst, *J. Magn. Reson.* **1983**, *53*, 521-528.
7. (a) G. A. Morris, H.Barjat, In: *Analytical Spectroscopy Library*; Gy. Batta, K. E. Kövér, Cs. Szántay, Ed.s; Elsevier: Amsterdam, 1997; Vol. 8, pp 209-226.; (b) S. J. Gibbs, C. S. Johnson, *J. Magn. Reson.* **1991**, *93*, 395-402.
8. R. Mills, *J. Phys. Chem.* **1973**, *77*, 685-688.
9. J. E. Rickman, T. J. Atkins, *J. Am. Chem. Soc.* **1974**, *96*, 2268-2270.
10. A.Gogoll, H. Grennberg, A. Axén, *Organometallics* **1997**, *16*, 1167-1178.
11. R. D. Hancock, M. P. Ngwenya, P. W. Wade, J. C. A. Boeyens, S. M. Dobson, *Inorg. Chem.* **1990**, *29*, 264-270.
12. L. Börjesson, C. J. Welch, *Acta Chem. Scand.* **1991**, *45*, 621-626.
13. P. Caubére, N. Derozier, *Bull. Soc. Chim. Fr.* **1969**, 1737-1745.
14. a) N. S. Zefirov, S. V. Gogozina, *Tetrahedron* **1974**, *30*, 2345-2352; b) L. Toom, A. Kütt, I. Kaljurand, I. Leito, H. Ottosson, H. Grennberg, A. Gogoll, *J. Org. Chem.* **2006**, *71*, 7155-7164.
15. D. J. Brown, R. F. Evans, *J. Chem. Soc*, **1962**, 4039-4045.
16. J. H. Chapman, L. N. Owen, *J. Chem. Soc.* **1050**, 579-585.
